# Music therapy for supporting informal carers of adults with life-threatening illness pre- and post-bereavement; a mixed-methods systematic review

**DOI:** 10.1186/s12904-024-01364-z

**Published:** 2024-02-27

**Authors:** K. Gillespie, T. McConnell, A. Roulston, N. Potvin, C. Ghiglieri, I. Gadde, M. Anderson, J. Kirkwood, D. Thomas, L. Roche, M. O.’Sullivan, A. McCullagh, L. Graham-Wisener

**Affiliations:** 1 Centre for Improving Health-Related Quality of Life, School of Psychology, Queen’s University Belfast, Belfast, UK; 2 School of Nursing & Midwifery, Queen’s University Belfast, Belfast, UK; 3 Marie Curie Northern Ireland, Belfast, UK; 4 School of Social Sciences, Education & Social Work, Queen’s University Belfast, Belfast, UK; 5 Mary Pappert School of Music and School of Nursing, Music Therapy, Duquesne University, Pittsburgh, USA; 6 Cochrane Developmental, Psychosocial and Learning Problems, Centre for Public Health, Queen’s University Belfast, Belfast, Northern Ireland UK; 7 Independent Researcher, Belfast, Northern Ireland UK; 8 CHROMA Therapies, Overross House, Ross Park, Ross On Wye, Herefordshire, UK; 9 MusiCARER Project Carer Advisory Group, Belfast, UK; 10 AIIHPC Voices4Care, Dublin, Ireland; 11 Marie Curie Research Voices, Southampton, UK

**Keywords:** Palliative care, Music therapy, Bereavement, Grief, Carer, End-of-life

## Abstract

**Background:**

Music therapy interventions with informal carers of individuals with life-threatening illness at pre- and post-bereavement is an increasingly important clinical area. This systematic review is the first to synthesise and critically evaluate the international evidence associated with music therapy with adult informal carers pre- and post-bereavement. Specifically, the objectives were: i) to describe the characteristics and effectiveness of music therapy interventions which aim to improve health-related outcomes for adult informal carers of adults with life-threatening illness (pre- and post-bereavement), and ii) to describe the experience of music therapy for adult informal carers of adults with life-threatening illness (pre- and post-bereavement).

**Methods:**

Eligibility: adult informal carers of adults at end of life or bereaved; music therapy interventions for improving health-related outcomes; qualitative; mixed-method; and quantitative studies including comparators of any other intervention; published in English from 1998 onwards. Six databases were searched up to July 2022. A JBI mixed-methods systematic review approach was followed throughout, including quality appraisal, data extraction and a convergent segregated approach to synthesis and integration.

**Results:**

A total of 34 studies were included, published between 2003 and 2022. Most were conducted in North America (*n* = 13), Australia (*n* = 10), or Europe (*n* = 8). No studies were conducted in low- and middle-income countries or in the UK. The majority were qualitative (*n* = 17), followed by quasi-experimental (*n* = 8), mixed-methods (*n* = 7) and two RCTs. The majority focused on carers of individuals with dementia (*n* = 21) or advanced cancer (*n* = 7). Seventeen studies were purely quantitative or included a quantitative component. During meta-synthesis, findings were aligned to core outcomes for evaluating bereavement interventions in palliative care and previously identified risk factors for complicated grief. Commonly targeted outcomes in quantitative studies included quality of life and mental wellbeing, showing equivocal effectiveness of music therapy with significant and non-significant results. Twenty-two studies either purely qualitative or with a qualitative component underwent meta synthesis and suggested a diverse range of improved pre- and post-bereavement outcomes for informal carers across all core outcomes, and across all risk and protective factors, including psychological, spiritual, emotional, and social outcomes.

**Conclusions:**

Qualitative studies provide moderate to strong evidence for improved health-related outcomes for adult informal carers of adults with life-threatening illness pre-bereavement. Limited studies including those bereaved negates conclusions for the bereavement phase. Comparisons and explanations for effectiveness across quantitative and qualitative studies are equivocal, with a high risk of bias and small samples in the limited number of quantitative studies, demonstrating a need for high-quality RCTs.

**Systematic review pre-registration:**

PROSPERO [CRD42021244859].

**Supplementary Information:**

The online version contains supplementary material available at 10.1186/s12904-024-01364-z.

## Background

Music therapy is defined as the reflexive process of using music and sound by a professionally trained music therapist to cultivate therapeutic relationships and facilitate clinical interventions that promote optimal health, including physical, emotional, spiritual, and psychological wellbeing [1]. Music therapy has been used to support individuals in end-of-life care (EoLC) for more than forty years [2]. However, a recent service evaluation [3] with the professional body for music therapists in the UK identified that a high proportion (75.5%) of music therapists working in EoLC also focus their therapeutic practice on informal carers (family members or friends providing physical and/or psychosocial support. In this context, music therapists provide dyadic, family-based or informal carer interventions both pre- and post-bereavement [3] i.e. during the period before or after the patient’s death. These findings indicate clinical recognition for the value of music therapy as a form of support for informal carers across the bereavement continuum. There are a number of evidence-based reasons why this may be the case.

Arts-based approaches may align particularly well to the internationally recognised need for public health approaches to bereavement support [4, 5]. Within a public health approach to bereavement support there is acknowledgement of the need to avoid pathologising grief, instead emphasising a resilience-based approach including social support through an appropriate mix of universal, selective and specialist bereavement support services [4, 5]. This is reflected in the recently developed core outcome set for evaluating bereavement interventions [6], with the two outcomes – “Ability to cope with grief” and “Quality of life and mental wellbeing”— reflecting a departure from disease-focused outcomes in previous bereavement research. Here, bereavement is less an experience to be extinguished and rather one to be honoured as a space for deriving meaning from loss. The role of the arts in providing holistic care that enhances multiple dimensions of quality of life and addresses complex problems for which there are not currently adequate solutions was recently highlighted in a WHO evidence synthesis [7]. Arts-based approaches, and music therapy within this, offer a creative means for self-expression and reflection, and are often centred upon developing social connections and deriving meaning from experiences [8].

Music therapy may usefully target risk and protective factors for complicated grief. Although the majority of bereaved individuals will adjust psychologically with time [9], a subgroup of individuals will experience complicated grief [10]. This is a Complex or Prolonged Grief Disorder (PGD [11], associated with prolonged impairment of psychological and social functioning [12, 13]. Evidence from a gap analysis indicates components of existing bereavement support interventions rarely map to the modifiable risk and protective factors for PGD [14]. Examples of risk factors include depression [15], anxiety [16], family conflict at EoL [17], poor perceived social support [18], early non-acceptance of loss [19], Post-Traumatic Stress Disorder (PTSD) [20] and difficulty accessing positive memories [14]. Examples of protective factors include higher spirituality [21], satisfaction with palliative care [21], working part-time [22], and perceived preparedness for death [21]. A realist evaluation to develop an understanding of how music therapy works to improve patient outcomes in EoLC [8] identified several mechanisms of change which align with the above determinants. Examples include music therapy helping individuals reframe their identities from patients to people with unique pasts, helping patients reconnect with happier memories, to safely express repressed emotions, and to transcend to a higher plane. It is an open question as to whether similar mechanisms to those identified in patients in EoLC may also facilitate improvement in outcomes for informal carers.

Music therapy is a form of support which could be notably applicable across the bereavement continuum. There is increasing recognition that bereavement support needs are to be considered before, during and after a close person dies [23]. Pre-bereavement is the period before the death of a close person, and is when some individuals may experience anticipatory grief [24]. Although there is no consensus definition of anticipatory grief, it is generally agreed upon to be the carer’s reaction to the perception of multiple losses in the caregiving period of their loved ones with a life-threatening illness or when their loved ones are approaching death, beginning as early as diagnosis [25, 26]. Recognition that grieving processes can begin at the point of diagnosis aligns to a public health approach to bereavement support, with acknowledgement of the importance of pre-bereavement support in various clinical guideline and quality standard documents (e.g. [27, 28]). Music therapy is currently being used in clinical practice to support carers before the death of a loved one [3]. This may be as it is a readily adaptable therapeutic approach, and can be delivered effectively to both individuals, dyads and families, depending on the context. In addition to the mechanisms to preventing PGD already discussed, many of which are applicable pre-bereavement, music therapy in EoLC is evidenced to strengthen social bonds with loved ones and to provide ongoing connections after death [8]. This suggests that even when applied only at pre-bereavement, there may be longer-term benefits to the grieving process from music therapy.

Although music therapy with adult informal carers of individuals at end-of-life is an emergent clinical area, evidence for music therapy intervention in this context is limited (e.g. [29–31]) and largely focused on bereaved children and adolescents [32–34]. Despite anecdotal evidence, there is a lack of strong research evidence for music therapy’s effectiveness in this area, and a lack of understanding around the mechanisms through which music therapy may influence outcomes, and how music therapy is experienced by adult informal carers. The lack of translation to evidence-informed practice limits the ability of music therapists working in this area to maximise best practice and avoid the potential for harm. A further consequence of the limited evidence base is that music therapy is not referenced in EoLC guidelines [35] and rarely funded as a core service in the UK National Health Service (NHS) [3], thus creating barriers for carers and patients to gain access to music therapy services. We know that improving availability of bereavement care in routine practice is a priority research area within End-of-Life Care (EoLC), as identified by 1,403 patients, carers and healthcare professionals as part of a James Lind Alliance Priority Setting Partnership Exercise [36]. We also know the small number of bereavement support randomized controlled trials that do exist have methodological limitations which reduce confidence in the findings [37]. This includes small sample sizes and heterogeneity in study populations, models of care, and outcomes. There is an opportunity to develop evidence-based music therapy interventions to be implemented as part of the bereavement support offering. However, the first step is identifying and critically reviewing the existing evidence base for this therapeutic approach.

A preliminary search of PROSPERO, MEDLINE, The Open Science Framework (OSF), the Cochrane Database of Systematic Reviews, and the JBI Database of Systematic Reviews and Implementation Reports was conducted and no current or underway systematic reviews on the topic were identified. Music therapy interventions within palliative care settings have been included in a number of systematic reviews (e.g. [38–40]). However, these reviews were limited in their focus on patient outcomes, quantitative evidence, and often particular research designs (e.g. randomized controlled trials). A recent systematic review of bereavement interventions [37] included relevant studies but was limited to post-bereavement interventions and to the UK or comparable countries where the research is likely to be applicable to the UK. An examination of the international evidence base for the value of music therapy across the bereavement continuum is important, given increasing evidence for the important role of the pre-bereavement period in reducing negative outcomes such as PGD [4, 5, 14]. To date, no previous reviews have comprehensively examined the range of international evidence associated with music therapy with adult informal carers pre- and post-bereavement.

## Aims of review

This mixed-methods segregated systematic review is the first to synthesise and critically evaluate the current state of the international evidence base for music therapy with adult informal carers of individuals with life-threatening illness at pre- and post-bereavement. Inclusion of both qualitative and quantitative research designs will identify a comprehensive range of evidence relating to efficacy and experience of music therapy.

Specifically, the objectives are:

- To describe the characteristics (including mechanisms of change, implementation processes, and economic considerations) and effectiveness of music therapy interventions which aim to improve health-related outcomes for adult informal carers of adults with life-threatening illness (pre- and post-bereavement)
- To describe the experience of music therapy for adult informal carers of adults with life-threatening illness (pre- and post-bereavement)

Review questions

i. What are the characteristics (including mechanisms of change, implementation processes and economic considerations) of music therapy interventions which aim to improve health-related outcomes for adult informal carers of adults with life-threatening illness (pre- and post-bereavement)?
ii. What is the effectiveness of music therapy interventions which aim to improve health-related outcomes for adult informal carers of adults with life-threatening illness (pre- and post-bereavement)?
iii. What is the experience of adult informal carers of adults with life-threatening illness (pre- and post-bereavement) receiving music therapy?

## Methods

The systematic review was conducted in accordance with the Joanna Briggs Institute (JBI) methodology for a segregated Mixed-Methods Systematic Review [41] and is reported according to the PRISMA 2020 Statement [42]. The protocol was pre-registered via the International Prospective Register of Systematic Reviews PROSPERO [43].

### Inclusion criteria

#### Population

This review is interested in the potential of music therapy as a psychosocial-spiritual intervention with adult informal carers across the bereavement continuum (pre- and post-bereavement). The population of interest, therefore, was informal carers who are close persons (e.g. spouse, adult children, parent, sibling, relative, friend, or neighbour) of an individual diagnosed with a life-threatening illness (advanced, progressive, incurable) approaching end-of-life (pre-bereavement), or an informal carer who is bereaved. Both the informal carer and individual diagnosed with a life-threatening illness needed to be adults (≥ 18 years old). There were no restrictions on gender or ethnicity.

The authors recognise that there is no agreement on when the pre-bereavement period begins, and so define this as informal carers providing care to individuals with a life-threatening illness approaching EoL using the NICE “End of Life Care for Adults” guideline [35]. In line with the guidelines, conditions such as dementia are included where the prognosis and disease trajectory are unpredictable and where people may reach end-of-life before they are in the advanced stages of disease [44].

People are considered to be approaching the end of life when they are likely to die within the next 12 months, although this is not always possible to predict. This includes people whose death is imminent, as well as people who:

- have an advanced incurable illness, such as cancer, dementia or motor neurone disease
- are generally frail and have co-existing conditions that mean they are expected to die within 12 months
- have existing conditions if they are at risk of dying from a sudden crisis in their condition
- have a life-threatening acute condition caused by a sudden catastrophic event, such as an accident, stroke or medical complications.

There was no restriction on the setting. Studies with adults bereaved through unexpected sudden deaths with no life-threatening condition as outlined above (e.g. suicide, homicide, natural disaster, terrorist activity, road traffic collision, plane crash) were excluded.

Studies of mixed populations where the sample is composed ≥ 50% of the target population were eligible. Otherwise, studies were included where participants’ data were separately reported and could be extracted.

#### Intervention

Music therapy interventions aimed at improving adult informal carer health-related outcomes. This included interventions which i) primarily target patients but record outcomes for carers, ii) are dyadic/family-based (involving at least one adult), or iii) focused primarily on carers (current/bereaved). Music therapy is defined as “the use of sounds and music within an evolving relationship between client and therapist to support and encourage physical, mental, social, spiritual and emotional wellbeing” [1]. This refers to tailored interventions delivered in an individual/group setting by professionally trained music therapists or trainees in a professional music therapy training programme. This can involve a number of processes as detailed below, however a clear therapeutic process needed to be present.

- Receptive methods that structure active and intentional listening experiences with pre-composed music via live performance or recordings.
- Recreative methods that draw upon pre-composed music to structure active musicking through instrumental play, singing, movement/dancing and other forms of creative engagement.
- Improvisational methods that structure spontaneous musicking using instruments (percussive and melodic) and singing.
- Compositional methods that facilitate composition of new music, including but not limited to lyrics, melodies, harmonies, and rhythms.

Interventions with any mode of delivery were eligible, such as face‐to‐face contact, telephone, or online interventions, and we included individual or group-based interventions. There were also no restrictions on the duration or frequency of interventions.

Some studies were included in initial screening but on closer inspection of the full text did not meet the criteria as music therapy interventions. For example, Canga et al. [45] investigated “environmental music therapy”, but this intervention was determined to lack the therapeutic process necessary to be considered a music therapy intervention. Similarly, the intervention assessed by Dassa et al. [46], involved music therapists in choosing music, but lacked a therapeutic process as music was used as an added element, rather than a central therapeutic medium.

#### Comparator

The quantitative component of the review considered studies that compared music therapy to any other intervention, examples including; bereavement support and social groups, education, psychological support and counselling, befriending and home-visiting support, arts-based approaches, spiritual approaches, complementary therapies, or pharmacological therapies. Interventions comparing music therapy to usual care (i.e., as provided by the multidisciplinary team in any care setting) were also eligible. Aligned to an inclusive approach regarding study design, studies without a comparison group were also eligible.

#### Outcomes

The quantitative component of this review considered studies that included any health-related outcome for the informal carer, as defined broadly using the Dodd et al.’s [47] taxonomy of outcomes in medical research. This included but was not limited to outcomes of life impact (e.g., health-related quality of life, carer burden, coping, emotional/wellbeing, delivery of care), resource use, adverse events, physiological/clinical and mortality/survival.

Outcomes could be measured using any validated instrument (via observation, clinician-administered or self-report) and be measured during or immediately after the intervention or at a follow-up period. Regarding the hierarchy of outcome measures, clinician-administered measures were prioritised and where multiple outcome measures of the same type were used, the outcome measure that was most frequently used across the included studies was prioritised.

#### Phenomena of interest

The qualitative component of this review considered studies that explored the experiences of adult informal carers of music therapy intervention as defined above.

#### Context

The qualitative component of this review considered studies from all geographical regions. It also included all contexts (e.g., primary care, secondary, tertiary, community, or home settings).

#### Types of studies

This review considered primary quantitative, qualitative and mixed-methods studies. Quantitative studies included both experimental and quasi-experimental study designs, including randomised controlled trials, non-randomised controlled trials, before and after studies, and interrupted time-series studies, analytical observational studies (including prospective and retrospective cohort studies), case–control studies, and analytical cross-sectional studies. Qualitative studies include, but are not limited to, designs such as phenomenology, grounded theory, ethnography, qualitative description, action research, and feminist research. Mixed-method studies were only considered if relevant data from the quantitative or qualitative components could be clearly extracted.

Studies could be conducted in any country, however, only studies published in English were included (due to lack of resources for translation).

Studies had to be empirical and published in peer-reviewed journals, with a publication year from 1998 onwards. This date reflects the formal establishment of key music therapy professional bodies and the formal adoption of music therapy as a protected title in the US and UK. In the absence of sufficient research studies, grey literature was to be considered (e.g. conference papers and doctoral theses), however this was assessed as not needed.

Systematic reviews were not included, however relevant studies were harvested from them, where relevant. Editorials, opinion papers, case studies (case series or case reviews), and any articles without relevant, original data were excluded.

### Search strategy

An initial limited search of MEDLINE and PsycINFO was undertaken to identify articles on the topic using the following keywords: Music therapy AND carer OR caregiver OR bereaved AND palliative OR end of life OR advanced illness. We used text words and indexing terms from relevant records to develop a comprehensive set of search terms for each concept. The final strategy for MEDLINE was constructed in collaboration with an information specialist (MA) using two separate strands combined with OR (music therapy AND carers) OR (music therapy AND end of life). This sensitive approach reduced the risk of missing studies which were not primarily focused on carers, but which were relevant to the review question. The MEDLINE search strategy was adapted for each database using appropriate syntax and indexing terms.

### Information sources

We searched the following databases on 15 April 2021 and updated the search on 19 July 2022. MEDLINE All (Ovid).

- APA PsycINFO (Ovid)
- Embase (OVID)
- CINAHL Plus (EBSCOhost)
- RILM Abstracts of Music Literature (EBSCOhost)
- Cochrane Central Register of Controlled Trials (CENTRAL)

The searches were limited to English language publications and by year of publication (1998 onwards), See Additional file 1 for a record of the database searches and the complete search strategies.

In addition, we checked reference lists of reviews and of included articles for additional studies and performed citation searches on included articles. We also contacted international research leaders in the field for additional studies. This included the corresponding author of all included studies, in addition to those within the professional network of the research team.

### Study selection

All records from the electronic searches were imported into EndNote X9.3.3 [48] and duplicates removed. The remaining records were exported to Rayyan [49], with titles and abstracts independently screened by two reviewers for assessment against the inclusion criteria for the review (LGW, TMcC, IG, KG). Potentially relevant studies were retrieved in full, and their citation details imported into Rayyan [49]. If a full text paper could not be identified, the relevant authors were contacted. The full text of selected citations were also independently assessed in detail against the inclusion criteria by two independent reviewers (LGW, TMcC, IG, KG, CG). Reasons for exclusion of papers at full text stage were recorded. Any disagreements that arose between the reviewers at each stage of the selection process were resolved through discussion, or with additional review and discussion with an additional reviewer/s (LGW, TMcC, IG, KG, CG).

### Assessment of methodological quality

All papers were assessed by two independent reviewers (LGW, TMcC, KG, AR, NP) for methodological quality prior to data extraction. Quantitative papers (and the quantitative component of mixed-methods papers) were assessed using standardised critical appraisal instruments from JBI for randomised controlled trials and quasi-experimental studies [50]. Qualitative papers (and the qualitative component of mixed-methods papers) were assessed using the standardised critical appraisal instrument from JBI for qualitative research [51]. Authors of papers were contacted to request missing or additional data for clarification, where required. Any disagreements that arose between the reviewers were resolved through discussion, or with a third reviewer. All studies, regardless of the results of their methodological quality, underwent data extraction and synthesis (where possible) with quality appraisal helping to indicate the strength of evidence.

### Data extraction

Included studies were divided equally among the review team for independent data extraction (LGW, TMcC, KG, AR, NP) using a modified version of the JBI data extraction tool. Data extraction was then independently checked by another member of the same data extraction team for accuracy and completeness. The data extraction tool captured data on the characteristics of included studies, including the methods, participants, analysis and findings for both quantitative studies and qualitative studies, as well as the key conclusions of the study authors and references to other relevant studies. The tool was modified for the current study with integration of the Reporting Guidelines for Music-based Interventions [52], to enable detailed information on intervention content to be extracted.

For quantitative studies, data were extracted for any health-related outcome relating to the carer. Outcome data were extracted for all relevant measures, and at all time-points. For qualitative studies, findings and their illustrations were extracted and assigned a level of credibility. A finding is defined by the JBI as “a verbatim extract of the author’s analytic interpretation accompanied by either a participant voice, or fieldwork observations or other data.” ([53], p.40). Findings were identified through repeated reading of the text, with extraction of findings including any distinct analytic observation with an accompanying illustration. Each study finding was identified by an alphanumeric code (e.g. A1, A2, B1…), with the letter corresponding to a study and each number to a unique finding. Each finding was rated with one of three levels of credibility:

1. Unequivocal—findings accompanied by an illustration that is beyond reasonable doubt and therefore not open to challenge.
2. Credible—findings accompanied by an illustration lacking clear association with it and therefore open to challenge.
3. Not Supported—findings are not supported by the data.

Any disagreements that arose between the reviewers were resolved through discussion, or with a third reviewer. Authors of papers were contacted to request missing or additional data, where required.

### Data synthesis and integration

This review followed a convergent segregated approach to synthesis and integration according to the JBI methodology for MMSR [42]. This involved separate quantitative and qualitative synthesis followed by integration of the resultant quantitative evidence and qualitative evidence.

### Quantitative synthesis

The included studies were assessed for their suitability for meta-analysis. Significant heterogeneity was found in relation to the clinical population, the carer support, the study design, and outcomes measured. The majority of studies were also quasi-experimental, with meta-analysis not advised [54] due to threats to validity. Instead, the findings are presented in narrative form including tables and figures to aid in data presentation, where appropriate. The narrative is structured in line with the Reporting guidelines for music-based interventions [52] and outcomes are mapped where relevant against previously identified predictors of PGD [14].

### Qualitative synthesis

Qualitative research findings were pooled with the meta-aggregation approach [51]. This involved the aggregation or synthesis of findings (unequivocal or credible) to generate a set of statements that represent that aggregation, through assembling the findings (Level 1 findings) rated according to their quality, and categorising these findings based on similarity in meaning (Level 2 findings). At the Level 2 stage, these findings were also mapped to existing risk and protective factors for complicated grief in order to build on existing knowledge and ensure relevance of findings. For example, “Synthesised finding 1: Social connectedness and social support” was most closely aligned with the existing risk factor, “poor perceived social support”. These categories were then subjected to a meta-synthesis to produce a single comprehensive set of synthesised findings (Level 3 findings) that can be used as a basis for evidence-based practice.

### Integration of quantitative evidence and qualitative evidence

The findings of each single method synthesis included in this review were configured according to the JBI methodology for mixed-methods systematic reviews [41]. This involved quantitative evidence and qualitative evidence being juxtaposed together and organised/linked into a line of argument to produce an overall configured analysis.

## Results

### Study inclusion

**Fig. 1:**
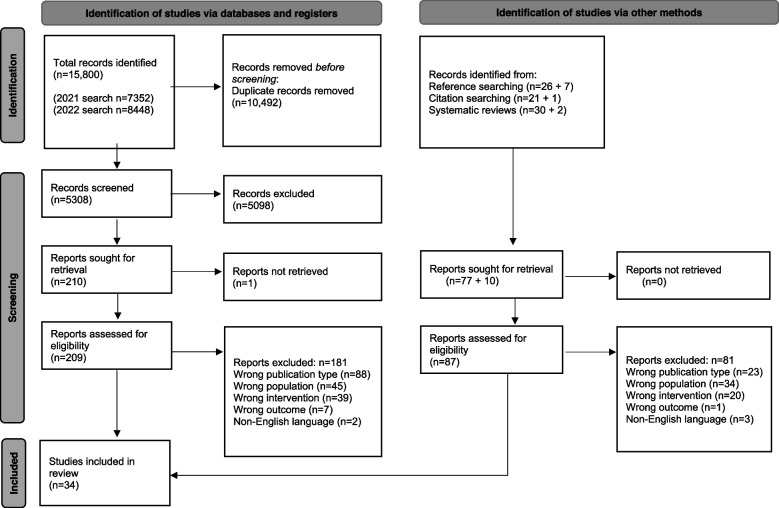
PRISMA 2020 study flow diagram [55]

The database searches yielded 15,800 records. After removal of duplicates, 5308 titles and abstracts were screened for relevance and 5098 were excluded. The full texts of 209 papers were assessed against the inclusion criteria. From these, we identified 28 studies for inclusion, and 181 were excluded with reasons. Four additional included studies were identified from supplementary searches of reference lists. A total of 34 studies were included in the review. A study flow diagram showing the number of studies at each stage of the screening process and reasons for exclusion is presented in Fig. 1.

Any studies which were sent to the team after contacting experts had already been identified through database searches. No further studies were included in this way.

### Study characteristics

The 34 studies included in the review were published between 2003 and 2022. There is evidence that this is an emerging research area, with the majority of studies (*n* = 20) published in the last 5 years. The studies were largely conducted in the US (*n* = 11) or Australia (*n* = 10), followed by European countries (*n* = 8) or Canada (*n* = 2). Only three studies were conducted outside of North America/Europe, in Israel [56] and South Korea [57, 58]. No studies were conducted in low- and middle-income countries (LMICs) or in the UK. In relation to research design, the majority of studies were qualitative (*n* = 17), with purely quantitative studies including quasi-experimental designs (*n* = 8) and two RCTs [58, 59]. Mixed-methods studies (*n* = 7) were quasi-experimental in design, with a qualitative component.

Carers were defined as members of the individuals family in the majority of studies (*n* = 27), and in a smaller number of studies either family or friends (*n* = 4) or the patient’s spouse (*n* = 3). Carers supported a range of different patient populations. The majority of studies focused on carers of individuals with dementia (*n* = 21) or advanced cancer (*n* = 7). A smaller number of studies focused on carers of individuals within an end-of-life population broadly [31, 58, 60, 61], mixed-neurological conditions [62] or Parkinson’s Disease [63].

### Music therapy intervention description

Music therapy interventions were delivered to patient-carer dyads in the majority of studies (*n* = 23), with a smaller number of studies reporting on music therapy interventions delivered to the carer only (*n* = 6) or primarily to the patient (*n* = 5). Examples of interventions delivered primarily to the patient include those in which the carer had the capacity to benefit from the intervention, but in which their involvement was primarily in the capacity of interventionist (where they were trained by the music therapist to deliver the intervention to their close person) [64–66]. Other examples include those where the music therapist targeted intervention delivery to the patient, but the carer was considered to have experienced the intervention by being present in the room and able to benefit in line with the goals of care, with carer outcomes assessed [67, 68]. As would therefore be expected, the majority of interventions were delivered in the pre-bereavement phase for the carer (*n* = 31), with a minority delivered to carers across both pre- or post-bereavement stages [69, 70] or post-bereavement only [61]. Please see Table 1 for more detailed study characteristics, and Table 2 for more detailed information on intervention content.

**Table 1:** Main study summary table

**Author, Country**	**Study design**	**Carer participants**	**Patient population (supported by carer)**	**Intervention**	**Control**	**Outcome measures**	**Findings**
Baker et al. (2012) [64], Australia	Mixed-methods pilot study: Quasi-experimental (one-group, no control, pre-test and post-test design without randomisation) Qualitative (carers kept diary, interviewed post-program, thematic analysis)	Spouse carers (*n*=5) Age range: 59-81 Gender: 3 female, 2 male	Individuals with dementia living at home Early-late stage dementia	Delivered at pre-bereavement, intervention directed primarily at patient Home-based Active Music Intervention (delivered by carer to patient)	None	GDS-SF (Depression) GAI (Anxiety) MCBS (mutual communal behaviours) PACQ (positive aspects of caregiving)	Descriptive statistics only, outcome measures administered before and directly after intervention. GDS-SF: small increase in depression from pre-test (M=5.6, SD=1.82) to post-test (M= 6.2, SD=1.64). ^*^Small effect size (0.35). GAI: moderate decrease in anxiety from pre-test (M=6.0, SD=7.55) to post-test (M=3.0, SD= 2.74). ^*^Medium effect size (0.53). MCBS: small increase in mutual communal behaviours from pre-test (M= 37.6, SD=1.82) to post-test (M=38.4, SD=1.67). ^*^ Small effect size (0.46). PACQ: small increase in satisfaction from pre-test (M=41.8, SD=5.26) to post-test (M=43.0, SD=4.3). ^*^Small effect size (0.25). Qualitative: Thematic analysis indicated that intervention beneficial to relationship, satisfaction with caregiving, and wellbeing, and enhanced mood of carer and patient.
Baker & Yeates (2018) [69], Australia	Qualitative study (semi-structured individual interviews and focus group, Interpretative Phenomenological Analysis (IPA))	Family carers (*n*=4) Age not provided Gender: 2 female, 2 male	Individuals with dementia living at home and receiving care at a dementia day centre Early-late stage dementia	Delivered to carers who were pre-bereaved (*n*=3) and post-bereaved (*n*=1), directed only to carers Group Therapeutic Songwriting Intervention	N/A	N/A	Five recurring themes emerged, with all but one theme being shared by all participants: Theme 1: experience exceeded expectations, Theme 2: co-creating a song is meaningful, Theme 3: the song product is meaningful, Theme 4: learning through the songwriting experience, Theme 5: having a voice and being heard
Baker et al. (2018) [71], Australia	Mixed-methods feasibility pilot study: Quasi-experimental (controlled trial with experimental and control groups, but without randomisation, pre-test/post-test) Qualitative (focus groups and semi-structured interview, thematic analysis)	Family carers (*n*=14) Age range: 61-85 Gender: 9 female, 5 male	Individuals with dementia living at home and receiving care at a dementia day centre Stage not specified	Delivered at pre-bereavement, directed only to carers Group Therapeutic Songwriting Intervention	Standard care (*n*=6)	PHQ-9 (Depression) QCPR (Quality of the Carer Patient Relationship) PACQ (Positive Aspect of Caregiving)	Feasibility pilot study, so findings are estimates of an effect. Outcome measures administered before intervention and directly after intervention. PHQ-9: Moderate decrease was observed for the pre-post depression scores for the intervention group, medium effect size (0.64). Small increase observed for the pre-post depression scores on the PHQ-9 for control group, small effect size (0.33). QCPR: Minimal increase observed for quality of carer patient relationship in intervention group, ^*^small effect size (0.14). Moderate increase observed for quality of carer patient relationship in control group, ^*^medium effect size (d=0.57). PACQ: Small decrease observed for positive aspects of caregiving in intervention group, ^*^small effect size (0.24). Minimal decrease observed for positive aspects of caregiving for control group, ^*^small effect size (0.18). Qualitative: Theme 1. Sharing the Whole Carer Journey: From Woe to Go, Theme 2. We’re Singing the Same Tune: Finding Connections, Theme 3. Gaining Clarity Around the Carer Journey: Discovering We’ve Already Found a Way Through, Theme 4. We All Fit Under the Same Umbrella but We’re Miles Apart: Developing a Group Identity, Theme 5. We Can Stand Up for Ourselves, We’ve Got Stronger: Fostering Inner Strength And Personal Growth, Theme 6. Songwriting Groups Fill a Gap Not Met by Other Support Groups
Black et al. (2020) [67], Canada	Qualitative study (semi-structured individual interviews, audio-recorded music therapy sessions, music therapist researcher’s reflective phenomenological writing and field notes. Framework of interpretive-descriptive hermeneutic phenomenology)	Friend or family carers (*n*=8) Age range: 53-86 Gender: 6 female, 1 male, 1 unknown	Individuals with advanced cancer who have requested medical assistance in dying, setting is hospital or hospice	Delivered at pre-bereavement, and primarily to patient but with carer present Music Therapy (one-to-one sessions)	N/A	N/A	Theme 1: Immediacy of emotion (access to emotion through music): Music creates a holding space that allows for immediate access to emotion. Music connects to emotional content with immediacy. Theme 2: Reflection (on personal narratives (and pivotal life moments with their loved one, or anticipatory grief) within the music): Music invites a retrospective reflection into the carer’s experiences. A contextual reflection period is created (within the music). Theme 3: Witnessing (emotional and narrative expression): Witnessing patient’s experiences through the lens of music. Interconnectedness between patient narratives and carer narratives. Theme 4: Unexpected opportunities (for life review through music): opportunity to express unexplored/ unarticulated emotion
Brotons & Marti (2003) [72], Spain	Pilot study: Quasi-experimental (one-group, no control, pre-test and post-test design without randomisation)	Spouse carers (*n*=14) Mean age: 73.17 (SD =6.1) Gender: 9 female, 5 male	Individuals with dementia living at home Early-moderate stage dementia	Delivered at pre-bereavement, with patient only group sessions, patient-carer dyad group sessions and carer only group sessions Group Music Therapy	None	CBQ (Carer Burden) STAI (Anxiety) BDI (Depression) Satisfaction Questionnaire (developed by authors to measure satisfaction)	Outcome measures administered before intervention, directly after intervention and 2-month follow-up. CBQ: Significant decrease in carer burden between baseline, post-test and 2-month follow-up (*p*=.01). STAI: Significant decrease in carer anxiety between baseline, post-test and 2-month follow-up (*p*=.001). BDI: Significant decrease in carer depression between baseline, post-test and 2-month follow-up (*p*=.009). SQ: carers perceived improvement in social and emotional areas of their patients. All agreed intervention was positive because it helped them to relax. 66.7% added that it offered a pleasant and enjoyable space where they could share and feelings they had not been able to express before.
Choi et al. (2009) [57], South Korea	Pilot study: Quasi-experimental (controlled trial with experimental and control groups, but without randomisation)	Carers (*n*=20) Age not provided Gender not provided	Individuals with dementia attending a day care unit Moderate stage dementia	Delivered at pre-bereavement, patient-carer dyad targeted Active group music intervention	Standard care, no structured therapeutic programs (*n*=10) Offered complementary music-intervention program after study.	NPI-Q (Carer Distress)	Outcome measures administered before intervention and directly after intervention NPI-Q: Significant difference between carer total distress score between intervention and control group (*p*=.003). ^*^Large effect size (2.21).
Clark et al. (2018) [73], Australia	Qualitative study (semi-structured interviews, thematic analysis)	Family carers (*n*=12) Mean age: 75.7 (SD=10) Gender: 5 female, 4 male	Individuals with dementia living at home with family carer Early-moderate stage dementia	Delivered at pre-bereavement, patient-carer dyad targeted Group Therapeutic singing	N/A	N/A	Theme 1: Therapeutic Facilitation and Design. Intervention included supportive therapeutic features that enhanced participants’ experiences Theme 2: Accessibility. Therapeutic Singing Group Made Singing More Accessible for IWD and Their FC Theme 3: Empathic Friendship. Therapeutic group singing fostered New supportive friendships for IWD and FC Theme 4: IWD/FC Relationship. Therapeutic group singing supported Relationships between IWD and FC Theme 5: Personal Wellbeing. Therapeutic Group Singing Led to Positive Individual Experiences for IWD and FC
Clark et al. (2020) [74], Australia	Mixed-methods feasibility study: Quasi-experimental (one-group, no control, pre-test and post-test design without randomisation) Qualitative (survey and semi-structured interviews. IPA)	Family carers (*n*=14) Mean age: 67.1 (SD=10.1) Gender: 9 female, 5 male	Individuals with dementia living at home or in care home Early-late stage dementia	Delivered at pre-bereavement, patient-carer dyad targeted Group Therapeutic Songwriting	None	QCPR (Quality of the Carer–Patient Relationship) PHQ-9 (Depression) ZBI (Carer Burden)	Feasibility study, so findings are estimates of an effect. Outcome measures administered before intervention and within 2 weeks post-intervention. QCPR: No statistically significant differences for relationship quality, ^*^minimal effect size observed for increases in relationship quality (0.07). PHQ-9: No statistically significant differences for depression, small effect size observed for decreases in depression (0.20). ZBI: No statistically significant differences for carer burden, small effect sizes observed for decreases in perceived burden (0.45). Qualitative: Qualitative data indicated that session design and delivery were acceptable, and TSW was a positive shared experience with personal benefits, which supported rather than changed relationship quality.
Clark et al. (2021) [75], Australia	Qualitative study (semi-structured dyadic interviews, IPA)	Family carers (*n*=10) Mean age: 70, (SD=9.8) Gender: 6 female, 4 male	Individuals with dementia living at home or in care home Mixed dementia stages implied	Delivered at pre-bereavement, patient-carer dyad targeted Group Therapeutic Song Writing	N/A	N/A	Theme 1. Group TSW was an overwhelmingly positive shared experience, benefiting both members of the dyad and motivating further engagement with music. Theme 2. TSW was engaging and valuable as it stimulated mental processes and reignited participants’ interests, skills and memories.Theme 3. TSW provided meaningful opportunities for reflection and connection with memories and life experiences. Theme 4. TSW prompted interaction and collaboration, leading to social connections, empathic relationships and experiences of inclusion Theme 5. Group TSW included diverse challenges, however, the facilitated process supported participants to engage, highlighting abilities and challenging doubts
Dassa et al. (2020) [56], Israel	Qualitative study (case study design. Audiotaped phone counselling sessions with carer. Questions asked at end of sessions. Researcher’s log. Cresswell (2007) used for analysing case studies)	Spouse carers (*n*=2) Age range: 62-75 Gender: 2 female	Individuals with Alzheimer’s dementia Early-moderate stage dementia	Delivered at pre-bereavement, patients-carer dyad targeted Home based music therapy	N/A	N/A	Singing helped the individual with dementia to revive forgotten roles. Singing assisted the spouse in her role as primary carer. Singing improved the couple’s relationship
Denk et al. (2022) [62], USA	Mixed-methods pilot study: Quasi-experimental (one-group, no control, pre-test and post-test design without randomisation) Qualitative study (focus group discussion one week after final music therapy support group session)	Family carers (*n*=4) Mean age: 62.5 (SD=14.15) Gender: 4 female	Individuals with Parkinson’s disease or dementia Stage not specified	Delivered at pre-bereavement, directed only to carers Music therapy support group sessions	None	Three visual analog scales (perceived stress, anxiety, and depression)	Outcome measures administered immediately before and after each session. Descriptive statistics only. Stress: mean score pre-session M=4.6 (SD=2.8) decreased to post-session M= 2.0 (SD=1.5) Anxiety: mean score pre-session M= 36 (SD=1.9) decreased to post-session mean: 0.8 (SD=1.1) Depression: mean score pre-session M= 1.6 (SD=1.6) decreased to post-session M= 0.3 (SD=0.3) Qualitative: Focus group yielded 2 themes: receiving support from group members, and personal support strategies employed outside of the music therapy support group sessions
Gallagher et al. (2017) [60], USA	Quasi-experimental study (one-group, no control, pre-test and post-test design without randomisation)	Family carers (*n*=50) Age not reported Gender not reported	Individuals receiving end-of-life services in hospice (nursing home or inpatient hospice care facility) or palliative medicine (inpatient palliative medicine unit) Diagnosis not specified	Delivered at pre-bereavement, patient-carer dyad targeted Music Therapy (one-to-one sessions)	None	Music therapy family survey (developed by authors to measure benefits of music therapy for patient and carer)	Outcome measures administered post-intervention. Descriptive statistics only. Family member perceptions were positive - no family members indicated that any scale got worse. 83% of family members perceived self-improvement for quality of life, 92% for mood, 94% for stress/distress; and 84% considered the music therapy experience to be extremely helpful. 49 out of 50 family members would recommend another music therapy session for the patient, and one did not respond.
García-Valverde et al. (2020) [76], Spain	Quasi-experimental study (one-group, no control, pre-test and post-test design without randomisation)	Family carers (*n*=31) Age range: 44-87 Gender: 24 female, 7 male	Individuals with dementia using a dementia centre Stage not specified	Delivered at pre-bereavement, patient-carer dyad targeted Group songwriting program	None	SF-36v2 (perceived health status) BDI-II (Depression) STAI (Anxiety) RSS (Self-esteem)	Outcome measures administered at baseline and post-intervention. SF-36v2 – the mean score on the post-test Mental Component Summary was significantly higher compared to baseline (*p*=.02) with a medium effect size (0.57). The mean score on the post-test Mental Health dimension was also significantly higher compared to baseline (*p*=.004) with a medium effect size (0.70). There was no significant change across any other dimensions, all reporting small effect sizes (0.00-0.31). BDI-II: the mean score on the post-test BDI-II was significantly higher compared to baseline (*p*=.01), with a medium effect size (0.57). STAI: the mean score on the post-test STAI was significantly higher compared to baseline (*p*=.04), with a medium effect size (0.53). RSS: the mean score on RSS was significantly higher in the post-test measure than at baseline (*p*=.03), with a medium effect size (0.47)
Garcia-Valverde et al. (2022) [77], Spain	Qualitative study (open-question questionnaires, focus group, the last session video, and lyrics of three songs composed, inductive thematic analysis)	Family carers (*n*=31) Mean age: 63.87 (SD=13.62) Gender: 24 female, 7 male	Individuals with dementia (*n*=21), including 14 patients staying in temporary residential respite care facility Stage not specified	Delivered at pre-bereavement, carers targeted Group therapeutic songwriting program	N/A	N/A	Four themes: (1) GTSW generates a sense of connection, (2) it is a motivating, empowering, and meaningful experience, (3) it promotes emotional connection in a personal space, and (4) it increases wellbeing. Qualitative findings suggest that GTSW is a therapeutic tool that promotes carers’ connectivity, expression of emotions, and wellbeing.
Hanser et al. (2011) [65], USA	Feasibility study: Quasi-experimental (one-group time series design without randomisation)	Family carers (*n*=8) Age range: ≤65-≥85 Gender: 5 female, 3 male	Individuals with dementia, living at home Moderate-late stage dementia	Delivered at pre-bereavement, patient-carer dyad targeted Music-facilitated stress reduction program (delivered by carer to patient)	None	Visual analogue scale- relaxation, comfort and happiness CSS (Carer satisfaction)	Outcome measures administered immediately before and after each session Relaxation- statistically significant improvement for most sessions (*p*<.01 – *p*<.02). Comfort – statistically significant improvement for most sessions (*p*<.01 – *p*<.02). Happiness- statistically significant improvement for most sessions (*p*<.01 – *p*<.02). CSS- decrease but no statistically significant change over time.
Holden et al. (2019) [78], USA	Pilot study: Quasi-experimental (one-group, no control, pre-test and post-test design without randomisation)	Family carers (*n*=18) Age not reported Gender not reported	Individuals with dementia, living at home, attending memory/movement disorder clinic or neuropalliative and supportive care clinic Early-late stage dementia	Delivered at pre-bereavement, patient-carer dyad targeted Home-based Neurologic music therapy (one-to-one-sessions)	None	RSCSE (Carer self-efficacy) ZBI (Carer burden)	Outcome measures administered at baseline, directly after intervention, and at 6-week follow-up. RSCSE: non-significant increase in carer self-efficacy from baseline to 6 weeks (*p*=.39) with small effect size (0.43), and from baseline to 12 weeks (*p*=.055) with large effect size (0.92). ZBI: non-significant decrease in carer burden from baseline to 6 weeks (*p*=.50) with large effect size (1.67), and from baseline to 12 weeks (*p*=.45) with large effect size (1.62).
Kim & Dvorak (2018) [58], South Korea	Randomised Controlled Trial (cross-over design, AB-BA)	Family carers (*n*=10) Age range: 42-69 Gender: 8 female, 2 male	Individuals receiving end-of-life care in hospice Diagnosis not specified	Delivered at pre-bereavement, patient-carer dyad targeted Music Therapy (one-to-one sessions)	Chaplaincy (one-to-one sessions) (*n*=10)	FIOS (Family Intimacy)	Outcome measures administered during sessions (observation of behaviour in video-recordings). FIOS verbal intimacy scores between music therapy and chaplaincy sessions were significantly different (*p*=.74). One specific behavior of “verbally letting go of patient” was statistically significant (*p*<.015) with a large effect size (*r*=−.540), indicating higher verbal intimacy with music therapy. FIOS affective intimacy scores between music therapy and the chaplaincy were significantly different (*p*<.005) with a large effect size (*r*=−.627), indicating higher affective intimacy with music therapy. FIOS physical intimacy scores between music therapy and chaplaincy were statistically significant (*p*<.005) with a large effect size (*r*=−.627), indicating higher physical intimacy with music therapy.
Klein & Silverman (2012) [79], USA	Qualitative study (linguistic enquiry and word count -written survey)	Family carers (*n*=14) Age range: 19-75 Gender: not reported	Individuals with Alzheimer’s and other dementias, attending a family-centred care programme Stage not specified	Delivered at pre-bereavement to carers and patients Music therapy (MT) songwriting intervention	Psychoeducational intervention (PI)	N/A	Eight key themes identified, number of comments for each condition included in brackets: distraction from stress (MT: *n*=2, PI, *n*=2); reiteration of subject matter (MT: *n*=2, PI, *n*=2); fun (MT: *n*=3, PI, *n*=0); group cohesiveness and camaraderie (MT: *n*=1, PI, *n*=2); therapeutic insight and generalisation (MT: *n*=3, PI, *n*=4); appreciation (MT: *n*=2, PI, *n*=0); comment on presentation (MT: *n*=7, PI, *n*=3); and left blank (MT: *n*=2, PI, *n*=6) Linguistic Inquiry and Word Count Results Big words (>six letters) (MT: 18.50, PI: 16.89), Positive emotions (MT: 12.00, PI: 8.89), Overall cognitive words (MT: 8.50, PI: 7.56), Articles (a, an, the) (MT: 5.50, PI: 4.00), Self-references (I, me, my) MT: 4.50, PI: 4.89), Social words (MT: 4.00, PI: 7.56), Negative emotions (MT: 0.50, PI: 0.44), Total words (MT: 200, PI: 225)
Lee et al. (2022) [80], Ireland	Qualitative study (semi-structured interviews, IPA)	Family carers (*n*=4) Age range: 30-79 Gender: 3 female, 1 male	Individuals with dementia, living at home Early-stage dementia	Delivered at pre-bereavement, patient-carer dyad targeted Community-based live group singing intervention	N/A	N/A	IPA revealed four superordinate themes: (1) Social Connection; (2) Happiness and Rejuvenation; (3) Reconnection with the Self; and (4) Supporting the Carer–Cared-for Relationship.
Madsø et al. (2022) [66], Norway	Quasi-experimental study (bi-phasic AB single-case design, replicated for three sessions)	Family carers (*n*=10) Age not reported Gender not reported	Individuals with dementia, living at home Early-late stage dementia	Delivered at pre-bereavement to patients (family carers considered as collateral therapists participating in therapy to support patient, rather than being recipients of therapy themselves – but includes outcomes for carers) Active music therapy intervention	None	RSS (Carer burden)	Outcome measure administered at baseline and directly after intervention. RSS: No significant change from pre- to post-intervention (*p*=0.674)
Magill (2009a) [81], USA	Qualitative study (semi-structured interviews, field notes and researcher journal entries. Naturalistic enquiry)	Family carers (*n*=7) Age not reported Gender: 6 female, 1 male	Individuals with advanced cancer, receiving home hospice care through a hospice	Delivered at pre-bereavement, patient-carer dyad targeted Home-based music therapy program	N/A	N/A	The carers described these aspects of music in sessions to have memorable and lasting effects as follows: “music is a conduit,” “music gets inside us,” “live music makes a difference,” and “music is love.” Findings support the benefits of preloss music therapy for bereaved carers.
Magill (2009b) [82] USA	See Magill [81]	See Magill [81]	See Magill [81]	See Magill [81]	N/A	N/A	Sustaining themes pervaded the interviews throughout a range of topics and were those overall feelings and reactions that seemed to nurture and motivate the carers through the illness and beyond. The two sustaining themes were (1) joy (autonomous joy and empathic joy) and (2) empowerment. Analysis also revealed spirituality-laden themes that were sorted according to *processes of reflection*, that is, *reflection themes*. A further analysis of transcriptions revealed these themes: (1) reflection on the present: connectedness; 2) reflection on the past: remembrance; and 3) reflection on the future: hope.
Magill (2009c) [30] USA	See Magill [81]	See Magill [81]	See Magill [81]	See Magill [81]	N/A	N/A	The findings show that by including simple music therapy strategies in their sessions with patients, carers can offer their loved ones joy, comfort, relief from distress, meaning, aesthetic beauty, and peace. In this way, carers can improve their sense of empowerment.
Magill, (2011) [83], USA	Qualitative study (archived interview transcripts, content analysis)	See Magill [81]	See Magill [81]	See Magill [81]	N/A	N/A	The following 4 themes describe the significant characteristics of the role of music therapist as perceived by the carers. The music therapist displays calming and compassionate personal attributes. The music therapist uses subtleness of approach and has specialised person-centred therapeutic skills. The music therapist maintains a supportive and interactive role. The music therapist establishes and maintains an ongoing therapeutic relationship.
Mittelman & Papayannopoulou (2018) [84], USA	Pilot study: Quasi-experimental (one-group, no control, pre-test and post-test)	Friend or family carers (*n*=11) Mean age: 71.7 (SD=8.3) Gender: 6 female, 5 male	Individuals with dementia Early-moderate stage dementia	Delivered at pre-bereavement, patient-carer dyad targeted Unforgettables Choral Group	None	MOS (social support) SF-8 (health related quality of life) GDS (depression) FAM (family communication) RSS (self-esteem)	RSS: Self-esteem was the only outcome to change significantly (*p*=0.60), with an improvement in self-esteem reporting a medium effect size (0.68) SF-8: Change in health-related quality of life was not significant, with a decrease in health-related quality of life reporting a medium effect size (0.45) MOS: Change in social support was not significant, with an increase in social support reporting a medium effect size (0.42) GDS: Change in depression was not significant, with a decrease in depression reporting a minimal effect size (0.05) FAM: Change in communication with individual with dementia was not significant, with increase in communication reporting a small effect size (0.29)
O’Callaghan et al. (2013) [70], Australia	Qualitative study (semi-structured interviews, constructivist grounded theory (comparative analysis)	Friend and family carers (*n*=8) Age range: 21-≥70 Gender: 5 female, 3 male	Individuals who had died from cancer	Delivered at pre- and/or post-bereavement, patient and carers Music listening by the bereaved; song writing by the deceased and the bereaved	N/A	N/A	Theme A: remembering how music enhanced the lives of those mourned, and sharing music with deceased, was supportive. Theme B: music-elicited memories, personal empathy, and messages related to the deceased can be purposive, sometimes Unexpected, and supportive or occasionally challenging. Theme C: although music behaviors can alter, music often still improves mood, and although music use can signify mourners’ confrontation of grief, music’s occasional nonuse can signify efforts to not intensify sadness. Theme D: feeling positive when musical efforts extend the deceased relative or friend’s legacy. Theme E: what was shared and created in preloss music therapy could help before and after the death. Theme F: music recommendations for other carers to support their bereavement.
Potvin et al. (2018) [31], USA	Qualitative study (semi-structured interviews, constructive grounded theory using situational analysis)	Family carers (*n*=14) No age range provided but majority <65 Gender: 11 female, 3 male	Individuals with dementia, failure to thrive, or stroke, at end of life receiving hospice care	Delivered at pre-bereavement, patient-carer dyad targeted Hospice-based collaborative musicking	N/A	N/A	Music therapy offers a resource (e.g. stable caring relationship that carers maintain with care recipients through reconnecting with their pre-illness identity, i.e. spouse, daughter, etc.). It does this through music-making together, so that caregiving becomes more purposeful and of value and that in turn helps build carer resilience during pre-bereavement period (being able to do something of value rather than just practical tasks, e.g. washing/feeding someone). Analysis of the data resulted in the development of a theoretical model of resource-oriented music therapy with informal hospice carers during pre-bereavement. Two core categories—normalcy multilarity and collaborative musicking—and four subsequent theoretical codes—resource, risk, mediation, and outcome—explicate a theoretical avenue for conceptualising music therapy’s role in carer pre-bereavement.
Raglio et al. (2016) [85], Italy	Quasi-experimental study (one-group, no control, pre-test and post-test design without randomisation)	Family carers (*n*=4) Age not reported Gender: 3 female, 1 male	Individuals with dementia living at home Moderate-late stage dementia	Delivered at pre-bereavement, patient-carer dyad targeted Structured Active Music Therapy (one-to-one sessions)	None	ZBI (Carer Burden) HAM-A (Anxiety) BDI (Depression)	Outcome measures administered at baseline, directly after intervention, and at 1-month follow-up. Descriptive statistics only. ZBI: carer burden improved from baseline (M= 43.50), to post-intervention (M=32.75) to follow-up (M=39.75). HAM-A: anxiety improved from baseline (M=29.25) to post-intervention (M=20) to follow-up (M=19.75). BDI: depression was more stable across time from baseline (M=16) to post-intervention (M=15.25) to follow-up (16.75)
Särkämö et al. (2014) [59], Finland	Randomised controlled trial (single-blind)	Family carers (*n*=59) Age not reported Gender not reported	Individuals with dementia living at home or in long-term care Early–moderate stage dementia	Delivered at pre-bereavement, patient-carer dyad targeted Music listening intervention (group-based music coaching programme) (*n*=29)	Standard care (*n*=28), including continuation of regular group-based activities at day centre A second intervention arm, group singing sessions, was facilitated by a music teacher (*n*=27)	ZBI-12 (Carer Burden) GHQ-12 (Mental health)	Outcome measures administered at baseline, directly after intervention, and at 6-month follow-up. ZBI-12: A significant long-term specific effect was observed for the ZBI scores, Time × Group (*p*=.026), which decreased (indicating reduced burden) more in the singing group than in both music listening group (*p*=.029) and control group (*p*=.069) from baseline to Follow-up 2. GHQ: The same effect was observed also for mental health, but it failed to reach statistical significance (*p*=.174).
Tamplin et al. (2020) [63], Australia	Quasi-experimental study (controlled trial with experimental and control groups, but without randomisation, pre-test/post-test)	Family carers (*n*=44) Age not reported Gender: 30 female, 14 male	Individuals with Parkinson’s disease Early-moderate stage	Delivered at pre-bereavement, patient-carer dyad targeted ParkinSong Group Singing Programme: Weekly singing group (*n*=10) Monthly singing group (*n*=15)	Two active control groups Weekly Parkinson’s Disease dancing, painting, or tai chi classes (*n*=8) Monthly peer support groups with a similar socialisation component (*n*=11)	QCPR (Quality of Carer-Patient Relationship) Depression, Anxiety and Stress Scale DASS (Depression, anxiety, stress) EQ-5D (quality of life)	Outcome measures administered at baseline, and at 3-month, and 12-month follow-up. QCPR: Carer relationship quality total scores increased for the weekly carer singers, for the monthly singers, and weekly controls, and decreased for the monthly carer controls. No statistically significant differences between groups for perceived relationship quality as measured by the QCPR (*p*=0.09). DASS: For depression, there was a significant interaction effect of group and time (*p*=0.05). Pairwise comparisons showed a significant difference (*p*=0.031) at 12 months between carers in the weekly singing groups (whose depression scores decreased) and monthly control groups (whose depression scores increased). There were no statistically significant differences between groups for carer anxiety, but clinically relevant trends towards a group main effect (*p*=0.067) for carer stress. Carers in the weekly singing groups had significantly lower stress scores than that of weekly controls (*p*=0.044) and of monthly controls (*p*=0.022) at 12 months. EQ-5D: No statistically significant differences between groups for carer quality of life (*p*=0.70)
Tamplin et al. (2018) [86], Australia	Mixed-methods feasibility study: Quasi-experimental (one-group, no control, pre-test and post-test design without randomisation) Qualitative (semi-structured interviews, thematic analysis)	Family carers (*n*=12) Age range: 58-88 Gender: 6 female, 6 male	Individuals with dementia, living at home Early-moderate stage dementia	Delivered at pre-bereavement, patient-carer dyad targeted Remini-Sing Music Therapy Intervention (group singing intervention)	None	QCPR (Quality of Carer Patient Relationship) PHQ-9 (Depression) SWLS (Satisfaction with life) FS (Flourishing) PACQ (Positive aspects of caregiving)	Feasibility study, so findings are estimates of an effect. Outcome measures administered before intervention, midway through (11 weeks) and post-intervention. QCPR: No statistically significant pre to post differences between time-points for relationship quality. A medium effect size for mid-point increase (0.65) but this decreased again at post-intervention. PHQ-9: No statistically significant pre to post differences between time-points for depression. Depression scores were low at baseline and remained relatively stable. SWLS: No statistically significant pre to post differences between time-points for satisfaction with life. Satisfaction with life scores increased over time (medium effect at mid-point, 0.51, and small effect at post-point, 0.41). FS: No statistically significant pre to post differences between time-points for flourishing. Flourishing scores were high at baseline and also remained relatively stable. PACQ: No statistically significant pre to post differences between time-points for positive aspects of caregiving. Positive aspects of caregiving scores were lower post-intervention (medium effect, 0.56).
Teut et al. (2014) [68], Germany	Qualitative study (open interviews, grounded theory)	Family carers (*n*=3) Age not reported Gender: 1 female, 2 male	Individuals with advanced cancer and at end of life, receiving palliative care in a stationary hospice	Delivered at pre-bereavement, primarily delivered to patient, (body tambura placed on or near patients’ body, but family carers who were present were considered to have “experienced” the intervention) The Body Tambura	N/A	N/A	The most often described subjective experiences (by carers) were the generation of relaxing images and visualisations. Family members enjoyed listening to the music and felt more connected with the sick family member.
Thompson et al. (2022) [87], Australia	Qualitative study (semi structured interviews, IPA)	Family carers (*n*=7) Age range: 50-89 Gender: 5 female, 2 male	Individuals with dementia living at home Early-moderate stage dementia	See Tamplin et al. [86]	N/A	N/A	Eight themes identified the perceived benefits of choir singing and elements of the choirs and research project more broadly that influenced participation. Overarching theme 1: participating in the Remini-Sing choirs afforded experiences of personal and social benefits: Theme 1: therapeutic choir participation fosters positive feelings Theme 2: singing in THE choir promotes cognitive stimulation, connection to identity, for individuals with Dementia Theme 3: opportunities to engage musical identity Theme 4: choir participation enables much needed social connections Theme 5: participating as dyads for pragmatic and personal reasons Overarching theme 2: pragmatic aspects of the choirs and research design impacted the experience for participants: Theme 6: getting started can be challenging – a welcoming environment is needed Theme 7: accessibility of therapeutic Design Theme 8: sustainability of choirs is desired
Young & Pringle (2018) [61], Canada	Qualitative study (individual semi-structured qualitative interviews. Optional written participant feedback after each session. Researcher reflexive journal and analytic memos. IPA)	Family carers (*n*=7) Age range: 51-80 Gender: 7 female	Individuals who were deceased within past three years, with none of these deaths sudden or unexpected Deaths due to cancer (*n*=3), other diagnoses not specified (*n*=4)	Delivered at post-bereavement, carers only Singing Well group (community hospice-based music therapy group)	N/A	N/A	Themes organised in five categories. 1: Group singing experiences/the Singing Well context: Fostered feelings of connection, evoked emotions, allowed participants to enjoy themselves in safe space and express themselves in a way that was different than talking. Importance of qualities of music therapist. 2: Vocal warmups, breathing and relaxation exercises: mixed responses to these. 3: Songs (precomposed/original): Validated and re-conceptualised grief and loss, evoked memories, emotional release. Choosing songs was important. Songwriting. 4: Improvised vocal experiences: For most, felt supportive, evocative, and liberating. Provided musical framework where participants could feel togetherness and be independent. 5: Overall experiences of Singing Well: Commitment to the group. Motivated participants to make changes, explore new/revive previous interests and helped to move forward in their lives. Experience of the group and impact was difficult to describe.

* Statistically significant at >0.05 level

**Table 2:** Intervention content table

**Study**	**Intervention Theory**	**Intervention Content**	**Intervention Delivery Schedule**	**Interventionist**	**Treatment Fidelity**	**Setting**	**Unit of delivery**
Baker et al. (2012) [64], Australia	Not reported	Delivered at pre-bereavement, directed primarily to patient Home-based Active Music Intervention Background information collected Carer instructed to use music strategically to facilitate interaction with spouse (flexibly to status on that day, e.g. activity order). Carers then engaged spouse in music-sharing Music selected by couple. Live singing familiar songs (unaccompanied or with audio player), gentle movement to music, listening to relaxing music. After each activity, carer asked spouse to recall memories (with prompts), and kept diary after each session	Initial instruction session x 1 Music-sharing, 3 x 20–30-minute sessions per week for 6 weeks	Music therapist provided initial instruction session for carer to use music strategically with spouse Spousal carers then delivered intervention	Not reported	Instruction and all other sessions provided at home	Initial session delivered to patient-carer dyad, later sessions delivered by individual carer to patient
Baker & Yeates (2018) [69], Australia	Not reported	Delivered to carers who were pre-bereaved or post-bereaved, directed only to carers Therapeutic songwriting group programme Group discussion, and collaborative composition (musical accompaniment and lyrics) of song with three verses, a chorus, and a bridge Participants rehearsed the material Whiteboard, keyboard	4 x 60-minute sessions over unspecified period of time	2 music therapists	Not reported	Dementia day centre	Group (*n*=4 members)
Baker et al. (2018) [71], Australia	Insight-orientated, strengths-orientated, narrative, and cognitive reframing therapeutic songwriting frameworks	Delivered at pre-bereavement, directed only to carers Group Therapeutic Songwriting Intervention Participants shared stories and brainstormed to create lyrics and music for one song, then refined, rehearsed, and recorded Music therapist predominantly responsible for music creation, but participants encouraged to be involved at numerous stages of the process. (musical preferences/style, and musical choices) Live song writing, co-created with therapist	6 x 60-minute sessions, weekly	One trained music therapist	Implied manualised protocol based off previous pilot study of 3-week intervention No further information reported	Private rooms at 2 not for profit dementia day care centres	Group (*n*=3-5 members)
Black et al. (2020) [67], Canada	Not reported	Delivered at pre-bereavement, primarily to patient but carer present Music Therapy Receptive (inter-active) listening, song writing, psychotherapeutic processing through lyric analysis and active playing/singing Tailored to patient goals, reached through discussion with music therapist Goals included reminiscence, comfort (familiarity, and ease and calm while listening to live music), symptom management, and song writing/legacy (patient-created/therapist-supported songs as therapeutic outlet for self-expression and narrative creation, often gifted to family member after death)	1-5 music therapy sessions (session length not specified), provided from 2 months prior to death to day of death	1 music therapist/ researcher	Music therapist/ researcher delivered No further information reported	Multi-site between several hospitals and a residential hospice	Individual patients, sometimes with carer
Brotons & Marti (2003) [72], Spain	Not reported	Delivered at pre-bereavement, with patient only group sessions, patient-carer dyad group sessions and carer only group sessions Group Music Therapy Therapists administered music experiences/preferences questionnaire with patients and carers and conducted music therapy session to observe. Participants divided in 2 groups with equal numbers of carers and patients in each Patient-carer dyad sessions (instrumental ensembles, and sing-alongs); carer only sessions (singing, music listening, music relaxation exercises, musical games, and song-writing)	Participants stayed on location for 12 days Patient and carer dyads = 7 morning sessions; carer only = 4 afternoon sessions (session length not specified)	2 music therapists	Not reported	Participants stayed at rural house, equipped to meet their physical needs	Group (patient-carer dyad sessions *n*=28 members; carer only sessions *n*=14 members)
Choi et al. (2009) [57], South Korea	Not reported	Delivered at pre-bereavement, patient-carer dyad targeted Active group music intervention (*n*=10) Singing songs, analysis of libretto, making musical instruments, playing instruments, e.g. pianos and hand bells, song drawing, song writing, listening to various kinds of songs as concentration processes	15 x 50-minute sessions, 3 times a week for 5 weeks	3 certified professional music therapists	Certified music therapists delivered No further information reported	Special dementia-care unit	Group (*n*=10 members), with patient-carer dyads
Clark et al. (2018) [73], Australia	Not reported	Delivered at pre-bereavement, patient-carer dyad targeted Group Therapeutic singing Vocal warmups and exercises, singing familiar participant-requested songs (selected by therapist), learning new songs and singing skills introduced by the researchers, and socialisation over afternoon tea Participants encouraged to contribute to running of group Power-point presentation with ∼75 participant nominated songs and accompanying lyrics supported singing Live music provided by therapists (guitars, keyboard, and banjo) with support for carers to sing and, occasionally, percussion instruments	20 x ~120 minute sessions on differing dates over 12-month period	Usually 2 registered music therapists, sometimes 1	Registered music therapists delivered No further information reported	Spacious room at large public health facility	Group (n≤15 patient-carer dyads)
Clark et al. (2020) [74], Australia	Not reported	Delivered at pre-bereavement, patient-carer dyad targeted Group Therapeutic Songwriting Brainstorming, reminiscence, lyric creation, music listening, familiar song singing, instrument playing. Encouraged to listen to preferred music between sessions for inspiration Groups wrote and recorded 2–3 songs per group, using song parody and song collage and setting original spoken word poetry to background music. Tailored to meet participants’ needs Whiteboard and markers, visual cues (e.g. photographs), devices for streaming commercial music, guitar, percussion instruments, lyric song books, and recording equipment	6 x 60-minute sessions, weekly (held over 3.5-month period with 1- to 2-week break)	Each group facilitated by qualified music therapist with an accredited degree and evidence of ongoing professional development	Study design registered Session facilitation protocol Music therapists experienced and received training Study was a feasibility study, included interventionist reflection on treatment and participant engagement	Community aged care support facilities or residential aged care homes Music-listening at home between sessions was encouraged	Group (*n*=2-3 patient-carer dyads) 4 homogeneous groups based on relationship type (spousal or family) and living situations (IWD in community or care home)
Clark et al. (2021) [75], Australia	Not reported	Delivered at pre-bereavement, patient-carer dyad targeted Group Therapeutic Song Writing Music therapy assessment before program Brainstorming (sharing experiences to find group focus); lyrics (review ideas, find main message and structure); music (explore group preferences and discuss ideas and influences) Each group had 5 sessions and created and recorded 2-3 songs using song parody song collage/original lyrics Whiteboard and markers, song books, lyric sheets, guitar, percussion instruments, speakers, recording equipment and software	6 x 60-minute sessions, weekly	2 experienced music therapists	Experienced music therapist who conceptualised the study provided weekly supervision to both music therapists who facilitated TSW groups	Private rooms at community spaces accessed through partnering organisation or residential aged care facility where participants with dementia were living	Group (*n*=2-4 patient-carer dyads) 4 homogeneous groups based on relationship type (spousal or family) and living situations (IWD in community or care home)
Dassa et al. (2020) [56], Israel	Not reported	Delivered at pre-bereavement, patient-carer dyad targeted Home based music therapy Background information session Adapted to couples’ preferences and needs that day. Singing (with therapist guitar accompaniment), music listening, dance, playing percussion instruments Participants selected music; favourite songs used to elicit reactions and encourage participation. Folk, traditional, religious, and children’s songs/lullabies Small instruments, smart phone, amplifier, YouTube Music therapist phone counselling sessions with primary carer	12 x 60-minute sessions of music therapy, weekly 6 x 30-minute telephone counselling sessions, fortnightly	3 qualified music therapists with expertise in area	Experienced music therapists delivered No further information reported	Homes of the individuals with dementia	Individual patient-carer dyad
Denk et al. (2022) [62], USA	Not reported	Delivered at pre-bereavement, directed only to carers Music therapy support group sessions 7 activities: 1. 15-minute verbal check-in: welcome and sharing updates/challenges. 2. 5-minute movement to music exercises: recorded music played, researcher demonstrated movements. 3. 10-minute guided breathing experience: relaxation and preparation. Researcher provided rhythmic, musical autoharp cues. 4. 5-minute music listening activity: researcher played guitar and sang song related to session theme. Participants given printed lyrics and encouraged to sing. 5. 10-minute music-based activity to connect song content to session theme: researcher-led songwriting or song discussion, allowed participant self-expression and mutual support. 6. 10-minute caregiving discussion experience: opportunity for emotional expression and building relationships during shared experiences. 7. 5-minute verbal check-in: session closure, foster comfortable environment for mutual support.	6 x 60-minute sessions, bi-weekly	Researcher, a board-certified music therapist with nine years of experience providing clinical services for populations including elderly adults with dementia, Alzheimer’s disease, Parkinson’s disease, and long-term carers	Researcher created program manual containing detailed explanations and session plans	Large, open area within an office building	Group (*n*=4 members)
Gallagher et al. (2017) [60], USA	Not reported	Delivered at pre-bereavement, patients and carers targeted Unnamed – “Music therapy sessions” Patient-preferred music, music listening, singing, choosing songs, playing instruments, music-assisted relaxation techniques, analysing song lyrics, and writing songs Music mostly chosen by patient, music therapist, and family. Sometimes by patient alone, music therapist alone, or family alone	30-90 minute sessions (average 51 minutes) 43% of sessions were initial sessions and 41% were follow-up (16% missing data)	Board-certified music therapist (MT-BC)	Board-certified music therapist delivered No further information reported	Hospice (nursing home or inpatient hospice care facility) or palliative medicine (inpatient, 23-bed palliative medicine unit)	Individual patient alone or with carer. Carers had an active input and were present during sessions
García-Valverde et al. (2020) [76], Spain	Not reported	Delivered at pre-bereavement, directed only to carers Group songwriting program Developed group climate, trust, and cohesion. Promoted connection and expression through therapeutic singing and musical collage, and explored potential song themes on canvas. Collaboratively created lyrics, then music), and recorded the song. Groups came together for joint closing to share songs and experiences	12 x 60-minute sessions, over 3 months	Qualified music therapist	Intervention protocol Model and sessions were designed and facilitated by researcher music therapist All groups received same intervention protocol	National Reference Centre for Alzheimer’s disease and Dementia Care of Salamanca (acronym in Spanish, CREA) - music therapy room	3 groups (*n*=6-9 members)
Garcia-Valverde et al. (2022) [77], Spain	Not reported	Delivered at pre-bereavement, directed only to carers Group therapeutic songwriting program (GTSW) In four phases: 1. developed group climate; 2. promoted connection and expression through therapeutic singing and musical collage, and explored potential song themes on canvas; 3. collaboratively created lyrics, then music; 4. recorded the song and groups came together for joint closing to share songs and experiences	12 x 60-minute sessions, over 3 months	Qualified music therapist	Manualised protocol implied. Intervention process developed in earlier study [76] Interventions delivered by first author All groups followed same intervention protocol	The music therapy room of the CREA (see above [76])	3 groups (*n*=6-9 members)
Hanser et al. (2011) [65], USA	Not reported	Delivered at pre-bereavement, directed primarily to patient Music-facilitated stress reduction program (delivered by carer to patient) Training session: music therapist met dyad to discuss musical selections (from list of recordings), and rehearse how IWD could be engaged with music Individualised CDs with recorded instructions. Families listened to CD together and chose from following: Listen to carer/IWD’s choice of music and discuss e.g. memories; gentle exercise and movement to music; music-listening and relaxation/ visualisation; music-listening and artistic expression/discussion; singing or rhythmic accompaniment/ improvisation; music listening for specific needs, e.g., to facilitate sleeping etc.	Initial 120-minute training session Families were asked to listen to individualised CD together, 3 days each week. Family members selected days/times. Target goal of 8-20 total sessions	Music therapist trains family carer in strategies conducted at home by the carer alone	Protocol Researcher telephoned carers once a week to ensure that they were implementing protocol, completing the visual analogue scales, and sending data to investigator	At home of individual with dementia	Training session delivered to individual patient-carer dyad Program delivered by individual carer to patient
Holden et al. (2019) [78], USA	Not reported	Delivered at pre-bereavement, patient-carer dyad targeted Home-based Neurologic music therapy (NMT) Carer attended all sessions to learn NMT techniques and improve interactions with IWD Sing/play-along, improvisation, rain stick, fill-in-the blank singing tasks, color-coded bells, reminiscence. Tasks gradually increased in complexity. IWD choice of 3 recordings on weekly theme, e.g. “Travel”. Visual/tactile aids and dyad reminiscence discussion prompts. Closed with goodbye song and therapist provided carer instruction until next in-person session Protocol modified for stage of dementia Guitar, small percussion instruments, pentatonic xylophone, writing materials, song list, visual and tactile aids, theme-based music playlists on a tablet, and carer education sheets	6 x (first session 90 minutes, remaining sessions 60 minutes), weekly for 6 weeks	4 board-certified music therapists (MT-BC) with NMT training, trained by MT-BC who designed protocol	Board-certified music therapist MT-BC (R.S.), with NMT training designed protocol. Protocol reviewed and implemented by 4 MT-BCs with NMT training Interventionists trained by the board-certified music therapist MT-BC (R.S.) to ensure standardised and consistent protocol implementation	At home of individual with dementia	Individual patient-carer dyad
Kim & Dvorak (2018) [58], South Korea	Not reported	Delivered at pre-bereavement, patient-carer dyad targeted Unnamed – music therapy session (one-to-one sessions) Music-assisted relaxation (therapist live guitar improvisation, with breathing exercises, for energy and relaxation) Music listening (therapist live guitar music – song selection according to patient preferences) Music-based life review/reminiscence (therapist live guitar music familiar to the patient as a stimulus to evoke, share, and validate memories and feelings)	1 x minimum 15-minute session	Delivered by board-certified music therapist with 1 year of work experience in hospice, employed at hospice agency in hospice general inpatient care units within a larger hospice	Principal investigator determined minimum session length based on typical therapy services at the hospice Board-certified music therapist delivered Interventionists blind to study purpose and provided treatment as usual in hospice setting	Hospice general inpatient care units within a larger hospice Patient bedside where carers were present with their dying loved one	Individual patient-carer dyad
Klein & Silverman (2012) [79], USA	Not reported	Delivered at pre-bereavement to carers and patient (patients included in psychoeducational intervention but unclear if also included in music therapy intervention) Music therapy songwriting intervention Group sang familiar song, “With Love From Me to You” (Lennon & McCartney, 1963, track 2) as basis of new composition about coping skills - title, “With Love From Me to Me”. Interventionist asked for participant input for lyric replacement with guitar accompaniment. Guiding questions used to help compose new lyrics. Lyrics typed on laptop and projected onto large screen using LCD projector. At session conclusion, song played in entirety After session, lyrics e-mailed to members	1 x 45-minute session	Music therapist (4th-year music therapy student at the same university as the care program)	Music therapist delivered No further information reported	Not reported	Group (*n*=7 members)
Lee et al. (2022) [80], Ireland	Not reported	Delivered at pre-bereavement, patient-carer dyad targeted Community-based live group singing intervention Each session followed flexible structure, driven by participants Session began and ended with vocal warm-ups; goodbye song “Hit the Road Jack” with participants’ names. Aimed to promote social connection (e.g. social warm-ups/games), expression and creativity in-the-moment (e.g. improvisation; song-writing), reminiscence (e.g. familiar songs; discussion), and life-long learning/cognitive stimulation (e.g. addition of harmony lines; unfamiliar songs Tea, coffee, and biscuits provided before and after each session	6 x 60-minute sessions, weekly	Qualified music therapist with a background in Irish traditional music	Qualified music therapist delivered No further information reported	Private room in community arts centre	Group (*n*=6 patient-carer dyads)
Madsø et al. (2022) [66], Norway	Main principles of resource-oriented music therapy	Delivered at pre-bereavement, intervention delivered primarily to patients (carers considered as participating to support patient, rather than being recipients) Active music therapy intervention Musical history of IWD and shared musical history of dyad mapped. Treatment plan including personal goals made in collaboration with dyad. Music therapist came to participant’s home for first weekly session with dyad, and guided dyads in choosing musical activities for second weekly session, initiated by carer (collateral therapist) Activities with therapist or carer: - Singing together (often with music therapist playing guitar, piano, or accordion) - Playing instrumental music together (e.g. drumming, guitar or harmonica) - Improvising music together - Listening to live or recorded music - Moving to music alone or together - Relaxation exercises to music - Activities often followed by conversations about music and/or memories coming to mind	Aimed for 2 x ~ 45-minute sessions (tailored to needs of IWD) weekly, over 10 weeks	Music therapist or carer (collateral therapist)	Followed principles originating from the manual of Rolvsjord et al. (2005) Logged number of sessions received (and who delivered), proportion of sessions dedicated to musical elements, adverse effects, and how participants rated collateral sessions	At home of individual with dementia	First weekly session delivered to individual patient-carer dyad. Second weekly session delivered by individual carer to patient
Magill (2009a) [81], USA	Not reported	Delivered at pre-bereavement, patient-carer dyad targeted Home-based music therapy program Common strategies are as follows: 1. Use of precomposed songs: 2. Lyric improvisation and song composition: 3. Imagery in music: carers may select images that instill peace, security, comfort, and pleasure. Words are accompanied by chants or improvised melodies adapted to meet moods and needs. 4. Music listening. Carers assist loved ones in singing, selecting songs, and participating in the lyrical and musical interactions Strategies to address moment-to-moment patient and carer needs, wishes, and emergent issues	Not reported	Music therapist	Not reported	At home of patient	Individual patient and if relevant, carer (as part of family unit of care, carers invited and encouraged to be present in sessions as often as appropriate)
Magill (2009b) [82], USA	Not reported	See Magill [81]	Not reported	See Magill [81]	Not reported	See Magill [81]	See Magill [81]
Magill (2009c) [30], USA	Not reported	See Magill [81]	Not reported	See Magill [81]	Not reported	See Magill [81]	See Magill [81]
Magill (2011) [83], USA	Not reported	See Magill [81]	Not reported	See Magill [81]	Not reported	See Magill [81]	See Magill [81]
Mittelman & Papayannopoulou (2018) [84], USA	Not reported	Delivered at pre-bereavement, patient-carer dyad targeted Unforgettables Choral Group Practice of melody, rhythm, form, and dynamics. Participants learn nuance of performance, techniques of breathing and vocalisation, and following the leadership of the conductor. Conductors encourage everyone to join in. Both carer and IWD are encouraged to be creative Conductors consult with participants about songs they want to sing. Participants sing songs from their youth and learn new ones Provide enjoyable experiences and give IWD and carers an opportunity to share, create, express, learn, and enjoy time together. 15-minute break with refreshments and opportunity for social interactions and support Rehearsal ends with a song that involves movement/dance	13 x 120-minute rehearsals, weekly for 13 weeks before each concert They rehearse 18 songs for each concert	Choral conductor and assistant conductor (took turns as piano accompanist), music therapist, and research and support team directed by first author	Music therapist assisted conductors in assuring appropriateness of materials, techniques, and rehearsal strategies. First author directed support team No further information reported	Church setting	Chorus group (size not specified)
O’Callaghan et al. (2013) [70], Australia	Not reported	Delivered at pre- and/or post-bereavement, directed to patients and carers Music listening by the bereaved. Song writing by the deceased and the bereaved Carers’ deceased family members had written songs in music therapy which were recorded on CDs, offering continued connection after death. Recordings of songs were played or sung live by carers at family members; funerals Four participants had shared music therapy sessions with the deceased	Not reported	1 music therapist/researcher (previously had provided music therapy to 2 participants who had shared music therapy sessions with the deceased)	Researcher/music therapist delivered No further information reported	Not reported, but assumed to be the hospital or hospice where the music therapist/ researcher was previously employed	Delivered to individual patients, prior to their death (4 carers had shared these sessions; 2 carers had not personally experienced music therapy but knew that the deceased person had received music therapy)
Potvin et al. (2018) [31], USA	Not reported	Delivered at pre-bereavement, patient-carer dyad targeted Hospice-based collaborative musicking Co-constructed recreative musicking (familiar, precomposed music re-created through any combination of voice, guitar, piano, and percussion), communal music therapy (engagement of other entities, such as individual residents or the general milieu in a care recipient’s long-term care facility), and legacy projects (musical artifacts, e.g., video recordings of sessions, etc.) Carer roles: director/conductor, primary vocal, secondary vocal, or musical presence Music tailored based on patient assessment and adjusted to carer patient preferences and engagement	Not reported	Board-certified music therapist	Board-certified music therapist delivered No further information reported	Hospice, homes and long-term care facilities	Individual patient-carer dyad, some communal activities in long-term care facility (group size not specified)
Raglio et al. (2016) [85], Italy	Not reported	Delivered at pre-bereavement, patient-carer dyad targeted Structured Active Music Therapy (AMT) Strategies, and mode of delivery, etc. not reported apart from reference to “sonorous-music”	12 x 40-minute sessions, twice weekly	Trained music therapist	Trained music therapist delivered No further information reported	Not reported	Individual patient-carer dyad targeted
Särkämö et al. (2014) [59], Finland	Not reported	Delivered at pre-bereavement, patient-carer dyad targeted Music listening intervention (group-based music coaching programme) Listening to CD songs and discussing emotions, thoughts, and memories that they evoked. Visual cues (e.g., album covers) used to stimulate reminiscence Traditional folk and popular songs selected based on IWD preferences. Session themes (e.g., childhood) or how to use music in everyday life (e.g., for reminiscence). Homework: music listening, with aim of rooting music activity to everyday home setting Final session - participants given compiled CDs of their favorite songs and encouraged to continue musical activities at home on regular basis	10 x 90-minute sessions, weekly at each centre	Music therapist	Session participation, homework completion, and continuation of activities after intervention monitored (appears to be retrospective) No further information reported	5 day activity centres and inpatient centres	Group (*n*=5 patient-carer dyads)
Tamplin et al. (2020) [63], Australia	Not reported	Delivered at pre-bereavement, patient-carer dyad targeted ParkinSong Group Singing Programme Vocal warm-ups, exercises and activities designed to develop and extend respiratory strength and control, vocal loudness, articulation, pitch control, and communication confidence, and address sensory processing deficits. Communication strategies taught and practised to increase vocal loudness and to improve self monitoring of communication and internal cueing. Built on during singing activities using familiar songs and rounds, with focus on respiratory support and high intensity vocal effort Socialisation and refreshments at end of each session (informal opportunities to practise communication strategies in safe and supportive peer environment Songs selected according to participant preference and in accordance with ParkinSong guidelines	Weekly sessions compared with monthly: Weekly: 120-minute sessions, weekly over 12 months Monthly: 120-minute sessions, monthly over 12 months Total sessions not specified	Weekly: Music therapist, speech pathologist and allied health assistant Monthly: Community musicians and volunteers with training and support from music therapists and speech pathologists All facilitators received training in ParkinSong protocol	ParkinSong protocol Facilitators received training in protocol with regular fidelity checking from research team	Not reported. Only notes “safe and supportive peer environment”	Group (size not specified), with patient-carer dyads
Tamplin et al. (2018) [86], Australia	Not reported	Delivered at pre-bereavement, patient-carer dyad targeted Remini-Sing Music Therapy Intervention (group singing) Singing-based activities to enhance memory, communication, wellbeing, and group cohesion. Vocal warm ups (e.g. breathing, vocalising, physical exercises), singing familiar, participant-requested songs, learning new songs, harmony parts, rounds, and singing skills, and social interaction and peer support over afternoon tea Songs acapella or accompanied with live music on keyboard, guitar, and or banjo by music therapists. Song keys adjusted as needed to match vocal range of group. Variation of musical elements introduced as groups became more comfortable singing together (e.g., dynamic variation, simple harmonies, movement to music) Name tags, appropriate seating, accessible facilities, large screen projection of lyrics	20 x 120-minute sessions, weekly	2 trained music therapists	Trained music therapists delivered No further information reported	Patients and active carers targeted Sessions were held in a spacious room at a large public health facility	Group (*n*=9-12 patient-carer dyads)
Teut et al. (2014) [68], Germany	Not reported	Delivered at pre-bereavement, primarily delivered to patient, (body tambura placed on or near patients’ body, but carers who were present were considered to have “experienced” the intervention) The Body Tambura Body Tambura either placed directly onto the body of the patient while lying down or played a short distance away from the patient. Each session began with greeting, short introduction and a request for feedback on the previous session. At the end of each session, the patients had the opportunity to give feedback and share their experiences	Up to 5 x 5-30-minute sessions (determined by patient requirements), weekly	2 experienced music therapists, both having more than 10 years of professional experience	Experienced music therapists delivered No further information reported	Stationary 16-bed hospice	Individual patients and carers (body tambura placed on or near patients’ body, but family carers who were present for the treatment were considered to have “experienced” the intervention)
Thompson et al. (2022) [87], Australia	See Tamplin et al. [86]	See Tamplin et al. [86]	See Tamplin et al. [86]	See Tamplin et al. [86]	See Tamplin et al. [86]	See Tamplin et al. [86]	See Tamplin et al. [86]
Young and Pringle (2018) [61], Canada	Not reported	Delivered at post-bereavement, directed only to carers Singing Well group - community hospice-based postloss bereavement music therapy group Various creative vocal expression, e.g., breathing and relaxation, vocal warm ups, humming, toning, chanting, vocal improvisation, song writing/song sharing, group singing (participants’ song choices), and a closing song Structure adapted to participants’ needs 2 Songbooks (lyrics only). When a participant requested a song not contained in the book, facilitators improvised, participant lead singing, group sang along with online recording, or facilitators learnt song and offered in a subsequent session	6 x 90-minute groups, over 3-month period	2 certified music therapists (MTA) with a combined total of 42 years of experience	Pilot sessions informed development of protocol Experienced music therapists delivered	Community hospice	Group (*n*=7 members)

A slightly higher number of interventions were group-based (*n* = 18) with a smaller number delivered to individuals or patient-carer dyads (*n* = 16). All interventions delivered to the carer only (*n* = 6) were group-based, and all interventions directed primarily to the patient in the presence of the carer, (*n* = 5) were delivered to individuals or patient-carer dyads.

Interventions were delivered across a range of settings, including the home of the individuals with dementia (*n* = 9), residential or day care specialist facilities (*n* = 7), public health or other community facilities (*n* = 7), hospices or hospitals (*n* = 6). Two interventions were delivered across combinations of these settings. Setting was not reported in three studies.

All interventions involved trained and credentialed music therapists, who were also responsible for directly delivering the intervention in the vast majority of studies (*n* = 32). In three studies, the carer was trained by the music therapist to deliver the intervention; in two of these studies the carer then delivered the intervention alone. In two interventions, music therapists were joined by additional interventionists such as choral conductors, researchers, speech pathologist, allied health assistant, community musicians, and volunteers. Reporting on interventions often lacked clarity and detail. This was particularly the case for areas such as treatment fidelity and intervention theory.

The number of intervention sessions ranged from one to 20. Five interventions were delivered across one to five sessions, eight were delivered across six to ten sessions, and twelve were delivered across 11 to 20 sessions. One additional study reported eight to 20 sessions, and one group intervention comparing monthly to weekly sessions did not specify the total number of sessions for each group, although the weekly group likely received a greater number of sessions than 20 [63].

Studies often failed to report detail on intervention delivery schedules or duration, with seven studies reporting no information, and three more reporting limited information (for example, reported the delivery schedule, but not the session duration).

Some studies provided extensive detail on music therapy activities and their therapeutic justification (e.g., Madsø et al. [66]),while others provided extremely limited information on what “music therapy” referred to (e.g., Raglio et al. [85]).

### Methodological quality of included studies

Studies that scored 50% or less were rated as being of lower methodological quality. Those scoring between 50–75% were rated as medium quality, and those scoring above 75% were scored as higher quality. Qualitative studies (*n* = 17) scored higher for methodological quality compared to the included quasi-experimental, mixed-method and RCTs, ranging from 50–100%, with nine out of 17 (53%) scoring 100% across all 10 quality criteria questions (Additional file 3). Reflexivity and researcher positionality were the weakest areas, with eight out of the 17 studies (59%) failing to locate the researcher culturally or theoretically, and four out of the 17 (76%) failing to address the influence of the researcher on the research, and vice-versa. Mixed-method studies that included a qualitative design (*n* = 7) were not conducted to the same level of quality, ranging from 20 to 80% overall, with the exception of Tamplin et al.’s [86] study (reported in full in Clark et al. [86]) which was rated as higher quality overall. Again, methodological limitations were found for reflexivity and researcher positionality, with none of the mixed-method studies (excluding Tamplin et al. [86]) reporting on these quality criteria (Additional file 3).

For quasi-experimental studies with no control group, a yes response was reported for question 2 as per JBI guidance. Purely quasi-experimental studies (*n* = 8) were mostly rated as lower to medium quality, with only two including a control group and rated as higher quality. Lack of appropriate statistical analyses was a particular issue, with the majority of studies demonstrating no evidence of power analysis being performed and with small sample sizes, likely to be underpowered. Several studies identified as feasibility/pilot studies, did not discuss justification of sample size to estimate a parameter. Mixed-method studies (*n* = 7) that included a quasi-experimental design also scored medium to lower methodological quality, ranging from 43 to 66% across all studies. Only one study included a control group. Follow up was complete in 86% of studies, outcomes were measured in a reliable way in 43%, but only one study used appropriate statistical analyses, with the same limitations as found for purely quasi-experimental studies (Additional file 4).

There were only two RCTs, and both were rated as medium quality. Methodological imitations for the lower scored study (54%) included lack of clarity around concealment of allocation to treatment groups, blinding of participants and outcome assessors to treatment assignment (Additional file 5). The slightly higher scored study (61%) had similar treatment groups at baseline and blinded outcome assessors to treatment assignment. Reliability of outcome measures was unclear for both studies, and both lacked appropriate statistical analyses which included no evidence of power analysis being performed and with small sample sizes, were likely to be underpowered.

## Findings of the review

### Results of the meta-synthesis of quantitative research findings

There were 17 studies which were either purely quantitative or which included a quantitative component. Quasi-experimental studies (*n* = 15) were largely of pre-post design, and only three included a control group. Sample size was small overall, with a median of “14” and range of between “4” and “50” participants. The two RCTs [58, 59] also included a small sample size, 10 and 89 participants respectively. The outcome data is summarised below, aligned to core outcomes for evaluating bereavement interventions in palliative care [6] and previously identified risk factors for complicated grief [14]. No studies reported either a cost-unit analysis or cost-effectiveness outcomes.

### Ability to cope with grief

#### Accessing appropriate support (PGD risk factor- poor perceived social support)

##### Outcomes

Only one study aligned to the core outcome domain “ability to cope with grief”, and specifically the “accessing appropriate support” dimension. Mittelman and Papayannopoulou [84] assessed functional social support by administering the Medical Outcomes Study Social Support Survey (MOS Social Support Survey, [88]).

##### Findings

Mittelman and Papayannopoulou [84] with 11 family carers of individuals with dementia noted a non-significant increase from pre- to one-week post music therapy intervention in social support, with a medium effect size.

#### Quality of life and mental wellbeing

##### Outcomes

Five studies [60, 63, 76, 84, 86] assessed quality of life as a broad construct. Garcia-Valverde et al. [76] and Mittelman and Papayannopoulou [84] used two different versions of the Short-Form Health Survey, the SF-36V2 [89] and the SF-8 [90]. Tamplin et al. [63] administered the EuroQol-5 (EQ-5D, [91]), and Tamplin et al. [86] administered the Satisfaction with Life Scale (SWLS; [92]). Gallagher et al. [60] included a single-item (author’s own) to assess self-improvement in quality of life.

##### Findings

Garcia-Valverde et al. [76] and Tamplin et al. [86] reported an increase in quality of life from pre- to post music therapy intervention, which was statistically significant in Garcia-Valverde et al. [76] and represented a small to medium effect size across both studies. Gallagher et al. [60] with 50 family members of hospice patients, reported 83% to perceive self-improvement in their quality of life post-intervention. Mittelman and Papayannopoulou [84] reported a decrease in quality of life from pre-intervention to one-week post-intervention, with a medium effect size. In Tamplin et al. [63] with 44 family carers of individuals with Parkinson’s, there were no statistically significant differences reported between the music therapy groups (weekly and monthly singing group) or the control group in relation to quality of life.

#### Participation in work and/or other regular activities

##### Outcomes

One study [78] measured “self-efficacy” using the Revised Scale for Caregiving Self-Efficacy (RSCE; [93]). A number of studies (*n* = 7) assessed caregiver burden, a broader outcome domain encompassing constructs such as lifestyle burden in addition to social burden, emotional burden and physical burden. Four studies [59, 74, 78, 85] used either the Zarit Caregiver Burden Interview (ZBI; [94]) or short-form (ZBI-12; [95]). Brotons and Marti [72] administered the Caregiver Burden Questionnaire (CBQ; [96]), Hanser et al. [65] the Caregiving Satisfaction Scale (CSS; [97]) and Madso et al., the Relative Stress Scale (RSS; [98]).

##### Findings

Holden et al. [78] with 18 carers of individuals with dementia noted a non-significant increase in carer self-efficacy from baseline to 12 weeks post-music therapy intervention, with a large effect size. Six studies [59, 65, 72, 74, 78, 85] reported a decrease in caregiver burden over time, albeit only two reported a statistically significant change [59, 72]. For studies for whom effect size could be calculated, the decrease in caregiver burden reported either a small [74] or large effect size [78]. Särkämö et al. [59] reported a long-term effect on caregiver burden at six-months. One study [66] reported a non-significant increase in caregiver burden over time.

#### Relationships and social functioning (PGD risk factor- family conflict at EoL)

##### Outcomes

A number of studies (*n* = 7) assessed relationship quality between the carer and their close person. Four studies [63, 71, 74, 86] used the Quality of the Caregiver-Patient Relationship measure (QCPR; [99]) to assess overall relationship quality. Two studies [58, 84] measured communication using the Family Intimacy Observation Scale (FIOS; [58]) or the communication subscale of the Family Assessment Measure [100]. One study [64] measured communal behaviours using the Mutual Communal Behaviour Scale (MCBS; [101]).

##### Findings

Change in overall relationship quality was minimal across four studies. Three studies [71, 74, 86] reported stability in this construct and very small effect sizes. Tamplin et al. [63] reported no statistically significant differences between music therapy intervention and control groups for change in relationship quality. In relation to communication, Mittelman and Papayannopoulou [84] reported a non-significant increase in communication, with a small effect size. Kim and Dvorak [58] with 10 family carers of individuals receiving hospice care reported no statistically significant differences between the music therapy intervention and control group overall, but differences in specific communicative behaviours such as “verbally letting go of patient” where a large effect was observed. Baker et al. [64] with five spouses of individuals with dementia reported an increase in mutual communal behaviours from baseline to immediately after the intervention, with a small effect size.

#### Positive mental wellbeing (PGD protective factor- higher spirituality)

##### Outcomes

Seven studies assessed positive aspects of mental wellbeing, including positive aspects of caregiving. Three studies [64, 71, 86] administered the Positive Aspects of Caregiving measure (PACQ; [102]). Two studies [76, 84] measured self-esteem using the Rosenberg Self-Esteem Scale (RSS; [103]). Tamplin et al. [86] measured flourishing using the Flourishing Scale (FS; [104]). Hanser et al. [65] measured relaxation, comfort and happiness using visual analogue scales. Gallagher et al. [60] included a single-item (author’s own) to measure self-improvement in mood.

##### Findings

Studies reported a small increase [64] or small-moderate decrease [69, 86] in positive aspects of caregiving from pre- to post- music therapy intervention. Two studies [76, 84] reported a statistically significant increase in self-esteem from pre- to post-intervention, with a medium effect size. Gallagher et al. [60] reported that 92% of carers perceived self-improvement in their mood from the music therapy intervention at post-intervention. Hanser et al. [65] reported a statistically significant improvement in relaxation, comfort, and happiness after the majority of music therapy sessions.

#### Negative mental and emotional state (PGD risk factor- anxiety and depression)

##### Outcomes

Four studies [57, 59, 60, 63] assessed caregiver distress or mental health more broadly. To measure distress, Choi et al. [57] administered the Neuropsychiatric Inventory Questionnaire (NPI-Q; [105]) and Gallagher et al. [60] included a single-item (author’s own) to assess self-improvement in distress. To measure mental health more broadly, Särkämö et al. [59] administered the General Health Questionnaire-12 (GHQ-12; [106]) and Tamplin et al. [63] administered the Depression Anxiety, Stress Scale (DASS; [107]).

Five studies [62, 64, 72, 76, 85] specifically assessed anxiety. Two studies [72, 76] administered the Spielberger State-Trait Anxiety Inventory (STAI, [108]), one study [64] administered the Geriatric Anxiety Inventory (GAI; [109]) and one study [85] administered the Hamilton Rating Scale for Anxiety (HAM-A; [110]). Denk et al. [62] used a visual analogue single-item (author’s own) to assess anxiety.

The most commonly measured outcome for carers was depression, included as an outcome domain in nine studies. Three studies [71, 74, 86] administered the Public Health Questionnaire-9 (PHQ-9; [111]). Three studies [72, 76, 85] used different versions of Beck’s Depression Inventory; the BDI [112] and the BDI-II [113]. Two studies administered different versions of the Geriatric Depression Scale [64, 84]; the GDS [114] and GDS-SF [115]. Denk et al. [62] used a visual analogue single-item (author’s own) to assess depression.

##### Findings

Choi et al. [57] with 20 carers of individuals with dementia reported a significant difference between carer total distress score between the music therapy and control group, with a large effect size. Gallagher et al. [60] reported 94% of carers to perceive an improvement in their level of distress. Särkämö et al. [59] reported a non-significant difference in mental health between music therapy groups and control group at six-months post-intervention, with a large effect size. Tamplin et al. [63] reported a significant difference in depression between carers of individuals with Parkinson’s in a weekly singing group, compared to a monthly singing group. There were no differences between groups for anxiety.

In studies including a specific anxiety outcome domain, three studies [64, 72, 85] reported a decrease in anxiety from pre-to post music therapy intervention, albeit only Brotons and Marti [72] demonstrated a statistically significant change. Denk et al. [62] with four family carers of individuals with neurological conditions reported a decrease in anxiety from start to end of each music therapy session. Garcia-Valverde et al. [76] with 31 carers of individuals with dementia reported an increase in anxiety over time, with a medium effect size.

In studies including a specific depression outcome domain, four studies [62, 71, 72, 74] reported a decrease in depression from pre- to post music therapy intervention, with one study [72] reporting a statistically significant change. For studies for whom effect size could be calculated, the reduction in depression represented a small [74] or medium effect [71]. In three studies [84–86] there was little change in depression over time with levels remaining stable. Lastly, in two studies [64, 76] there was an increase in depression over time, representing a small to medium effect size.

### Results of the meta-synthesis of qualitative research findings

There were 22 studies which were either purely qualitative or which included a qualitative component. Alphanumeric code letters were assigned (A–X) however two studies (code letters J and W), were removed after extraction as it was determined that the available qualitative data were anecdotal [65], or that eligible participant data could not be separated [84]. Sample size was small overall, ranging from two to 31 participants.

Five synthesised findings emerged from informal carers’ perceptions and experiences with music therapy (Additional file 6). The synthesised findings were aggregated from 11 categories, with 191 study findings. Evidence for each finding was assessed, and findings categorised as unequivocal (*n* = 166), credible (*n* = 25), or unsupported (*n* = 23). Unsupported findings were extracted but not included in the synthesis. Some study findings were extracted and assigned an alphanumeric code, but were later assessed as being insufficiently relevant to bereavement and so were not included in the final synthesis. Meta-aggregation tables (Additional file 6) detail synthesised findings, categories, and extracted study findings.

### Synthesised finding 1: Social connectedness and social support

Two related categories comprising (*n* = 37) study findings were integrated into this synthesised finding. Study findings suggested that group therapeutic musical and singing interventions offered positive social opportunities which helped to reduce loneliness, both pre- and post-bereavement.

*“Participants also focused on Therapeutic Song Writing (TSW) as a positive social opportunity that was different to other group experiences. […] “the music allowed you to sort of interchange with other people .”* ([77], p.9)

Findings indicated that commonalities in experiences and connection with other group members helped to foster solidarity, empathy, and the sense of enjoying a “safe space” where other informal carers could explore and share their views and experiences.

*“It’s just a very comfortable feeling. Nobody is judging anybody else”* ([73], p.6)

*“a wonderful underlying thing that everybody knows what we’re all, what the baseline is underneath, and so you don’t have to explain it if you’re having a crap day, at all.”* ([87], p.16)

Study findings suggested that the participatory nature of the group supported a feeling of belonging, and that the group environment itself acted as a conduit for carers to express their feelings, elevating the opportunity for externalisation of feelings beyond what could be achieved in a one-to-one or dyad-music therapist setting.

*“We have all felt like one big family, sharing the pain, joys, tears, laughter. There is comprehension and affection in every moment experienced.”* ([77], p.8)

*“as a place “to be able to feel heard and understood and have support systems with other people.””* ([62], p.5)

### Synthesised finding 2: Music as an emotional/communication channel and spiritual bridge

Two related categories (study findings, *n* = 18) were integrated into this synthesised finding. Music therapy had a cathartic effect, eliciting and providing a safe channel for participants to experience, process, and communicate a range of grief-related emotions.

*“It’s an emotional shock to be able to share feelings and emotions. Thanks to these sessions, we learn to place and fit the pieces of the puzzle of our situation.”* ([77], p.11)

*“I get uplifted by the music and I feel I can let a lot of my feelings go into the music. It’s actual freedom [to sing] because I hadn’t sang at home for so long.”* ([61], p.60)

It was seen as a spiritual bridge to something beyond the bereavement experience, providing meaning and hope of something more beyond death, as well as a sense of connection to the deceased. It was considered a conduit, often particularly important/powerful at end of life, for enabling carers to transcend their bereavement experience and find peace.

*“When I heard the music, I heard the angels with us and felt like I was talking to God. I knew she [patient] was seeing her loved ones as we sang, even though they were somewhere beyond us. I know she is happy and in peace in that place now…”* ([82], p.102)

### Synthesised finding 3: Positive reminiscence of pre-illness identities/relationships and finding balance between grief and life going forward

Three related categories (study findings, *n* = 49) were integrated into this synthesised finding. Music therapy deepened relationships, and communication between the patient and carer. Music therapy was considered to be an activity which carers and patients could share.

*“It was nice to sit down together and listen to the music. Just to share some time together was good. I mean, it's harder to do this now.”* ([64], p.14)

This allowed them to interact in ways that resembled previous relationship dynamics, prior to the advent of the carer-patient relationship.

*“I think [it’s] better if, as we did, we talked about our previous life…it brought the past out of people who are now sitting in this environment… Because I think there’s a bit more happiness.”* ([74], p.10)

Carers were able to enjoy the patient experiencing the music, which could lead to the carer experiencing the patient as having a renewal of their former self, and they sometimes engaged in reflection, reminiscing, and life review together.

*“When he sings, he smiles. And he remembers the words, which I think is good for him. When he smiles it makes me happy and I can relax a bit.”* ([64], p.14)

*“In the beginning it was a great surprise and pleasure when the first time you came, and he was kind of comatose; he was with his eyes closed and he was not speaking, and you started playing and his lips started moving and then he starts singing, with a very soft voice, but he became alive again, and before he was not communicating with anyone; and more and more than in the other days, his voice was much stronger and he was obviously enjoying it tremendously, which was an incredible surprise because he was very passive and kind of like in a coma, detached with no response”* ([82], p.101)

This could also support the carer in enjoying positive memories of the patient. Music therapy activities were sometimes perceived to increase the carer's sense of connection to the patient and reignite/strengthen their relationship and sense of intimacy.

*“The music brought love back. I felt that love between us when I heard the music. Music brought us love…and joy. It’s so beautiful, that it gets into your heart, into love, everything. Her eyes were filled with so much love when we sang and we could all be close together again. At times she had seemed so far away and it meant so much to us all to be close and to have that love between us like that again”* ([81], p.37)

Additionally, reciprocity in the patient-carer relationship was perceived to be strengthened.

Collectively, reconnecting with their pre-illness/caregiver identities and deepened relationships with their significant other aided relationship completion and preparedness for death, which in turn helped them balance their grief with their renewed interest in life going forward.

*“And somehow…my sorrow, that I don’t want to rationalize away, has a bit turned into…gratitude for being able to be here. … This felt very, very good.”* ([68], p.5)

*“having done this experience (group song writing) will help me get stronger and get back into it and say “I’ve got a right to stay in the community”.* ([71], p.9)

*“I am looking forward to doing things that I want to now. Music, yoga. I am also debating between poets to read now. I have never been able to do that easily and my mother loved “The Leaves of Grass.” I have decided to read this. She [her mother/patient] really liked this. … It is to carry on with living”* ([82], p.104)

*“I refused [concert invitations] because I didn’t want people to know I was enjoying something…my husband is gone and I shouldn’t enjoy things. But now I’m okay. I want to do this and I’m allowing myself to go.”* ([61], p.62)

### Synthesised finding 4: Positive mental wellbeing: sense of meaning and purpose in life

Two related categories (study findings, *n* = 37) were integrated into this synthesised finding. Interventions offered and facilitated enjoyment of aesthetic qualities of music.

*“We could all see the pleasure he felt through his smiles and his looking at us as he sang with us…the creativity, the singing of harmonies. I was able to do something for him and bring him beauty.”* ([30], p.72)

Intervention offered a creative outlet which allowed participants to express meaningful inner emotions through creation of music and/or lyrics. Intervention offered routes of self-expression or connection, such as dance and moving to the music. Music could be used as a tool to regulate carers’ and patients’ moods—it could help to relax or to invigorate and was considered to have a positive impact on carer mood.

*“Before, I used to be [gesture of discouragement], and this has been wonderful for me because I always remember the sessions. We were very happy upon arriving and even more so when we left.”* ([77], p.11)

Some interventions encouraged use of music in daily life or otherwise when the intervention facilitator was not present, and it was considered to be of use as a caregiving tool to manage patient symptoms, including for gently bringing them back to life near EoL.

*“I was able to help give her the pleasure of music in the sessions and was able to give back to her the way she gave to me for so long. It was hard to see her in pain. Our singing brought her peace and comfort and I could smile and laugh again…”* ([82], p.100)

### Synthesised finding 5: Contextual and implementation factors

Two related categories (study findings, *n* = 50) were integrated into this synthesised finding. Flexible, tailored approaches helped to maximise participant comfort and engagement. Carer needs could differ according to the patient’s condition. Factors requiring consideration include intervention timing and duration, and group size. The goal of interventions was influential, for example, where social support was an objective, groups should be large enough to facilitate this and to be sustainable, but small enough to encourage interaction.

*“With the smaller group, you could toss [ideas] around and you got the yes or no… I think it’d be a lot harder if you tried to do it with just one couple. I think you needed that input from…other people as to their experiences and ideas.”* ([74], p.10)

Session regularity was valued. Carers of individuals with dementia who attended in dyads prefered short sessions or certain times of the day.

*“It’s hard to…you can’t have long really long sessions. They’ve got to be short sessions…because they just yeah, they can’t focus for a long time.”* ([74], p.10)

Participants had differing levels of musical experience, while loved ones experienced different stages of cognitive decline. It was important that activities were inclusive and delivered at an appropriate level.

*“It was great the way it was just very relaxed and free flowing. … If someone wanted to play, they could. If someone wanted to sing, they could. If someone wanted to dance, they could. … He [FC3’s Dad] could just be himself.”* ([80], p.6)

*“Mark felt that the singing warm-ups afforded opportunities to practice memory skills “…without feeling stupid about not knowing someone’s name.””* ([87], p.11)

Participant choice over songs and activities supported their engagement, and where a song was produced by the group, this was meaningful.

*“…there was a level of ownership about it, you felt included, as distinct from sitting back and watching. You can sort of opt out…[but] you almost have to opt-in.”* ([87], p.23)

*“Knowing that we’d completed it and, ah, hearing it, that was really the best part for me, I think. Knowing that we’d actually accomplished it.”* ([74], p.11)

The skills and characteristics of the music therapist themselves also influenced participants’ experiences. Empathy and a commitment to the therapeutic relationship were crucial.

*“I think the key is an empathetic leader. Our two leaders are compassionate and inspiring. The creativity of the group was brought out by the group leaders; writing songs and lyrics and melodies, sensitive to our input, meeting us where we are at.”* ([61], p. 60)

It was important that facilitators maintain an enthusiastic and knowledgeable presence which encouraged participation, but which also allowed the participants to take the lead. Participants appreciated when facilitators were organised and instructions clear.

*“But maybe, maybe things don’t happen by accident. They happened by good endeavour.*

*Hmm. Maybe it’s…partly the leadership of the group.”* ([87], p.23)

### Integration of quantitative and qualitative evidence

Findings from the qualitative and quantitative studies were juxtaposed to explore how qualitative findings map on to quantitative findings in relation to important outcomes and mechanisms of change. The newly developed core outcome set (COS) for evaluating bereavement interventions [6] was used as a framework to organise the integrated findings below (see Table 3 Postulated Music Therapy mechanisms of change and facilitating contexts mapped to a Bereavement Core Outcome Set [6] and risk and protective factors for Prolonged Grief Disorder [14]).

**Table 3:** Music Therapy mechanisms of change and facilitating context mapped to COS [6], PGD risk/protective factors [14]

Core outcome set for bereavement interventions	Risk and protective factors	Music therapy generated mechanisms of change	Facilitating context
**Ability to cope with grief**			
**Negative and overwhelming grief** • Feelings of loneliness and emptiness • Feelings of blame, guilt, anger, bitterness, regret • Overwhelming thoughts and/or nightmares about loss • Preoccupation with thoughts of the deceased	• Bereavement depression and anxiety	• Social connectedness and support from the group created a sense of belonging and reduced perceptions of loneliness	**Mode of delivery:** group music therapy (optimal size of the group should be discussed and agreed with participants) **Group characteristics:** non-judgemental, supportive space to share feelings and receive social support **Having a tangible output**: e.g., recording to listen back on their song creation **Facilitator/music therapist skills**: foster a sense of **self-efficacy** among caregiver participants, especially when the music therapy intervention involves song writing, and provide **leadership and support** to foster participation and engagement **Participant driven:** flexible, tailored approach to suit carers’ needs **Optimal dose**: Discuss and agree optimal dose e.g., some prefer six weekly sessions whereas others prefer a longer-term ongoing intervention **Collaborative/inclusive approach** (e.g., participants choose the musical content/songs)
**Communication and connectedness** • Ability to express feelings openly and honestly		• Musical catharsis: music therapy enabled carers to release repressed emotions	
• Feeling understood by and connected with other bereaved people	• Poor perceived social support	• Commonalities in experiences helped to foster solidarity, empathy, and the sense of enjoying a “safe space” to explore and share common feelings and needs.	
**Understanding, accepting and finding meaning in grief** • Acceptance of grief experiences as normal	• Early non-acceptance of loss/denial • perceived preparedness for death	• Music therapy aided relationship completion – helped caregiver accept/prepare for loss via renewed intimacy with their loved one	
• Understanding, acceptance, finding meaning in loss	• Higher spirituality	• Comfort, sense of meaning found in sharing/facilitating a music intervention that brought joy to their significant other/helped them reconnect and have hope in something more beyond bereavement	
• Positive reminiscence and remembering of the deceased	• Family conflict at EoL • Difficulty accessing positive memories	• Music therapy as a shared activity which improved caregiver/patient relationships • Positive reminiscence	
**Finding balance between grief and life going forwards** • Ability to find balance and channel grief • Ability to take control/ look ahead and start to move forward with life		• Music therapy provided an emotional channel for grief/increased preparedness for death of significant other • Motivated carers to re-engage/explore activities outside of the music therapy intervention going forward in their lives	
**Accessing appropriate support** • Accessing emotional support if needed • Accessing practical support if needed	• Poor perceived social support	• Group music therapy provided a safe, non-judgemental, supportive space to share feelings and receive social support	
**Quality of Life and Mental Wellbeing**			
**Participation in work and/or other regular activities** • Ability to perform daily tasks • Ability to participate in work • Ability to participate in social activities		• Motivated carers to re-engage/explore activities outside of the music therapy intervention going forward in their lives	
**Relationships and social functioning** • Ability to function as part of a family • Relationships with friends and family	• Family conflict at EoL	• Improved relationships with significant others/renewed connection and sense of intimacy	
**Positive mental wellbeing** • Sense of meaning and purpose in life • Optimism and hopefulness	• Higher spirituality	• Comfort, sense of meaning found in sharing/facilitating a music intervention that brought joy to their significant other/helped them reconnect and have purpose during pre-bereavement and hope in something more after bereavement	
**Negative mental and emotional state** • Anxiety (feelings of tension, nervousness, panic and distress)	• Anxiety	• Carers experienced relaxation, calmness and inner peace	
• Depression (a sense of hopelessness, pessimism, periods of crying)	• Bereavement depression	• Carers experienced improved mood, joy, happiness, a sense of achievement and contentment	
• Suicidal thoughts		• Music therapy gave carers a renewed sense of hope and resilience through restoring a balance between grief and moving on with their lives.	

First, *Ability to cope with grief*—Only one quantitative study [84] aligned to one of five categories within this domain—accessing appropriate support. This study found a non-significant increase in social support for family carers of individuals with dementia, although there was a medium effect. In explaining an increase in social support, qualitative findings indicate that group therapeutic musical and singing interventions offer positive social opportunities which helped to reduce loneliness, both pre- and post-bereavement. The mechanism of action appeared to be the sense of belonging generated by the participatory nature of the group environment.

Second, *Quality of Life and Mental Wellbeing—*The COS includes four categories with 10 dimensions for the Quality of Life and Mental Wellbeing domain. Both quantitative and qualitative data aligned with all four categories as follows. Quantitative findings were mixed for quality of life. Mittelman and Papayannopoulou [84] reported a decrease in quality of life, and another study [63] found no difference in quality of life between a music therapy intervention and control group. However, Garcia-Valverde et al. [76] and Tamplin et al. [86] reported increased quality of life, which was statistically significant in one study [76], with a small to medium effect size across both studies. Qualitative studies helped explain improved quality of life in these latter two studies via improvements across a diverse range of psychological, social and spiritual outcomes as presented in the following sections.

Third, *Participation in work and/or other regular activities*—Six studies [59, 65, 72, 74, 78, 85] reported a decrease in caregiver burden over time, albeit only two reported a statistically significant change [59, 72]. Whilst no qualitative studies specifically explored caregiver burden, there was evidence that the intervention environment itself acted as a conduit for carers to express their feelings, elevating the opportunity for externalisation of feelings beyond what could be achieved in a one-to-one or dyad-music therapist setting (Synthesised finding 1: Social connectedness and social support). This suggested intervention alleviation of caregiver burden across multiple constructs, in particular emotional and social (also Synthesised finding 2: Music as an emotional/communication channel and spiritual bridge).

Fourth, *Relationships and social functioning.* Seven quantitative studies assessed relationship quality between the carer and their close person [58, 63, 64, 71, 74, 84], however, change in overall relationship quality was minimal across the majority of these studies. In contrast, the qualitative studies suggested that music therapy deepened relationships by enhancing communication and reciprocity between the patient and carer, supported reminiscence, and offered a link to former identities. This assisted with relationship completion at end of life. (Synthesised finding 3: Positive reminiscence of pre-illness identities/relationships and finding balance between grief and life going forward). Only one quantitative study [84] addressed social support, reporting a non-significant change (t = 1.32, NS, d = 0.42) but a medium effect size. However, the importance of social connection and support was a key finding in the qualitative studies. Qualitative evidence indicated that interventions offered social connectedness and a reduction in loneliness, while intervention contexts offered safe spaces in which participants could share what they were experiencing and enjoy a sense of belonging amongst others in comparable situations (Synthesised finding 1: Social connectedness and social support).

Fifth, *Positive mental wellbeing.* Seven quantitative studies assessed positive aspects of mental wellbeing, including positive aspects of caregiving [60, 63–65, 71, 76, 84, 86]. These studies reported a small increase [64] or small-moderate decrease [71, 86] in positive aspects of caregiving from pre- to post- music therapy intervention. Similarly, several qualitative studies provided insight into the positive effects of music therapy interventions. For example, many interventions offered a creative outlet which encouraged emotional expression and uplifted carers’ moods. Some interventions encouraged use of music in daily life or otherwise when the intervention facilitator was not present, and it was considered to be of use as a caregiving tool to manage patient symptoms, including for gently bringing them back to life near EoL (Synthesised finding 4: Positive mental wellbeing: sense of meaning and purpose in life.)

Sixth, Negative mental and emotional state. Four quantitative studies assessed caregiver distress or mental health more broadly [57, 59, 60, 63]. More specifically, five quantitative studies [62, 64, 72, 76, 85] assessed anxiety whilst nine quantitative studies measured depression [62, 64, 71, 72, 74, 76, 84–86]. Choi et al. [57] with 20 carers of individuals with dementia reported a significant difference between carer total distress score between the music therapy and control group, with a large effect size. Gallagher et al. [60] reported 94% of carers to perceive an improvement in their level of distress. Särkämö et al. [59] reported a non-significant difference in mental health between music therapy groups and control group at six-months post-intervention, with a large effect size. Tamplin et al. [63] reported a significant difference in depression between carers of individuals with Parkinson’s disease in a weekly singing group, compared to a monthly singing group. There were no differences between groups for anxiety.

Three quantitative studies [64, 72, 85] reported a decrease in anxiety from pre-to post- music therapy intervention, albeit only Brotons and Marti [72] demonstrated a statistically significant change. Denk et al. [62] reported a decrease in anxiety from start to end of each intervention session. Garcia-Valverde et al. [76] reported an increase in anxiety over time, with a medium effect size.

In quantitative studies including a specific depression outcome domain, four studies [62, 71, 72, 74] reported a decrease in depression from pre- to post- music therapy intervention, with one study [72] reporting a statistically significant change. For studies for whom effect size could be calculated, the reduction in depression represented a small [74] or medium effect [71]. In three studies [84–86] there was little change in depression over time with levels remaining stable. Lastly, in two studies [64, 76] there was an increase in depression over time, representing a small to medium effect size.

The qualitative evidence helps to shed further light on these findings, with evidence across several studies indicating that music could be used as a tool to regulate carers’ and patients’ moods—it could help to relax or to invigorate and was considered to have a positive impact on carer mood (Synthesised finding 4: Positive mental wellbeing: sense of meaning and purpose in life). Qualitative evidence on the social aspect of group music therapy interventions suggests that this also offered mental health benefits, both in terms of reduced loneliness and an opportunity to vocalise complex feelings (Synthesised finding 1: Social connectedness and social support). Additionally, qualitative evidence on the benefits of music therapy for carers’ preparedness for death and balancing their grief with their future lives indicates that this supported resilience, feelings of hope, and re-engaging with their lives (Synthesised finding 3: Positive reminiscence of pre-illness identities/relationships and finding balance between grief and life going forward).

## Discussion

This mixed-methods segregated systematic review sought to describe the characteristics (including mechanisms of change, implementation processes, and economic considerations) and effectiveness of music therapy interventions which aim to improve health-related outcomes for adult informal carers of adults with life-threatening illness (pre- and post-bereavement) and to describe the experience of music therapy for adult informal carers of adults with life-threatening illness (pre- and post-bereavement).

The COS [6] was a useful framework for helping to identify gaps in the evidence base, potential mechanisms of change and key implementation considerations which should be tested in further research. The COS also orientates the findings within a resilience-based approach which is especially pertinent to music therapy bereavement work [31]. There was a dearth of quantitative research (only one study) exploring ability to cope with grief, specifically in relation to accessing appropriate support, even though this is a core outcome in bereavement research, and a risk factor for complicated grief. The qualitative literature supported the medium effect size for improvement in social support, albeit non-significant, suggesting it is social connectedness and a sense of belonging generated from being part of a community music therapy group that explains benefits. Social bonds appeared to have been strengthened through working toward achieving the same goal (shared music making). Music therapy’s ability to create a sense of community [116] and improve social interactions with family and friends has also been found in previous research [117–119]. However, more research is required to ascertain for whom (family carers of those living with which conditions?), and in what circumstances, i.e., what needs to be in place for music therapy to exert these effects.

The most commonly targeted outcomes in quantitative studies included quality of life and mental wellbeing core outcomes such as improvements in self-efficacy, communication, self-esteem, mood, relaxation, comfort, happiness, and reduction in caregiver burden, depression and anxiety. Findings were equivocal for the effectiveness of music therapy with significant and non-significant results found within and across all of these core outcomes. Explanations for effectiveness from qualitative studies suggested a wide and diverse range of improved outcomes for informal carers across all core outcomes, and across all risk and protective factors. Developments in neuro-scientific research further help explain how music therapy may exert benefits for mental wellbeing [120]. Several studies have demonstrated that music therapy reduces the activity of brain structures that impact on psychological processes including anxiety and emotional distress [121, 122], and have been further evidenced in the results of our meta-synthesis showing music therapy helped carers experience a sense of peace and happiness in the midst of their grief. Previous research also shows that improved communication between patients and families results in lower levels of depression and anxiety [123]. Music therapy’s ability to renew a sense of connection and intimacy between carers and their care recipient may help explain this widely reported finding in the meta-synthesis.

There was also a recurrent theme in the qualitative studies of music therapy helping carers reconnect with and express their pre-illness/caregiver identity as a spouse or child of the care recipient, through being offered a compassionate medium (shared music making) rather than a purely task-focused caregiver role. As theorised by the resource-orientated music therapy model developed for informal carers pre-bereavement [31], this resource helps build caregiver resilience, which is known to be a protective factor against complicated grief and a key marker of quality bereavement support.

Similarly to what has been found for music therapy for palliative care patients, music provided a safe channel for caregiver emotional expression [124] and an opportunity for sharing of existential/spiritual distress [125, 126] with others who understood what they were going through. Whether we identify as spiritual or not, this term has infiltrated discussions on health and wellbeing as far back as the 1980s. Although its meaning is not always clearly understood [127], in the healthcare literature it differs from the concept of religion [128]. For example, spirituality is more about “…the search for meaning and existential purpose in life” [129]1572, and this search for meaning is a recurring theme in the literature pertaining to the link between health and spirituality [127, 130–132].

We know that patients requiring palliative care can experience a deep sense of psychological and physical pain, even when physical pain is well controlled. We also know that this is “existential suffering” for which there is no medical solution [133]. With the increasing recognition that informal carers of individuals with life-threatening illness experience similar existential suffering, this has resulted in the move away from pathologising grief, and recommendations for universal, selective and specialist bereavement support services [4, 5]. Neimeyer [134] suggests that symptoms in the bereaved have meaning-making significance, and that meaning reconstruction in response to loss is the central process in grieving. This is where music therapy appears to exert its unique therapeutic benefits for carers. The meta-synthesis suggests that music therapy helps carers find meaning through similar mechanisms as those that facilitate emotional support – namely, enabling carers to reconnect with their pre-illness/caregiver identity as a spouse or child of the care recipient, through offering a compassionate medium to provide caregiving (shared music making) rather than the usual purely task-focused caregiver role [31]. Finding meaning in grief is considered a core outcome of any bereavement support intervention and is especially important for positive mental wellbeing [6].

The meta-synthesis also identified the key contextual influences to be considered when implementing a music therapy intervention for pre- and post-bereavement support for informal carers of individuals with life-threatening illness. As supported in previous research, a flexible, tailored approach is required [135]. If the intervention is delivered to a group, then the size of the group should be discussed and agreed with the anticipated service users, along with other practicalities such as the timing, length, and duration of the intervention. Another important, regularly reported consideration relates to musical/song choice [136] which should be participant-driven to achieve the best outcomes.

A key barrier to attendance at music therapy sessions for carers is wrapped up in the very nature of their 24/7 caregiver role which often prevents them having time for self-care [137]. Therefore, the music therapy intervention should be co-designed with carers so that it is tailored to their social and practical needs.

### Quality of the evidence base

The dichotomy between the evidence for benefit in qualitative compared to the mixed evidence of effectiveness in quantitative findings can be somewhat explained by methodological limitations. For example, effect sizes in quantitative studies ranged from small to large even in studies showing no statistically significant improvement, suggesting that the sample size was too small to detect a significant change [138]. Qualitative studies were generally of moderate – high quality, and quantitative studies of low – moderate quality. The lack of RCTs and high risk of bias in the small number of quantitative, largely quasi-experimental, studies to date limits confidence in the current quantitative evidence base and demonstrates a need to move towards high-quality randomised controlled trials in this area. Feasibility studies should include a power calculation to determine the appropriate sample size to detect change, or not, in subsequent RCTs. Every effort should also be made to include blinding procedures in future music therapy trial designs to reduce bias [139].

### Review limitations

This review’s findings are limited by the variable quality of studies to date in this field. There were also a number of challenges posed to integrating the evidence in this review. Overall, qualitative findings indicate that music therapy offers positive impacts in a variety of different areas including participants’ ability to cope with grief. However, this was not supported by quantitative study findings; few quantitative studies aligned to core outcome domains (and associated dimensions and risk factors) around the ability to cope with grief, and instead, most quantitative findings shared a mental health focus.

Additionally, whilst the qualitative findings provide detailed insight into the experience of music therapy for adult informal carers of adults with life-threatening illness pre-bereavement, no quantitative studies focused specifically on bereavement. This posed challenges when seeking to identify patterns of convergence and divergence between the qualitative and quantitative evidence. There was large heterogeneity in music therapy interventions in terms of content and delivery, and poor reporting. This limited comparison, or identification of which intervention approach works best for pre- and post-bereavement outcomes. The lack of consistency in the number of music therapy sessions across studies, combined with poor reporting of the duration of sessions also precludes any conclusions on the optimal dose for improved outcomes.

An additional complication was the overall lack of explicitly reported theories informing the included studies clinical interventions, methods, and/or processes at work. Without an underlying theoretical framework through which to understand findings, the clinical relevance – that is, the degree to which the study can directly apply to practice – of these studies is negatively impacted. It is possible that increased theoretical reporting could help future meta-analyses and narrative syntheses construct an integrated body of knowledge from the available literature.

There was a lack of standardised outcome measures, and high heterogeneity in outcome measures across all health-related outcomes. Out of nine studies for the most commonly reported outcome of depression, six different outcome measures were used. Representativeness of experiences was limited as most studies included informal carers for individuals with dementia and were conducted in America or Australia. Therefore, knowledge of the effectiveness of, and experience of music therapy for informal carers of adults at end of life (pre- and post-bereavement) with cancer and other non-cancer diagnoses is limited, as is the knowledge base in LMICs and the UK. The review was however limited to studies published in the English language and grey literature was excluded, so there is the possibility that research exists in LMICs which was not identified.

Further, there was a lack of longer-term follow-up studies, which limits our understanding of the benefit of music therapy interventions with this population in the longer term.

### Conclusions

Evidence of effectiveness in quantitative and qualitative studies is equivocal. Qualitative studies provide moderate to strong evidence for improved health-related outcomes for adult informal carers of adults with life-threatening illness pre-bereavement. The limited number of studies including the bereavement phase negates any conclusions. There is a high risk of bias and small samples in the limited number of quantitative, largely quasi-experimental, studies to date, which demonstrates a need to move towards high-quality randomised controlled trials in this area.

### Recommendations for future research

Given the high heterogeneity across all aspects of research to date, further research is needed to develop a best practice agenda for building and strengthening the evidence base for music therapy for adult informal carers of adults with life-threatening illness (pre- and post-bereavement). This is especially important for guiding best practice and commissioning of services. Further high-quality feasibility studies are needed to inform the conduct of high-quality trials, including identifying the best outcome measure(s) to use. Feasibility studies and trials should include a realist evaluation to develop a programme theory of what works (e.g., mechanisms of change), for whom and in what circumstances when implementing music therapy interventions to improve health-related outcomes for adult informal carers of adults with life-threatening illness (pre- and post-bereavement). Given the absence of any cost-effectiveness data to date, an economic evaluation/cost–benefit analysis should also be included.

The current research is heavily focused around dementia, highlighting the need for more research for informal carers of adults with cancer and other non-cancer diagnoses such as Parkinson’s, heart failure etc. The current research also predominantly includes dyadic interventions, targeting both patient and carer pre-bereavement. However. it is still an open question as to what the optimal timing of intervention is to improve outcomes for informal carers in this context. Research to examine different timings of music therapy intervention is warranted, alongside measurement of longer-term outcomes. Finally, more research in LMICs and the UK is required.

### Supplementary Information

**Additional file 1.** Search Strategies. Description of data: Systematic search strategies for all databases.

**Additional file 2.** Record of database searches up to July 2022.

**Additional file 3.** Qualitative Quality Appraisal Table. Results of methodological assessment of included qualitative articles.

**Additional file 4.** Quasi-Experimental Quality Appraisal Table. Results of methodological assessment of included quasi-experimental articles.

**Additional file 5.** RCT Quality Appraisal Table. Results of methodological assessment of included RCT articles.

**Additional file 6.** Meta-synthesis Tables. Tables show synthesised findings, categories, and extracted study findings.

## Data Availability

All data generated or analysed during this study are included in this published article [and its additional information files].

## Abbreviations

COS: Core outcome set
EoL: End-of-life/End of Life
EoLC: End-of-life care
IWD: Individual with Dementia
JBI: Joanne Briggs Institute
LMICs: Low- and middle-income countries
MMSR: Mixed-method systematic review
NHS: National Health Service
OSF: Open Science Framework
PCBD: Persistant Complex Bereavement Disorder
PTSD: Post Traumatic Stress Disorder
PGD: Prolonged grief disorder
RCT: Randomised controlled trial
UK: United Kingdom
WHO: World Health Organization
