# Supplementary material for: Music therapy for supporting informal carers of adults with life-threatening illness pre- and post-bereavement; a mixed-methods systematic review
Source: BMC Palliat Care. 2024 Feb 27;23:55. doi: 10.1186/s12904-024-01364-z (PMC10898157; doi:10.1186/s12904-024-01364-z)
Supplement: Supplementary file 1 — Additional file 1. Search Strategies. Description of data: Systematic search strategies for all databases. [file 12904_2024_1364_MOESM1_ESM.pdf]

MusiCARER search strategies

Ovid MEDLINE(R) ALL

1 Music/
2 Music Therapy/
3 Singing/
4 music$.tw,kf.
5 (sing or singing or song$ or choral$ or choir$).tw,kf.
6 (vibroacoustic$ or vibro-acoustic$).tw,kf.
7 (Bonny$ or guided imag$ or GIM or BMGIM).tw,kf.
8 or/1-7
9 adult children/
10 Caregivers/
11 Caregiver burden/
12 family/
13 Fathers/
14 grandparents/
15 Mothers/
16 Siblings/
17 Spouses/
18 nuclear family/
19 parents/
20 (carer$ or caregiver$ or care-giver$ or (service$ adj2 user$)).tw,kf.
21 ((brother$ or sister$ or sibling$ or son$ or daughter$) adj5 (care for or take care or taking care or informal$ care or caring or care-giving or caregiving)).tw,kf.
22 ((parent$ or mother$ or father$ or paternal or maternal) adj5 (care for or take care or taking care or informal$ care or caring or care-giving or caregiving)).tw,kf.
23 ((family or families or grandparent$ or grandmother$ or grandfather$ or grand-parent$ or grand-mother$ or grand-father$ or husband$ or partner$ or relative$1 or significant other$ or spouse$ or wife or wives) adj5 (care for or take care or taking care or informal$ care or caring or care-giving or caregiving)).tw,kf.
24 or/9-23
25 8 and 24
26 Advance Care Planning/
27 Attitude to death/
28 bereavement/
29 Death/
30 exp Euthanasia/ not Euthanasia,Animal/
31 Right to die/
32 exp Grief/
33 Hospice care/
34 Life Support Care/
35 Palliative Care/
36 exp Parental death/
37 Suicide, Assisted/
38 terminal care/
39 Terminally Ill/
40 (assisted suicide$ or assisted death).tw,kf.
41 (bereave$ or grief or griev$ or mourn$ or prebereavement or postbereavement or pre-loss or post-loss).tw,kf.
42 (death or dying).ti.
43 (end of life or end stage$).tw,kf.
44 hospice$.tw,kf.

45 palliative$.tw,kf.
46 ((terminal$ or lifelimit$ or life limit$ or life threaten$ or threat to life) adj3 (condition$ or disease$ or ill$)).tw,kf.
47 (terminal stage$1 or end stage$1 or late stage$1).tw,kf.
48 ((after$ or approach$ or before or close or near$) adj1 (death$ or dying)).tw,kf.
49 (advance$1 care adj1 (directive$ or plan$)).tw,kf.
50 or/26-49
51 8 and 50
52 25 or 51
53 limit 52 to english language
54 limit 53 to yr="1998 -Current"
55 exp animals/ not humans/
56 54 not 55

APA PsycInfo Ovid

1 music therapy/
2 music/
3 music perception/
4 musical instruments/
5 musical pitch/
6 singing/
7 (sing or singing or song$ or choral$ or choir$).tw.
8 (vibroacoustic$ or vibro-acoustic$).tw.
9 (Bonny$ or guided imag$ or GIM or BMGIM).tw.
10 or/1-9
11 caring behaviors/
12 Caregivers/
13 Caregiver Burden/
14 exp family members/
15 (carer$ or caregiver$ or care-giver$ or (service$ adj2 user$)).tw.
16 ((brother$ or sister$ or sibling$ or son$ or daughter$) adj5 (care for or take care or taking care or informal$ care or caring or care-giving or caregiving)).tw.
17 ((parent$ or mother$ or father$ or paternal or maternal) adj5 (care for or take care or taking care or informal$ care or caring or care-giving or caregiving)).tw.
18 ((family or families or grandparent$ or grandmother$ or grandfather$ or grand-parent$ or grand-mother$ or grand-father$ or husband$ or partner$ or significant other$ or spouse$ or wife or wives) adj5 (care for or take care or taking care or informal$ care or caring or care-giving or caregiving)).tw.
19 or/11-18
20 10 and 19
21 advance directives/
22 exp "death and dying"/
23 hospice/
24 Palliative Care/
25 terminal cancer/
26 terminally ill patients/
27 life sustaining treatment/

28 treatment refusal/
29 treatment withholding/
30 (assisted suicide$ or assisted death).tw.
31 (bereave$ or grief or griev$ or mourn$ or prebereavement or postbereavement or pre-loss or post-loss).tw.
32 (death or dying).ti.
33 (end of life or end stage$).tw.
34 hospice$.tw.
35 palliative$.tw.)
36 ((terminal$ or lifelimit$ or life limit$ or life threaten$ or threat to life) adj3 (condition$ or disease$ or ill$)).tw.
37 (terminal stage$1 or end stage$1 or late stage$1).tw.
38 ((after$ or approach$ or before or close or near$) adj1 (death$ or dying)).tw.
39 (advance$1 care adj3 (directiv$ or plan$)).tw.
40 or/21-39
41 10 and 40
42 20 or 41
43 limit 42 to english language
44 limit 43 to yr="1998 -Current"
45 limit 44 to animal (Annotation: records retrieved by line 45 were checked manually to ensure no relevant human records were excluded)
46 44 not 45

OVID Embase

1 exp music/
2 music therapy/
3 *singing/
4 music$.tw,kw.
5 (sing or singing or song$ or choral$ or choir$).tw,kw.
6 (vibroacoustic$ or vibro-acoustic$).tw,kw.
7 (Bonny$ or guided imag$ or GIM or BMGIM).tw,kw.
8 or/1-7
9 adult child/
10 caregiver/
11 caregiver burden/
12 extended family/
13 family/
14 family relation/
15 father/
16 exp grandparent/
17 mother/
18 parent/
19 exp sibling/
20 exp spouse/
21 nuclear family/
22 (carer$ or caregiver$ or care-giver$ or (service$ adj2 user$)).tw,kw.
23 ((brother$ or sister$ or sibling$ or son$ or daughter$) adj5 (care for or taking care or informal$ care or caring or care- giving or caregiving)).tw,kw.

24 ((parent$ or mother$ or father$ or paternal or maternal) adj5 (care for or taking care or informal$ care or caring or care- giving or caregiving)).tw,kw.
25 ((family or families or grandparent$ or grandmother$ or grandfather$ or grand-parent$ or grand-mother$ or grand-father$ or husband$ or partner$ or significant other$ or spouse$ or wife or wives) adj5 (care for or taking care or informal$ care or caring or care-giving or caregiving)).tw,kw.
26 or/9-25
27 8 and 26
28 advance care planning/
29 attitude to death/
30 bereavement/
31 bereavement support/
32 death/
33 exp euthanasia/ not Animal Euthanasia/
34 right to die/
35 exp grief/
36 hospice/
37 hospice care/
38 hospice nursing/
39 hospice patient/
40 exp palliative therapy/
41 exp parental death/
42 assisted suicide/
43 terminal care/
44 terminally ill patient/
45 (assisted suicide$ or assisted death).tw,kw
46 (bereave$ or grief or griev$ or mourn$ or prebereavement or postbereavement or pre-loss or post-loss).tw,kw.
47 (death or dying).ti.)
48 (end of life or end stage$).tw,kw.
49 hospice$.tw,kw.
50 palliative$.tw,kw.
51 ((terminal$ or lifelimit$ or life limit$ or life threaten$ or threat to life) adj3 (condition$ or disease$ or ill$)).tw,kw.
52 (terminal stage$1 or end stage$1 or late stage$1).tw,kw.
53 ((after$ or approach$ or before or close or near$) adj1 (death$ or dying)).tw,kw.
54 (advance$1 care adj1 (directive$ or plan$)).tw,kw.
55 or/28-54
56 8 and 55
57 27 or 56
58 exp animals/ or exp invertebrate/ or animal experiment/ or animal model/ or animal tissue/ or animal cell/ or nonhuman/
59 human/ or normal human/ or human cell/
60 58 and 59
61 58 not 60
62 57 not 61
63 limit 62 to (english language and yr="1998 -Current")
64 limit 63 to (embase or medline) Annotation: This limit excludes pre-prints and conference proceedings

CINAHL Plus EBSCOhost

S1 (MH "Music")
S2 (MH "Music Therapy")
S3 (MH "Singing")
S4 TI(music*) OR AB(music*)
S5 TI(sing or singing or song* or choral* or choir*) OR AB(sing or singing or song* or choral* or choir*)
S6 TI(Bonny* or "guided imag*" or GIM or BMGIM) OR AB(Bonny* or "guided imag*" or GIM or BMGIM)
S7 S1 OR S2 OR S3 OR S4 OR S5 OR S6
S8 (MH "Adult Children")
S9 Caregivers
S10 (MH "Caregivers")
S11 (MH "Caregiver Burden")
S12 (MH "Family") OR (MH "Extended Family")
S13 (MH "Fathers")
S14 (MH "Grandparents")
S15 (MH "Mothers")
S16 (MH "Siblings")
S17 (MH "Spouses")
S18 (MH "Nuclear Family")
S19 (MH "Parents")
S20 (MH "Widows and Widowers")
S21 TI(carer* or caregiver* or care-giver* or (service* N2 user*)) OR AB(carer* or caregiver* or care-giver* or (service* N2 user*))
S22 TI((brother* or sister* or sibling* or son* or daughter*) N5 ("care of" or "take care" or "taking care" or "informal* care" or caring or care-giving or caregiving)) OR AB((brother* or sister* or sibling* or son* or daughter*) N5 ("care of" or "take care" or "taking care" or "informal* care" or caring or care-giving or caregiving))
S23 TI(parent* or mother* or father* or paternal or maternal) N5 ("care of" or "take care" or "taking care" or "informal* care" or caring or care-giving or caregiving)) OR AB(parent* or mother* or father* or paternal or maternal) N5 ("care of" or "take care" or "taking care" or "informal* care" or caring or care-giving or caregiving))
S24 TI((family or families or grandparent* or grandmother* or grandfather* or grand-parent* or grand-mother* or grand-father* or husband* or partner* or relative* or "significant other*" or spouse* or wife or wives) N5 ("care of" or "take care" or "taking care" or "informal* care" or caring or care-giving or caregiving)) OR AB((family or families or grandparent* or grandmother* or grandfather* or grand-parent* or grand-mother* or grand-father* or husband* or partner* or relative* or "significant other*" or spouse* or wife or wives) N5 ("care of" or "take care" or "taking care" or "informal* care" or caring or care-giving or caregiving))

S25 S8 OR S9 OR S10 OR S11 OR S12 OR S13 OR S14 OR S15 OR S16 OR S17 OR S18 OR S19 OR S20 OR S21 OR S22 OR S23 OR S24
S26 S7 AND S25
S27 (MH "Advance Care Planning")
S28 (MH "Attitude to Death")
S29 (MH "Bereavement")
S30 (MH "Death")
S31 (MH "Euthanasia") OR (MH "Euthanasia, Passive") OR (MH "Suicide, Assisted")
S32 (MH "Right to Die")

S33 (MH "Grief+")
S34 (MH "Hospice Care") OR (MH "Hospice Patients") OR (MH "Hospice and Palliative Nursing"

S35 (MH "Life Support Care")
S36 (MH "Palliative Care")
S37 (MH "Parental Death")
S38 (MH "Personal Loss")
S39 (MH "Terminal Care") OR (MH "Terminally Ill Patients")
S40 TI("assisted suicide*" or "assisted death") OR AB("assisted suicide*" or "assisted death"

S41 TI(bereave* or grief or griev* or mourn* or prebereavement or postbereavement or "pre-loss" or "post-loss") OR AB(bereave* or grief or griev* or mourn* or prebereavement or postbereavement or "pre-loss" or "post-loss")
S42 TI (death or dying)
S43 TI("end of life" or "end stage*") OR AB("end of life" or "end stage*")
S44 TI(hospice*) OR AB(hospice*)
S45 TI(palliative*) OR AB(palliative*)
S46 TI((terminal* or lifelimit* or "life limit*" or "life threaten*" or "threat to life") N3 (condition* or disease* or ill*) OR AB((terminal* or lifelimit* or "life limit*" or "life threaten*" or "threat to life") N3 (condition* or disease* or ill*)
S47 TI(terminal stage* or end stage* or late stage*) OR AB(terminal stage* or end stage* or late stage*)
S48 TI((after* or approach* or before or close or near*) N1 (death* or dying)) OR AB((after* or approach* or before or close or near*) N1 (death* or dying))
S49 TI(advance* care N1 (directive* or plan*)) OR AB(advance* care N1 (directive* or plan*))

S50 S27 OR S28 OR S29 OR S30 OR S31 OR S32 OR S33 OR S34 OR S35 OR S36 OR S37 OR S38 OR S39 OR S40 OR S41 OR S42 OR S43 OR S44 OR S45 OR S46 OR S47 OR S48 OR S49
S51 S7 AND S50
S52 S26 OR S51
S53 S26 OR S51 Narrow by Language: english
S54 S26 OR S51 Limiters - Publication Year: 1998-2022 Narrow by Language: english

RILM Abstracts of Music Literature (EBSCOhost)

S1 music* N5 (group* or inpatient* or therap* or intervention* or participat* or participant* or patient* or program* or treatment* or workshop*)
S2 (harmony or harmonies or harmonis* or harmoniz*) N5 (group* or inpatient* or therap* or intervention* or participat* or participant* or patient* or program* or treatment* or workshop*)
S3 (improvis* N5 (group* or inpatient* or therap* or intervention* or participant* or participat* or patient* or program* or treatment* or workshop*)
S4 ((melody or melodies or tune*) N5 (group* or inpatient* or therap* or intervention* or participat* or participant* or patient* or program* or treatment* or workshop))
S5 (percussion* N5 (group* or inpatient* or therap* or intervention* or participat* or participant* or patient* or program* or treatment* or workshop))
S6 (pitch N5 (group* or inpatient* or therap* or intervention* or participat* or participant* or patient* or program* or treatment* or workshop))
S7 (rhythm* N5 (group* or inpatient* or therap* or intervention* or participat* or participant* or patient* or program* or treatment* or workshop))

S8 (sing or singing or song* or choral* or choir*) N5 (group* or inpatient* or therap* or intervention* or participat* or participant* or patient* or program* or treatment* or workshop)
S9 (tempo N5 (group* or inpatient* or therap* or intervention* or participat* or patient* or program* or treatment* or workshop))
S10 (timbre N5 (group* or inpatient* or therap* or intervention* or participat* or patient* or program* or treatment* or workshop))
S11 ((vibroacoustic* or vibro-acoustic*) N5 (group* or inpatient* or therap* or intervention* or participat* or patient* or program* or treatment* or workshop))
S12 ((Bonny* or "guided imag*" or GIM or BMGIM) N5 (group* or inpatient* or therap* or intervention* or participat* or patient* or program* or treatment* or workshop*))
S13 S1 OR S2 OR S3 OR S4 OR S5 OR S6 OR S7 OR S8 OR S9 OR S10 OR S11 OR S12
S14 (carer* or caregiver* or care-giver* or (service* N2 user*))
S15 ((brother* or sister* or sibling* or son* or daughter*) N5 ("care of" or "take care" or "taking care" or "informal* care" or caring or care-giving or caregiving))
S16 (parent* or mother* or father* or paternal or maternal) N5 ("care of" or "take care" or "taking care" or "informal* care" or caring or care-giving or caregiving))
S17 ((family or families or grandparent* or grandmother* or grandfather* or grand-parent* or grand-mother* or grand-father* or husband* or partner* or relative* or "significant other*" or spouse* or wife or wives) N5 ("care of" or "take care" or "taking care" or "informal* care" or caring or care-giving or caregiving))
S18 S14 OR S15 OR S16 OR S17
S19 S13 and S18
S20 ("assisted suicide*" or "assisted death")
S21 (bereave* or grief or griev* or mourn* or prebereavement or postbereavement or "pre-loss" or "post-loss")
S22 TI(death OR dying)
S23 ("end of life" or "end stage*")
S24 ("advance* care" N3 (directiv* or plan*))
S25 ((after* or approach* or before or close or near*) N1 (death* or dying))
S26 hospice* or palliative or "good death" or "death with dignity" or "dignified death"
S27 ((terminal* or lifelimit* or "life limit*" or "life threaten*" or "threat to life") N3 (condition* or disease* or ill*)
S28 ("terminal stage*" or "late stage*")
S29 S20 OR S21 OR S22 OR S23 OR S24 OR S25 OR S26 OR S27 OR S28
S30 S13 AND S29
S31 S19 OR S30
S32 S19 OR S30 Limiters - Publication Date: 19980101-20221231
S33 S19 OR S30 Limiters - Publication Date: 19980101-20221231 Narrow by Language: - english

Cochrane Central Register of Controlled Trials (CENTRAL) searched using Cochrane Register of Studies Online (https://crso.cochrane.org/home.php)

#1 MESH DESCRIPTOR music EXPLODE ALL TREES

#2 MESH DESCRIPTOR music therapy EXPLODE ALL TREES

#3 music*:TI,AB,KY
#4 (vibroacoustic* or vibro NEXT acoustic*):TI,AB,KY

#5 ((Bonny* or guided NEXT imag* or GIM or BMGIM)):TI,AB,KY

#6 MESH DESCRIPTOR Singing EXPLODE ALL TREES

#7 ((sing or singing or song* or choral* or choir*)):TI,AB,KY

#8 #1 OR #2 OR #3 OR #4 OR #5 OR #6 OR #7

#9 MESH DESCRIPTOR adult children EXPLODE ALL TREES

#10 MESH DESCRIPTOR Caregivers EXPLODE ALL TREES

#11 MESH DESCRIPTOR Caregiver Burden EXPLODE ALL TREES

#12 MESH DESCRIPTOR Family EXPLODE ALL TREES

#13 MESH DESCRIPTOR Fathers EXPLODE ALL TREES

#14 MESH DESCRIPTOR Grandparents EXPLODE ALL TREES

#15 MESH DESCRIPTOR Mothers EXPLODE ALL TREES

#16 MESH DESCRIPTOR Siblings EXPLODE ALL TREES

#17 MESH DESCRIPTOR Spouses EXPLODE ALL TREES

#18 MESH DESCRIPTOR nuclear family EXPLODE ALL TREES

#19 MESH DESCRIPTOR parents EXPLODE ALL TREES

#20 (((parent* or mother* or father* or paternal or maternal) NEAR5 (care NEXT for OR take NEXT care or taking NEXT care or informal* NEXT care or caring or care NEXT giving or caregiving)) or ((care NEXT for OR take NEXT care or taking NEXT care or informal* NEXT care or caring or care NEXT giving or caregiving) NEAR5 (parent* or mother* or father* or paternal or maternal))):TI,AB,KY

#21 (((brother* or sister* or sibling* or son* or daughter*) NEAR5 (care NEXT for OR take NEXT care or taking NEXT care or informal* NEXT care or caring or care NEXT giving or caregiving)) or ((care NEXT for OR take NEXT care or taking NEXT care or informal* NEXT care or caring or care NEXT giving or caregiving) NEAR5 (brother* or sister* or sibling* or son* or daughter*))):TI,AB,KY

#22 (((family or families or grandparent* or grandmother* or grandfather* or grand NEXT parent* or grand NEXT mother* or grand NEXT father* or husband* or partner* or relative* or significant NEXT other* or spouse* or wife or wives) NEAR5 (care NEXT for OR take NEXT care or taking NEXT care or informal* NEXT care or caring or care NEXT giving or caregiving)) OR ((care NEXT for OR take NEXT care or taking NEXT care or informal* NEXT care or caring or care NEXT giving or caregiving) NEAR5 (family or families or grandparent* or grandmother* or grandfather* or grand NEXT parent* or grand NEXT mother* or grand NEXT father* or husband* or partner* or relative* or significant NEXT other* or spouse* or wife or wives))):TI,AB,KY

#23 (carer* or caregiver* or care-giver* or (service* NEAR2 user*) or (user* NEAR2 service*)):TI,AB,KY

#24 #9 OR #10 OR #11 OR #12 OR #13 OR #14 OR #15 OR #16 OR #17 OR #18 OR #19 OR #20 OR #21 OR #22 OR #23
#25 #8 AND #24
#26 MESH DESCRIPTOR Advance Care Planning EXPLODE ALL TREES

#27 MESH DESCRIPTOR Attitude to death EXPLODE ALL TREES

#28 MESH DESCRIPTOR bereavement EXPLODE ALL TREES

#29 MESH DESCRIPTOR Death EXPLODE ALL TREES

#30 MESH DESCRIPTOR Euthanasia

#31 MESH DESCRIPTOR Right to die

#32 MESH DESCRIPTOR Grief EXPLODE ALL TREES

#33 MESH DESCRIPTOR Hospice care EXPLODE ALL TREES

#34 MESH DESCRIPTOR Life Support Care EXPLODE ALL TREES

#35 MESH DESCRIPTOR Palliative Care EXPLODE ALL TREES

#36 MESH DESCRIPTOR Parental death EXPLODE ALL TREES

#37 MESH DESCRIPTOR Suicide, Assisted EXPLODE ALL TREES

#38 MESH DESCRIPTOR terminal care EXPLODE ALL TREES

#39 MESH DESCRIPTOR Terminally Ill EXPLODE ALL TREES

#40 ((assisted NEXT suicide* or assisted NEXT death)):TI,AB,KY

#41 ((bereave* or grief or griev* or mourn* or prebereavement or postbereavement or pre NEXT loss or post NEXT loss)):TI,AB,KY

#42 (death or dying):TI
#43 hospice*:TI,AB,KY
#44 palliative*:TI,AB,KY
#45 (((after* or approach* or before or close or near*) NEAR1 (death* or dying)) or ((death* or dying) NEAR1 (after* or approach* or before or close or near*))):TI,AB,KY

#46 (((terminal* or lifelimit* or life NEXT limit* or life NEXT threaten* or "threat to life") NEAR3 (condition* or disease* or ill*)) OR ((condition* or disease* or ill*) NEAR3 (terminal* or lifelimit* or life NEXT limit* or life NEXT threaten* or "threat to life"))):TI,AB,KY

#47 ((terminal NEXT stage* or end NEXT stage* or late NEXT stage*)):TI,AB,KY

#48 ((advance* NEXT care NEAR1 (directive* or plan*))):TI,AB,KY

#49 #26 OR #27 OR #28 OR #29 OR #30 OR #31 OR #32 OR #33 OR #34 OR #35 OR #36 OR #37 OR #38 OR #39 OR #40 OR #41 OR #42 OR #43 OR #44 OR #45 OR #46 OR #47 OR #48

#50 #8 AND #49
#51 #25 OR #50
#52 1998 TO 2022:YR
#53 #51 AND #52
