# Supplementary material for: Music therapy for supporting informal carers of adults with life-threatening illness pre- and post-bereavement; a mixed-methods systematic review
Source: BMC Palliat Care. 2024 Feb 27;23:55. doi: 10.1186/s12904-024-01364-z (PMC10898157; doi:10.1186/s12904-024-01364-z)
Supplement: Supplementary file 2 — Additional file 2. Record of database searches up to July 2022. [file 12904_2024_1364_MOESM2_ESM.pdf]

MusiCARER systematic review. Record of database searches up to July 2022

Database	Search date	Database date range	Number of records
Cochrane Central Register of Controlled Trials (CENTRAL) via Cochrane Register of Studies Online (https://crso.cochrane.org/)	15 April 2021	Current issue	**323**
Cochrane Central Register of Controlled Trials (CENTRAL) via Cochrane Register of Studies Online (https://crso.cochrane.org/)	19 July 2022	Current issue	**395**
Ovid MEDLINE ALL	15 April 2021	1946 to April 14, 2021	**1299**
Ovid MEDLINE ALL	19 July 2022	1946 to July 18, 2022	**1638**
Embase (Ovid)	15 April 2021	1974 to 2021 April 14	**1741**
Embase (Ovid)	19 July 2022	1974 to 2022 July 18	**2065**
APA PsycINFO (Ovid)	15 April 2021	1806 to April Week 1 2021	**1198**
APA PsycINFO (Ovid)	19 July 2022	1806 to July Week 2 2022	**1406**
CINAHL Plus (EBSCOhost)	15 April 2021	1939 onwards	**1694**
CINAHL Plus (EBSCOhost)	19 July 2022	1939 onwards	**1981**
RILM Abstracts of Music Literature (EBSCOhost)	15 April 2021	Current issue	**1097**
RILM Abstracts of Music Literature (EBSCOhost)	19 July 2022	Current issue	**963**
		**TOTAL RECORDS**	**15,800**
