# Supplementary material for: Music therapy for supporting informal carers of adults with life-threatening illness pre- and post-bereavement; a mixed-methods systematic review
Source: BMC Palliat Care. 2024 Feb 27;23:55. doi: 10.1186/s12904-024-01364-z (PMC10898157; doi:10.1186/s12904-024-01364-z)
Supplement: Supplementary file 3 — Additional file 3. Qualitative Quality Appraisal Table. Results of methodological assessment of included qualitative articles. [file 12904_2024_1364_MOESM3_ESM.docx]

**Additional file 3**

Additional file 3: Results of methodological assessment of included qualitative articles

|  |  | **Question** | | | | | | | | | |  |
| --- | --- | --- | --- | --- | --- | --- | --- | --- | --- | --- | --- | --- |
|  | **Citation** | **1** | **2*** | **3*** | **4*** | **5** | **6*** | **7*** | **8** | **9** | **10** | % |
| 1 | Baker et al. (2018) (1) | Y | Y | Y | Y | Y | Y | Y | Y | Y | Y | 100% |
| 2 | Black et al. (2020) (2) | Y | Y | Y | U | U | N | Y | Y | Y | Y | 70% |
| 3 | Clark et al. (2018)# (3) | Y | Y | Y | Y | Y | Y | Y | Y | Y | Y | 100% |
| 4 | Clark et al. (2021) (4) | Y | Y | Y | Y | Y | Y | Y | Y | Y | Y | 100% |
| 5 | Dassa et al. (2020) (5) | Y | Y | Y | Y | Y | Y | Y | Y | Y | Y | 100% |
| 6 | García-Valverde et al. (2022) (6) | Y | Y | Y | Y | Y | Y | Y | Y | Y | Y | 100% |
| 7 | Lee et al. (2022) (7) | Y | Y | Y | Y | Y | N | U | Y | Y | Y | 80% |
| 8 | Magill (2009a) (8) | Y | Y | Y | Y | Y | N | N | Y | Y | Y | 80% |
| 9 | Magill (2009b) (9) | Y | Y | Y | Y | Y | Y | Y | Y | N | Y | 90% |
| 10 | Magill (2009c) (10) | Y | Y | Y | Y | Y | N | N | Y | NA | Y | 70% |
| 11 | Magill (2011) (11) | Y | Y | Y | Y | Y | Y | Y | Y | Y | Y | 100% |
| 12 | O’Callaghan et al. (2013) (12) | Y | Y | Y | Y | Y | N | Y | Y | Y | Y | 90% |
| 13 | Potvin et al. (2018) (13) | Y | Y | Y | Y | Y | Y | Y | Y | Y | Y | 100% |
| 14 | Teut et al. (2014) (14) | Y | Y | Y | Y | Y | N | Y | Y | Y | Y | 90% |
| 15 | Young et al. (2018) (15) | Y | Y | Y | Y | Y | Y | Y | Y | Y | Y | 100% |
| 16 | Thompson et al. (2022) (16) | Y | Y | Y | Y | Y | Y | Y | Y | Y | Y | 100% |
| 17 | Klein et al. (2012) (17) | U | U | Y | Y | Y | N | N | N | Y | Y | 50% |
|  |  | 94% | 94% | 100% | 94% | 94% | 59% | 76% | 94% | 94% | 100% |  |
| **Mixed Method Studies Qualitative Component** | | | | | | | | | | | |  |
|  | **Citation** | **1** | **2*** | **3*** | **4*** | **5** | **6*** | **7*** | **8** | **9** | **10** | % |
| 1 | Baker et al. (2018) (18) | Y | Y | Y | Y | Y | N | N | Y | Y | Y | 80% |
| 2 | Baker et al. (2012) (19) | Y | Y | Y | Y | Y | N | N | Y | Y | Y | 80% |
| 3 | Clark et al. (2020) (20) | U | Y | Y | U | Y | N | N | Y | Y | Y | 60% |
| 4 | Hanser et al. (2011)* (21) | U | Y | U | U | U | N | N | N | Y | U | 20% |
| 5 | Denk et al. (2022) (22) | U | Y | Y | N | Y | N | N | Y | Y | Y | 60% |
| 6 | Mittleman et al (2018)† (23) | U | Y | N | N | N | N | N | Y | Y | Y | 40% |
| 7 | Tamplin et al. (2018)# (24) |  |  |  |  |  |  |  |  |  |  |  |
|  |  | 33% | 100% | 67% | 33% | 67% | 0% | 0% | 83% | 100% | 83% |  |

Y, Yes; no, No; U, Unclear; N/A, Not Applicable. * Dependability questions.

1.             Is there congruity between the stated philosophical perspective and the research methodology?

2.             Is there congruity between the research methodology and the research question or objectives?

3.             Is there congruity between the research methodology and the methods used to collect data?

4.             Is there congruity between the research methodology and the representation and analysis of data?

5.             Is there congruity between the research methodology and the interpretation of results?

6.             Is there a statement locating the researcher culturally or theoretically?

7.             Is the influence of the researcher on the research, and vice- versa, addressed?

8.             Are participants, and their voices, adequately represented?

9.             Is the research ethical according to current criteria or, for recent studies, and is there evidence of ethical approval by an appropriate body?

10.          Do the conclusions drawn in the research report flow from the analysis, or interpretation, of the data?

#Tamplin et al.’s (2018) qualitative component reported in full in Clark et al., (2018)

*Removed from meta-synthesis as available qualitative data were anecdotal

†Removed from meta-synthesis as eligible participant data could not be separated

**References**

1. Baker FA, Yeates S. Carers’ experiences of group therapeutic songwriting: An interpretive phenomenological analysis. Br J Music Ther. 2018;32(1):8–17.

2. Black S, Bartel L, Rodin G. Exit Music: The Experience of Music Therapy within Medical Assistance in Dying. Healthcare. 2020 Sep 10;8(3):331.

3. Clark IN, Tamplin JD, Baker FA. Community-Dwelling People Living With Dementia and Their Family Caregivers Experience Enhanced Relationships and Feelings of Well-Being Following Therapeutic Group Singing: A Qualitative Thematic Analysis. Front Psychol. 2018 Jul 30;9(July):1–13.

4. Clark IN, Baker FA, Tamplin J, Lee Y-EC, Cotton A, Stretton-Smith PA. “Doing Things Together Is What It’s About”: An Interpretative Phenomenological Analysis of the Experience of Group Therapeutic Songwriting From the Perspectives of People With Dementia and Their Family Caregivers. Front Psychol. 2021 Mar 31;12(March):1–15.

5. Dassa A, Rosenbach M, Gilboa A. Towards sustainable implementation of music in daily care of people with dementia and their spouses. Arts Psychother. 2020 Nov;71(September):101713.

6. García-Valverde E, Badia Corbella M, Orgaz Baz MB. Experiences of group therapeutic songwriting of family caregivers of people with dementia. Psychol Music. 2022;50(2):530–47.

7. Lee S, O’Neill D, Moss H. Promoting well-being among people with early-stage dementia and their family carers through community-based group singing: a phenomenological study. Arts Health. 2022 Jan 2;14(1):85–101.

8. Magill L. The Meaning of the Music: The Role of Music in Palliative Care Music Therapy as Perceived by Bereaved Caregivers of Advanced Cancer Patients. Am J Hosp Palliat Med. 2009 Feb 8;26(1):33–9.

9. Magill L. The spiritual meaning of pre-loss music therapy to bereaved caregivers of advanced cancer patients. Palliat Support Care. 2009;7(1):97–108.

10. Magill L. Caregiver Empowerment and Music Therapy: Through the Eyes of Bereaved Caregivers of Advanced Cancer Patients. J Palliat Care. 2009 Mar 19;25(1):68–75.

11. Magill L. Bereaved Family Caregivers’ Reflections on the Role of the Music Therapist. Music Med. 2011 Jan 1;3(1):56–63.

12. O’Callaghan CC, McDermott F, Hudson P, Zalcberg JR. Sound Continuing Bonds with the Deceased: The Relevance of Music, Including Preloss Music Therapy, for Eight Bereaved Caregivers. Death Stud. 2013 Feb;37(2):101–25.

13. Potvin N, Bradt J, Ghetti C. A Theoretical Model of Resource-Oriented Music Therapy with Informal Hospice Caregivers during Pre-Bereavement. J Music Ther. 2018 Mar 9;55(1):27–61.

14. Teut M, Dietrich C, Deutz B, Mittring N, Witt CM. Perceived outcomes of music therapy with Body Tambura in end of life care – a qualitative pilot study. BMC Palliat Care. 2014 Dec 7;13(1):18.

15. Young L, Pringle A. Lived experiences of singing in a community hospice bereavement support music therapy group. Bereave Care. 2018 May 4;37(2):55–66.

16. Thompson Z, Tamplin J, Clark I, Baker F. Therapeutic Choirs for Families Living with Dementia: A Phenomenological Study. Act Adapt Aging. 2022 Mar 27;00(00):1–35.

17. Klein CM, Silverman MJ. With love from me to me: Using songwriting to teach coping skills to caregivers of those with Alzheimer’s and other dementias. J Creat Ment Heal. 2012;7(2):153–64.

18. Baker FA, Stretton-Smith P, Clark IN, Tamplin J, Lee Y-EC. A Group Therapeutic Songwriting Intervention for Family Caregivers of People Living With Dementia: A Feasibility Study With Thematic Analysis. Front Med. 2018 May 22;5(May):1–13.

19. Baker FA, Grocke D, Pachana NA. Connecting through music: A study of a spousal caregiver- directed music intervention designed to prolong fulfilling relationships in couples where one person has dementia. Aust J Music Ther. 2012;23.

20. Clark IN, Stretton-Smith PA, Baker FA, Lee YEC, Tamplin J. “It’s Feasible to Write a Song”: A Feasibility Study Examining Group Therapeutic Songwriting for People Living With Dementia and Their Family Caregivers. Front Psychol. 2020;11(August):1–14.

21. Hanser SB, Butterfield-Whitcomb J, Kawata M, Collins BE. Home-based Music Strategies with Individuals who have Dementia and their Family Caregivers. J Music Ther. 2011 Mar 1;48(1):2–27.

22. Denk JG. The Impact of a Music Therapy Support Group on Perceived Stress, Anxiety, and Depression in Long-Term Caregivers: A Pilot Study. Music Ther Perspect. 2022 Jul 6;1–9.

23. Mittelman MS, Papayannopoulou PM. The Unforgettables: A chorus for people with dementia with their family members and friends. Int Psychogeriatrics. 2018;30(6):779–89.

24. Tamplin J, Clark IN, Lee Y-EC, Baker FA. Remini-Sing: A Feasibility Study of Therapeutic Group Singing to Support Relationship Quality and Wellbeing for Community-Dwelling People Living With Dementia and Their Family Caregivers. Front Med. 2018 Aug 31;5(AUG):1–10.
