# Supplementary material for: Music therapy for supporting informal carers of adults with life-threatening illness pre- and post-bereavement; a mixed-methods systematic review
Source: BMC Palliat Care. 2024 Feb 27;23:55. doi: 10.1186/s12904-024-01364-z (PMC10898157; doi:10.1186/s12904-024-01364-z)
Supplement: Supplementary file 4 — Additional file 4. Quasi-Experimental Quality Appraisal Table. Results of methodological assessment of included quasi-experimental articles. [file 12904_2024_1364_MOESM4_ESM.docx]

**Additional file 4**

Additional file 4: Results of methodological assessment of included quasi-experimental articles

|  |  | **Question** | | | | | | | | |  |
| --- | --- | --- | --- | --- | --- | --- | --- | --- | --- | --- | --- |
|  | **Citation** | **1** | **2** | **3** | **4** | **5** | **6** | **7** | **8** | **9** | % |
| 1 | Brotons et al. (2003) (1) | Y | Y* | N/A | N | Y | Y | N/A | Y | N | 55% |
| 2 | Choi et al. (2009) (2) | Y | Y | Y | Y | Y | Y | Y | U | N | 78% |
| 3 | Gallagher et al. (2017) (3) | Y | Y | N/A | N | Y | Y | N/A | Y | N | 55% |
| 4 | Garcia-Valverde et al. (2020) (4) | Y | Y | N/A | N | Y | N | N/A | Y | N | 44% |
| 5 | Holden et al. (2019) (5) | Y | Y | N/A | N | Y | Y | N/A | U | N | 44% |
| 6 | Raglio et al. (2016) (6) | Y | Y | N/A | N | Y | U | N/A | U | N | 33% |
| 7 | Madsø et al (2021) (7) | Y | Y | N/A | N | Y | Y | N/A | Y | Y | 66% |
| 8 | Tamplin et al. (2020) (8) | Y | Y | Y | Y | Y | Y | N | Y | N | 78% |
|  |  | 100% | 100% | 25% | 25% | 100% | 75% | 12% | 62% | 12% |  |
| **Mixed Method Studies Quasi-Experimental Component** | | | | | | | | | | | |
|  | **Citation** | **1** | **2** | **3** | **4** | **5** | **6** | **7** | **8** | **9** | % |
| 1 | Baker et al. (2018) (9) | Y | U | U | Y | Y | Y | Y | Y | N | 66% |
| 2 | Baker et al. (2012) (10) | Y | Y | N/A | N | Y | Y | N/A | U | N | 50% |
| 3 | Clark et al. (2020) (11) | Y | Y | N/A | N | Y | Y | N/A | Y | N | 66% |
| 4 | Hanser et al. (2011) (12) | Y | Y | N/A | N | Y | Y | N/A | U | N | 50% |
| 5 | Tamplin et al. (2018) (13) | Y | Y | N/A | N | Y | Y | N/A | Y | N | 66% |
| 6 | Denk et al (2022) (14) | Y | Y | N/A | N | Y | N | N/A | U | Y | 50% |
| 7 | Mittelman & Papayannopoulou (2018) (15) | Y | Y | N/A | N | Y | Y | N/A | U | N | 43% |
|  |  | 100% | 86% | 0% | 14% | 100% | 86% | 14% | 43% | 14% |  |

Y, Yes; no, No; U, Unclear; N/A, Not Applicable*

1.             Is it clear in the study what is the ‘cause’ and what is the ‘effect’?

2.             Were the participants included in any comparisons similar?

3.             Were the participants included in any comparisons receiving similar treatment/care, other than the exposure or intervention of interest?

4.             Was there a control group?

5.             Were there multiple measurements of the outcome both pre and post the intervention/exposure?

6.             Was follow up complete and if not, were differences between groups in terms of their follow up adequately described and analyzed?

7.             Were the outcomes of participants included in any comparisons measured in the same way?

8.             Were outcomes measured in a reliable way?

9.            Was appropriate statistical analysis used?

*Please note that studies with no control group received a Yes response for question 2 as per JBI guidance

**References**

1. Brotons M, Marti P. Music Therapy with Alzheimer’s Patients and Their Family Caregivers: A Pilot Project. J Music Ther. 2003 Jun 1;40(2):138–50.

2. Choi A-N, Lee MS, Cheong K-J, Lee J-S. Effects of Group Music Intervention on Behavioral and Psychological Symptoms in Patients with Dementia: A Pilot-Controlled Trial. Int J Neurosci. 2009 Jan 7;119(4):471–81.

3. Gallagher LM, Lagman R, Bates D, Edsall M, Eden P, Janaitis J, et al. Perceptions of family members of palliative medicine and hospice patients who experienced music therapy. Support Care Cancer. 2017 Jun 19;25(6):1769–78.

4. García-Valverde E, Badia M, Orgaz MB, Gónzalez-Ingelmo E. The influence of songwriting on quality of life of family caregivers of people with dementia: An exploratory study. Nord J Music Ther. 2020 Jan 1;29(1):4–19.

5. Holden SK, Sheffler J, Stewart R, Thompson S, Persson J, Finseth T, et al. Feasibility of Home-Based Neurologic Music Therapy for Behavioral and Psychological Symptoms of Dementia: A Pilot Study. J Music Ther. 2019 Aug 13;56(3):265–86.

6. Raglio A, Fonte C, Reani P, Varalta V, Bellandi D, Smania N. Active music therapy for persons with dementia and their family caregivers. Int J Geriatr Psychiatry. 2016 Sep;31(9):1085–7.

7. Madsø KG, Molde H, Hynninen KM, Nordhus IH. Observing Music Therapy in Dementia: Repeated Single-case Studies Assessing Well-being and Sociable Interaction. Clin Gerontol. 2022;45(4):968–82.

8. Tamplin J, Morris ME, Marigliani C, Baker FA, Noffs G, Vogel AP. ParkinSong: Outcomes of a 12-Month Controlled Trial of Therapeutic Singing Groups in Parkinson’s Disease. J Parkinsons Dis. 2020 Jul 28;10(3):1217–30.

9. Baker FA, Stretton-Smith P, Clark IN, Tamplin J, Lee Y-EC. A Group Therapeutic Songwriting Intervention for Family Caregivers of People Living With Dementia: A Feasibility Study With Thematic Analysis. Front Med. 2018 May 22;5(May):1–13.

10. Baker FA, Grocke D, Pachana NA. Connecting through music: A study of a spousal caregiver- directed music intervention designed to prolong fulfilling relationships in couples where one person has dementia. Aust J Music Ther. 2012;23.

11. Clark IN, Stretton-Smith PA, Baker FA, Lee YEC, Tamplin J. “It’s Feasible to Write a Song”: A Feasibility Study Examining Group Therapeutic Songwriting for People Living With Dementia and Their Family Caregivers. Front Psychol. 2020;11(August):1–14.

12. Hanser SB, Butterfield-Whitcomb J, Kawata M, Collins BE. Home-based Music Strategies with Individuals who have Dementia and their Family Caregivers. J Music Ther. 2011 Mar 1;48(1):2–27.

13. Tamplin J, Clark IN, Lee Y-EC, Baker FA. Remini-Sing: A Feasibility Study of Therapeutic Group Singing to Support Relationship Quality and Wellbeing for Community-Dwelling People Living With Dementia and Their Family Caregivers. Front Med. 2018 Aug 31;5(AUG):1–10.

14. Denk JG. The Impact of a Music Therapy Support Group on Perceived Stress, Anxiety, and Depression in Long-Term Caregivers: A Pilot Study. Music Ther Perspect. 2022 Jul 6;1–9.

15. Mittelman MS, Papayannopoulou PM. The Unforgettables: A chorus for people with dementia with their family members and friends. Int Psychogeriatrics. 2018;30(6):779–89.
