# Supplementary material for: Music therapy for supporting informal carers of adults with life-threatening illness pre- and post-bereavement; a mixed-methods systematic review
Source: BMC Palliat Care. 2024 Feb 27;23:55. doi: 10.1186/s12904-024-01364-z (PMC10898157; doi:10.1186/s12904-024-01364-z)
Supplement: Supplementary file 5 — Additional file 5. RCT Quality Appraisal Table. Results of methodological assessment of included RCT articles. [file 12904_2024_1364_MOESM5_ESM.docx]

**Additional file 5**

Additional file 5: Results of methodological assessment of included RCT articles

|  |  | **Question** | | | | | | | | | | | | |  |
| --- | --- | --- | --- | --- | --- | --- | --- | --- | --- | --- | --- | --- | --- | --- | --- |
|  | **Citation** | **1** | **2** | **3** | **4** | **5** | **6** | **7** | **8** | **9** | **10** | **11** | **12** | **13** | % |
| 1 | Kim & Dvorak (2018) (1) | Y | N | U | U | Y | U | Y | Y | Y | Y | U | N | Y | 54% |
| 2 | Särkämö et al. (2014) (2) | Y | U | Y | N | N | Y | Y | Y | Y | Y | U | N | Y | 61% |
|  |  | 100% | 0% | 50% | 0% | 50% | 50% | 100% | 100% | 100% | 100% | 0% | 0% | 100% |  |

Y, Yes; no, No; U, Unclear; N/A, Not Applicable

1.             Was true randomization used for assignment of participants to treatment groups?

2.             Was allocation to treatment groups concealed?

3.             Were treatment groups similar at the baseline?

4.             Were participants blind to treatment assignment?

5.             Were those delivering treatment blind to treatment assignment?

6.             Were outcomes assessors blind to treatment assignment?

7.             Were treatment groups treated identically other than the intervention of interest?

8.            Was follow up complete and if not, were differences between groups in terms of their follow up adequately described and analyzed?

9.            Were participants analyzed in the groups to which they were randomized?

10. Were outcomes measured in the same way for treatment groups?

11. Were outcomes measured in a reliable way?

12. Was appropriate statistical analysis used?

13. Was the trial design appropriate, and any deviations from the standard RCT design (individual randomization, parallel groups) accounted for in the conduct and analysis of the trial?

**References**

1. Kim B, Dvorak AL. Music therapy and intimacy behaviors of hospice family caregivers in South Korea: A randomized crossover clinical trial. Nord J Music Ther. 2018 May 27;27(3):218–34.

2. Särkämö T, Tervaniemi M, Laitinen S, Numminen A, Kurki M, Johnson JK, et al. Cognitive, Emotional, and Social Benefits of Regular Musical Activities in Early Dementia: Randomized Controlled Study. Gerontologist. 2014 Aug;54(4):634–50.
