# Supplementary material for: Music therapy for supporting informal carers of adults with life-threatening illness pre- and post-bereavement; a mixed-methods systematic review
Source: BMC Palliat Care. 2024 Feb 27;23:55. doi: 10.1186/s12904-024-01364-z (PMC10898157; doi:10.1186/s12904-024-01364-z)
Supplement: Supplementary file 6 — Additional file 6. Meta-synthesis Tables. Tables show synthesised findings, categories, and extracted study findings. [file 12904_2024_1364_MOESM6_ESM.pdf]

Meta-Synthesis Tables

Table 1: Synthesized finding 1: Social connectedness and social support

Findings (n=37)	Categories (n=2)	Synthesised Finding
(A3) Sense of connectedness and reduction in loneliness [U]<br>(A4) Sense of group cohesion and agreement when agreeing on the song's direction [U]<br>(A6) Despite 'miles' between each participants' experience of caring, participants identified broader commonalities and gained a sense of 'belonging' to the same group. [U]<br>(A8) Song writing group met an important need not met through other support groups attended in the past [U]<br>(E6) "Participants also focused on Therapeutic Song Writing (TSW) as a positive social opportunity that was different to other group experiences. […] "the music allowed you to sort of interchange with other people" [U]<br>(E7) Dyads identified the goal and process of TSW and use of music as important factors prompting interaction, collaboration, and connection." [U]<br>(E21) "one dyad appreciated recommendations from the music therapist to arrive half an hour early to allow time for socialization." [U]<br>(I1) Participants found that the process of composing a collaborative song led to a climate of trust and affection among them, in which a strong group identity was generated [U]<br>(I2) In addition, some participants emphasized that this sense of connection contributed to diminishing their perception of loneliness and isolation, which was also reflected in the lyrics of the songs [U]<br>(K1) The participants described feelings of belonging and acceptance when taking part in the singing sessions. [C]<br>(K2) They spoke ….of community that making music as a group created [C]<br>(K3) For five participants, their sense of belonging appeared to be rooted in their shared experience of having, or caring for a family-member with, early-stage dementia [U]	1.1 Social connectedness – social opportunity and reduction in loneliness<br><br>Findings (n=16)	Group therapeutic musical/singing interventions (both pre- and post-bereavement) offered positive social opportunities which helped to reduce loneliness. Commonalities in experiences and connection with other group members helped to foster solidarity, empathy, and the sense of enjoying a "safe space" where other informal carers could explore and share their views and experiences. There was a sense that the participatory nature of the group supported a feeling of belonging, and that the group environment itself acted as a conduit for carers to express their

(K13) They also discussed how unifying and inclusive the experience of singing together was [U]<br><br>(T14) Provided a musical framework where participants could feel togetherness and be independent at the same time. [U]<br><br>(X2) While Joan felt that she could not confirm whether participation in the choirs had a sustained impact on her mood, her son (Mark) challenged her opinion, and highlighted how subtle changes may be difficult for one to notice within themselves [U]<br><br>(X11) Social connection was described as an important need of participants with dementia and care-partners, and a reason they joined the choir [U]		feelings, elevating the opportunity for externalisation of feelings beyond what could be achieved in a one-to-one or dyad-music therapist setting.
(A1) Songwriting group was unique and helpful as it offered the opportunity to talk about stages of caregiving and aspects of the caregiving experience [U]<br><br>(F3) Therapeutic singing group provided a supportive environment where people felt comfortable [U]<br><br>(F4) An environment of acceptance enabled PwD who had previously sang in choirs to continue singing [U]<br><br>(F5) Empathic friends fostered for family group carers [U]<br><br>(G7) TSW group processes fostered empathic relationships and experiences of inclusion [U]<br><br>(I3) Participants stated that the creation of the song allowed them to share common feelings and needs, as caregivers of people with dementia. [U]<br><br>(I4) Throughout the therapeutic process, they could listen to each other, be listened to, and feel that they shared similar realities. This created meeting spaces, feelings of identification, empathy, and mutual support [U]<br><br>(I5) Most of the participants agreed that group support was the driving force that boosted the song's composition. Some caregivers expressed that they would not have felt able to complete the creation process on their own, but they could do this in a group, as the link generated provided them with security and trust [U]<br><br>(K4) The carers spoke of the comradery, support, and solidarity that this fostered among them [U]<br><br>(K21) FC3 recalled how the flexible and supportive environment encouraged participants to contribute [U]<br><br>(T1) Category 1: Group singing experiences/the Singing Well context: Fostered feelings of connection, awareness, and support. During the sessions or during singing experiences, participants felt connected to/aware of: themselves, their emotions, their own/others' voices, their breath, their bodies, other group	1.2 Safe, non-judgemental, supportive space to share feelings and receive social support while experiencing a sense of belonging<br><br>Findings (n=21)	

members, the music, their deceased loved ones, memories, the here and now, the future. They felt supported by each other and/or by the music. [U]<br><br>(T4) Provided a comfortable, non-judgmental, accepting, supportive, and safe space. Participants could freely express and/or experience their feelings; explore their voices/sing without feeling judged; accepting and sensitive to each other's needs. [U]<br><br>(U1) During the focus group discussion, group members noted the benefits of meeting and receiving support from other group members. Forms of support included recommendations of care providers, such as physicians and therapists specializing in their loved one's diagnosis, and perspective from participants who have been caregivers for longer periods of time [U]<br><br>(U2) Regarding the impact of the support group on anxiety, one participant stated no increase [U]<br><br>(U4) One participant noted a decrease in feelings of depression over the final month of the intervention, crediting the support group [C]<br><br>(X12) Although Eileen did not articulate precisely why she needed companionship from others, this was echoed by other participants who felt that they had become socially isolated due to friends becoming distant following the diagnosis of dementia [U]<br><br>(X13) Participants described experiencing a sense of deep sense belonging in the Remini-Sing choirs. Several participants compared the group to a community or club, or feeling as if they were 'at home' [U]<br><br>(X14) Eileen (C) speculated that the participatory nature of the group created a more mutual sense of belonging than other social groups: [U]<br><br>(X15) Some participants described feeling a sense of safety or security within in the group that was enabled by their connections with one another [U]<br><br>(X16) Participants also reported feeling supported by people who had similar experiences: [U]<br><br>(X17) For participants in this study, the 'social support' seemed to relate to an unspoken understanding, and a 'kindness' (Kate, C) that members could afford each other due to their shared understanding of what it's like navigating a dementia diagnosis. [C]		

Table 2: Synthesized finding 2: Music as an emotional/communication channel and spiritual bridge

**Findings (n=18)**	**Categories (n=2)**	**Synthesised Finding**
(D1) Music brought up a lot of emotion during sessions [C]<br><br>(I11) Besides, some participants indicated that they felt an emotional response when listening to the songs again, and that it was important for them to be able to relive and remember this experience [U]<br><br>(I14) Participants stated that the creation of a collaborative song stimulates the exploration and expression of emotions, sensations, and thoughts. [U]<br><br>(I15) In addition, they emphasized that they had not only been able to free and externalize their feelings, but also to channel them [U]<br><br>(P2) Music-related memories elicited participants' sadness, pleasure, or both. [U]<br><br>(T2) Evoked a range of emotions, expressed and experienced in various ways. During and/or after expressing emotions, participants felt uplifted, drained, peaceful, calm, sense of freedom/release, compassion, detached. [U]<br><br>(T5) Allowed participants to express themselves in a way that was different than talking. Sometimes words could not adequately express feelings or words alone were insufficient. [U]<br><br>(T10) Evoked meaningful memories, emotional release; a means of escape. Through lyrics and/or music. Participants sometimes caught off guard by their responses. [U]	2.1 Musical catharsis – music therapy enabled caregivers to release repressed emotions<br><br>Findings (n=8)	Music therapy had a cathartic effect, eliciting and providing a safe channel for participants to experience, process and communicate a range of grief-related emotions, and was seen as a spiritual bridge to something beyond the bereavement experience, providing meaning and hope of something more beyond death, as well as a sense of connection to the deceased. It was considered a conduit, often particularly important/powerful at end of life, for enabling caregivers to transcend their bereavement experience and find peace.
(L8) Patients and caregivers commonly find an enhanced sense of peace as a result of the supportive context of music therapy sessions [U]<br><br>(M1) The power of music to lift, transport, and engage attention and response, as well as to help ''put on a track,'' was described by the caregivers as being an important aspect of the music in sessions. The word	2.2 Music as a bridge/spirituality /finding meaning/hope and transcendence	

conduit, that is, a channel or vehicle, describes the views of several of the participants and is an aspect of music that embodies the process of transcendence. [U]<br><br>(N12) The caregivers made references to their own personal connections with themselves within the content of interviews. These moments of self-acknowledgement and self-affirmation were important to the caregivers, as they were able to perceive their own inner strengths and attributes and gain an enhanced sense of meaning as they reflected on their roles and the larger themes in their own lives [U]<br><br>(N14) The third prevalent connectedness theme was association with the "beyond"… References were made to music and its potential to act as a medium through and by which a relationship to this realm could be made. [U]<br><br>(N23) In other cases the caregivers referred to thoughts pertaining to the beyond in the context of hope: [U]<br><br>(P3) Participants used music to connect with the deceased [U]<br><br>(P5) Musical efforts of the deceased inspire others [U]<br><br>(S2) The following finding relates both to patients and carers: "While experiencing the intervention patients described experiencing pleasant images and visualizations like "being in other spheres", "swimming on waves" or hearing nice voices. Most often these images were connected to former experiences of relaxation that patients had encountered in the past or to symbolic situations associated with feelings of calmness and inner peace." [U]<br><br>(S4) "Furthermore, it had calming and relaxing effect on them and it was associated with other types of experiences such as autogenic training or being at the beach." [U]<br><br>(T13) Category 4: Improvised vocal experiences (chanting, toning, humming, creating melodies, harmonies): For most, improvised vocal experiences felt supportive, evocative, and/or liberating. A sense of freedom/ release; evoked emotions, imagery, physical sensations of floating/flying. [U]	Findings (n=10)	

Table 3: Synthesized finding 3: Positive reminiscence of pre-illness identifies/relationships and finding balance between grief and life going forward

**Findings (n=49)**	**Categories (n=3)**	**Synthesised Finding**
(B2) Claudia also found pleasure in witnessing Charles' responses [U]	3.1 Positive reminiscence –	Music therapy deepened relationships, and

(B8) For Jim and Betty, the music intervention facilitated reminiscence and a form of interaction that was typical of their relationship in their earlier years together as a couple — such as dancing [U]<br><br>(B10) Iris and Bill experienced shared humour: [U]<br><br>(D2) Music therapy created opportunities for reflection on personal narratives and pivotal life moments with their loved one, or anticipatory grief. [C]<br><br>(E24) “others highlighted the value of reminiscence focused TSW.” [U]<br><br>(I12) Participants described GTSW as a space that allowed them to distance themselves from their facet of caregivers and find themselves, with their feelings and emotions [U]<br><br>(K19) The carers also reported how seeing their loved ones enjoying themselves had a positive impact on their own mood: [U]<br><br>(K23) The carers expressed happiness at observing their family members being able to offer their talents and contribute to the sessions: [U]<br><br>(K31) Participants saw the singing sessions as good opportunities to spend meaningful one-to one time with their family member, where their roles were incidental and could be forgotten: [U]<br><br>(N8) Coming back to life and having a renewal of self were striking themes that often signified their being able to partake in interactions and being able to see glimpses of their personalities, attributes, and strengths once again. [U]<br><br>(N9) Across interviews, caregivers commented on their observations of seeing loved ones display personal traits in music therapy sessions that had otherwise been absent during this stage of illness [U]<br><br>(N20) At times, the caregivers ruminated on their childhoods and other memorable life events [U]<br><br>(X6) Participants described how the repertoire sung in the choir triggered reminiscence for participants with dementia [U]	reconnecting with self/significant other (i.e. pre-illness/bereavement identities and relationships)<br><br>Findings (n=13)	communication between the patient and carer. Music therapy was considered to be an activity which carers and patients could share, and which allowed them to interact in ways that resembled previous relationship dynamics, prior to the advent of the carer-patient relationship. Carers were able to enjoy the patient experiencing the music, which could lead to the carer experiencing the patient as having a renewal of their formal self, and they sometimes engaged in reflection, reminiscing, and life review together. This could also support the carer in enjoying positive memories of the patient. Music therapy activities were sometimes perceived to increase the carer's sense of connection to the patient and reignite/strengthen their relationship and sense of
(B5) Four of the spouses (Jim, Iris, Geoff and Ian) indicated that the music intervention enhanced the quality of time spent together and that listening to music, dancing, or talking shaped these moments [U]<br><br>(B6) Iris stated that music provided space for them to share — it was a “common ground”: [U]<br><br>(B7) The music intervention affected the quality of Geoff and Helen's relationship because it directed them to stop and take time to share time together: [U]	3.2 Music therapy as a shared activity which improved caregiver/patient relationships and aided relationship	

(B9) Three caregivers spoke of a strengthened reciprocity in their relationship. Iris described the quality time spent together as rekindling the feelings of intimacy in her relationship through the sharing and purposeful act of being in the moment with her partner [U]<br><br>(D3) Witnessing of their loved ones' emotional and narrative expressions [C]<br><br>(D4) Unexpected opportunities for life review through music [U]<br><br>(E2) "Importantly, the vast majority of dyads commented on appreciating being able to attend together and highlighted the value of TSW as a new, creative, and stimulating shared experience. [U]<br><br>(E3) "half of the dyads interviewed also commented that the experience of participating together felt "natural" (Michelle/Ann) and "normal" (Ellen/Tom), explaining that they had always had a "very strong bond" (Carlo/Isabella) and "done everything together" (Jan/John). In this sense, the TSW program appeared to largely support, rather than change, existing relationship quality. [U]<br><br>(E12) "participants recognized the role of family caregivers in supporting and "guiding" their family member with dementia. Participants commented that having both members of the dyad in the group was the "best way to do it" (Ian∗)." [U]<br><br>(G2) TSW was something to look forward to and do together [U]<br><br>(H3) Singing improved the couple's relationship [C]<br><br>(K15) The programme stimulated social interactions beyond the sessions themselves: [U]<br><br>(M2) Caregivers also described this potential of music as it attributed to opportunities for meaningful moments of communication [U]<br><br>(M8) The caregivers referred to the tendency for music to inspire, touch, and bring the feelings of love…Caregivers had lasting impressions of the love-filled verbal, nonverbal, and musical communications experienced in sessions [U]<br><br>(N4) Empathic joy was a common trend across interviews and was heard in comments that reflected this thought: [U]<br><br>(N11) Another caregiver explained these thoughts regarding "coming back" in music therapy in such a subtle and gentle way as to allow for final communications to occur [U]<br><br>(N13) Connectedness with others was a predominant theme in this study, as caregivers unanimously made references to their thoughts and feelings regarding revived interrelatedness. Most commonly these	completion at end of life<br><br>Findings (n=25)	intimacy. reciprocity in the patient-carer relationship was perceived to be strengthened.<br><br>Collectively, reconnecting with their pre-illness/caregiver identities and deepened relationships with their significant other aided relationship completion and preparedness for death, which in turn helped them balance their grief with their renewed interest in life going forward.

statements emerged in descriptions regarding connection and communication with the patients in, around, and following the music in sessions [U]<br><br>(N18) The caregivers were drawn, on their own initiative, to recollect life events and reminisce about times with and apart from the patient. Their reflections were commonly inspired by the memories evoked through the songs in sessions and the broader connections these songs had to their lives [U]<br><br>(P6) Music therapy was an opportunity to share important messages, enabling unspoken closeness and comfort in life and death [U]<br><br>(S3) "A shared positive experience of the music therapy seemed to facilitate a connection between the patients and the family members. During some interviews, family members of patients were present and thus asked to share their experiences of the intervention. The family members described their own experiences of the music. They were very enthusiastic about the therapy and found it comforting." [C]<br><br>(S7) "One relative mentioned that the experience of the treatment session could help her to turn her sorrow about the disease into gratitude for being able to spend time with her family member." [U]<br><br>(X18) Participating together was beneficial for practical reasons. [U]<br><br>(X19) Nancy (C) and Colin (D) reported that they enjoyed attending together because it reflected their approach to co-participation throughout their lives. [U]<br><br>(X20) Eileen (C) felt that participating together with her husband was something they prioritized, and that the choir provided an enjoyable platform for the two of them to do so: [U]<br><br>(X21) Several participants reported feeling that attending with their family member(s) gave them something to bond over at home. [U]		
(A7) Song writing experience helped carers gain a sense of inner strength [U]<br><br>(G3) The experience helped to break a 'barrier' to music engagement and gave them a new 'appreciation' for music, as well as ideas for how to use music and song writing beyond sessions. [U]<br><br>(G5) TSW reignited and connected them with their skills and interests in poetry [U]<br><br>(N5) The caregivers conveyed that feelings of joy seemed to assist them in preparing for the eventual death of their loved ones in that they knew the patients had moments of contentment and peace before dying [C]	3.3 Preparedness for death/balancing grief and life going forward<br><br>Findings (n=11)	

(N7) The feelings of empowerment they attained in, and as a result of, music therapy helped reduce their own sense of helplessness and facilitated their preparedness to let the patient "go in peace," [C]<br><br>(N24) Throughout the study, reflections on hope were commonly directed primarily toward their own personal lives [U]<br><br>(P4) Music often still improves mood or helped participants to get on with life [U]<br><br>(T9) Category 3: Songs (precomposed/original): Reflected, validated, re-conceptualised participants' feelings of grief and loss. Through lyrics and/or music. [U]<br><br>(T16) Motivated participants to make changes, explore new/revive previous interests in their daily lives. Participants became more musically active and/or initiated other activities/practices outside of the Singing Well context. [U]<br><br>(T17) Helped participants to move forward in their lives. The experience of being in the Singing Well group enabled participants to actively (re) engage in various aspects of their lives and/or adapt to current realities. [U]<br><br>(X10) For some care-partners, the choirs provided a chance to connect to different parts of their identity [U]		

Table 4: Synthesized finding 4: Positive mental wellbeing: sense of meaning and purpose in life

**Findings (n=37)**	**Categories (n=2)**	**Synthesised Finding**
(B1) All five caregivers reported that the music had an affect on their own wellbeing, particularly that engaging in music had a relaxing effect: [U]<br><br>(B3) Jim indicated that when music relaxed him, he felt more invigorated: [U]<br><br>(E1) Theme: Dyads' Experiences of Group Therapeutic Songwriting "TSW was overwhelmingly recognized as a positive, worthwhile, and enjoyable experience that participants were willing to commit time to." [U]<br><br>(E4) "Despite some family caregivers initially being incentivized to attend for their family member with dementia, all participants recognized personal benefits for both members of the dyad, including joy, motivation, pride, and achievement." [C]	4.1 Positive mental wellbeing<br><br>Findings (n=30)	Intervention offered and facilitated enjoyment of aesthetic qualities of music. Intervention offered a creative outlet which allowed participants to express meaningful inner emotions through creation of music

(E5) "([…] one family caregiver) also recognized a positive impact on mood. Further, caregivers found meaning in witnessing their family member and others enjoying, gaining from, and contributing to the TSW process." [U]<br><br>(G1) Group TSW was an overwhelmingly positive, fun and enjoyable experience that surpassed expectations [U]<br><br>(I13) Some participants emphasized that disconnecting from the disease is a "need" for them, which provides them with "calm" and "relief." [C]<br><br>(I18) Many participants noted that, after the sessions, they were happier and that, throughout the therapeutic process, they perceived improved mood [U]<br><br>(I19) Other caregivers noted that being part of the GTSW favored positive thoughts and greater optimism in them [U]<br><br>(K16) The participants recurrently reported that the singing sessions made them feel good. The sessions were associated with happiness, joy, and a sense of being uplifted: [U]<br><br>(K17) FC3 described how she felt rejuvenated and relaxed after the sessions: [U]<br><br>(K22) Six participants noted that as a group, everyone was eager and able to participate, and excited to learn something new: [U]<br><br>(L1) Patients and caregivers tend to find joy and pleasure in hearing and participating in songs, singing, and reminiscing [U]<br><br>(L7) Caregivers and patients alike are soothed and refreshed by the aesthetic beauty of music; and caregivers convey happiness in knowing they played a role in bringing this to their loved ones [U]<br><br>(N2) Music had a direct effect on each of the caregivers as they witnessed and participated in the music therapy sessions. They articulated wanting to be near the music and found the sounds to bring pleasure. [U]<br><br>(M5) Caregivers sometimes referred to its (sic music's) stimulating and energizing nature, for example, during periods of restored awareness and communication [U]<br><br>(M6) Likewise, they referred to this aspect of music that reaches and helps soothe and relax during times of distress [U]		and/or lyrics. Intervention offered routes of self-expression or connection, such as dance and moving to the music. Music could be used as a tool to regulate carers' and patients' moods - it could help to relax or to invigorate and was considered to have a positive impact on carer mood. Some interventions encouraged use of music in daily life or otherwise when the intervention facilitator was not present, and it was considered to be of use as a caregiving tool to manage patient symptoms, including for gently bringing them back to life near EOL.

(N1) Although some caregivers did not use the word joy, the term portrays the fundamental nature of the mood that was conveyed by all caregivers and the general happiness they felt as a result of the positive effects of music therapy [U]<br>(N3) They also referred to the internal pleasure of the presence of music and the experience of singing. This joy exemplified their personal responses of happiness and contentment resulting from the music therapy sessions [U]<br>(N22) Several caregivers described the sense of hope experienced as a result of the music therapy, as the “transporting” effects of music were comforting and “rejuvenating.” [U]<br>(S5) “One family member even reported that he felt a relief of muscle tension and pain due to the treatment.” [C]<br>(T3) Allowed participants to enjoy themselves and feel good. Participants felt able/gave themselves permission to experience pleasure within the Singing Well context and/or during the singing experiences. [U]<br>(U3) The other participant noted an increase in anxiety midway through the study [C]<br>(U5) During the focus group discussion, participants reflected on the activities completed in each session and stated how these activities transferred to their lives outside the support group sessions. [U]<br>(U6) Finally, when speaking to the impact of the support group on perceived stress [U]<br>(X1) Participants described feeling a positive boost while participating in the Remini-Sing choirs. They often used language such as ‘fun,’ ‘enjoy’ or ‘joy’ to describe how they felt during the act of singing together with others, as well as participation in the groups more broadly [U]<br>(X3) A more distinctive impact on mood was described by other participants. Kate (C) reported feeling calm following the groups [U]<br>(X9) Some participants described enjoying the fact that they were able to learn about different aspects of music, be it songs, musical skills, or facts about the songs themselves [U]<br>(X22) Some participants described a sense of nervousness at starting something new. [U]<br>(X28) In addition to the comfort that a regular routine can provide, consistent attendance provided regular opportunities for positive moments, which were important in the context of challenges that participants were facing in their lives [U]		

(H2) Singing assisted the spouse in her role as primary carer [C]<br><br>(L2) Caregivers often feel empowered by knowing that they have played a role in bringing this joy to their loved ones [U]<br><br>(L3) Caregivers benefit from exposure to, and participation in, such methods that provide comfort-giving measures to their loved ones [U]<br><br>(L4) Likewise, caregivers describe the cyclical nature of giving and receiving in life, and the comfort they find in being able to "give back comfort and pleasure [to their loved one] through music." [U]<br><br>(L5) They note feelings of gratitude toward music therapy because it can help mitigate disease-related distress. Caregivers tend to find relief in knowing that through music therapy they can play a role in symptom management. [U]<br><br>(L6) Caregivers often report feeling satisfaction at having contributed to attaining moments in which loved ones were able to access waning personal attributes and find enhanced humanness, identity, and dignity. Thus, due to music therapy sessions caregivers are empowered by the knowledge that they helped loved ones find greater inner peace and resolution during their approach to death [U]<br><br>(N6) The caregivers each described the relief, comfort, and fulfilment that they felt in being able to bring pleasure, as well as allay some of their loved one's distress [U]	4.2 Music therapy as a caregiving tool: provided caregiver with a sense of meaning and purpose in life<br><br>Findings (n=7)	

Table 5: Synthesized finding 5: Contextual and Implementation Factors

**Findings (n=50)**	**Categories (n=2)**	**Synthesised Finding**
(C3) Theme 3: the song product is meaningful [U]<br><br>(E11) "Participants also identified aspects of the approach and facilitation that were important in supporting and including all participants, especially those with more advanced dementia or who were nonverbal or used assistive communication devices. These included choosing the "right songs" (Ian∗), […] and integrating singing, instrument playing, music listening, and visual prompts into the TSW program." [U]<br><br>(E13) "One family caregiver commented that her husband experienced difficulties adjusting to the group setting, while another family caregiver noticed that his wife and some other participants with dementia had difficulty maintaining focus during TSW. Lyric writing was recognized as a valuable and gratifying experience for the majority. However, one participant who was in the more advanced stages of dementia	5.1 A flexible, tailored approach is required<br>Findings (n=39)	Flexible, tailored approaches helped to maximise participant comfort and engagement. Carer needs could differ according to the patient's condition. Factors requiring

and nonverbal was unable to contribute to lyric writing. Similarly, while the recording session was described by many as the “climax” (Ellen/Tom) of the program due to it being a new and intriguing experience, one family caregiver felt that the “new equipment [and] close proximity to people” (Carlo) presented challenges for his wife.” [U]<br><br>(E14) “However, despite these challenges, all dyads recognized TSW as worthwhile.” [U]<br><br>(E15) Theme: Therapeutic Songwriting Intervention Design and Delivery “generally, participants felt the groups of 2–3 dyads were enough to trigger thoughts and exchange ideas, while still being able to reach a consensus and value individual contributions. Further, participants speculated that the small group context may be easier than TSW with larger groups or individual dyads.” [U]<br><br>(E16) “Six weekly sessions were generally perceived as an appropriate amount of time for composing 2–3 songs using song parody and/or song collage techniques. However, some suggested that the program could have been extended.” [U]<br><br>(E17) Others stated […] that an ongoing program would “help dramatically” (Jan∗), even on a less regular basis. [U]<br><br>(E18) “One participant expressed that TSW sessions could have been more frequent, such as “every two or three days” (George∗), although acknowledged this may be difficult for others.” [U]<br><br>(E19) “One-hour sessions were experienced as “just right” (Neil/Mei).” [C]<br><br>(E20) “Sessions were long enough to develop and work on new ideas, but not too long as to be difficult for participants to sustain focus” [U]<br><br>(E22) “One dyad also suggested that sessions in the morning rather than late afternoon may be “better” (Elizabeth∗) in residential care as people with dementia might experience less confusion and find it easier to participate at that time.” [C]<br><br>(E23) “Regarding TSW methods and techniques, the majority of participants recognized song parody as an appropriate level of challenge and considered original music creation as “too much to ask” (Ian∗). [U]<br><br>(E25) “Finally, the majority of dyads described appreciating having an “end product” in the form of a recording, which enabled them to listen back to the final song creations, gain a sense of completion, and recognize the achievements of the group.” [U]<br><br>(F1) Participants thought the size of the group worked well (n=18) [U]		consideration include intervention timing and duration, and group size. The goal of interventions was influential, for example, where social support was an objective, groups should be large enough to facilitate this and to be sustainable, but small enough to build relationships. Session regularity was valued. Carers of people with dementia who attended in dyads could prefer short sessions or certain times of the day.<br><br>Participants had differing levels of musical experience, while loved ones experienced different stages of cognitive decline. It was important that activities were inclusive and

(I6) Composing a song was a challenge for the caregivers, especially in the early stages where they were unaware of how the process would be articulated [U]<br><br>(I7) Participants stated that, as they created the song and overcame the challenge, they felt increasingly satisfied and self-confident [U]<br><br>(I9) According to the participants' narratives, at the beginning of the intervention, their level of engagement was low due to the complexity of the task they faced. However, as the process progressed, and the song materialized, their motivation increased, and their expectations were overcome [U]<br><br>(K5) The participants observed that the collaborative and inclusive nature of the singing sessions and facilitator's flexible approach encouraged active engagement: [U](<br><br>(K7) …and that the small group size and similar cognitive capacities encouraged participation: [U]<br><br>(K24) The musical content of the sessions was driven by the participants, and this level of control was valued [U]<br><br>(K29) Both FC1 and FC2 appreciated the fact that they could attend the music program with their husbands, and that it took place in the morning, helping to alleviate these issues [C]<br><br>(M7) They (sic the carers) spoke about the use of songs and live music in sessions versus recorded music. In particular, they commented on the beneficial presence of live music, the use of familiar songs, and the involvement of the voice [U]<br><br>(T11) Choosing songs was important. Participants enjoyed choosing songs. Found meaning in familiar and unfamiliar songs. Supported others by supporting their song choices. [U]<br><br>(X4) Some care-partners felt that the group provided opportunities for their loved ones to practice cognitive tasks. [U]<br><br>(X5) Eileen (C) felt that the format of encouraging participant choice helped to encourage her husband to participate [U]<br><br>(X8) Participants described how the group afforded them opportunities to learn new skills or practice existing ones. [C]<br><br>(X23) Participants described feeling compelled to participate due to the low recruitment numbers in the early stages of the choirs [U]		delivered at an appropriate level. Participant choice over songs and activities supported their engagement, and where a song was produced by the group, this was meaningful.<br><br>The skills and characteristics of the music therapist themselves also influenced participants' experiences. Empathy and a commitment to the therapeutic relationship were crucial. It was important that facilitators maintain an enthusiastic and knowledgeable presence which encouraged participation, but which also allowed the participants to take the lead. Participants appreciated when

(X24) Several logistical aspects of the choir design were felt to be accessible; these included location and accessibility of the venue (particularly proximity to home), timing, and group size. Timing of sessions was the most prevalent logistical factor described by participants [U]<br><br>(X26) Participants felt that the 90-minute length of the sessions was acceptable: [C]<br><br>(X27) The regularity of sessions was highlighted as an important aspect of the choir; the group discussed that weekly sessions were useful in creating a habit of attending [U]<br><br>(X29) As discussed in the previous theme, the size of the groups was highlighted as an element of the choirs that impacted participation. Participants additionally acknowledged, however, that larger groups would support sustainability of the choir: [U]<br><br>(X33) Although singing was described as something that participants with dementia could engage in, regardless of their cognitive challenges, participants felt that the relaxed musical expectations helped to create an environment where all abilities were welcomed [U]<br><br>(X34) Patrick (C) described the importance of everyone being afforded an opportunity to select repertoire: [U]<br><br>(X35) Mark echoed this sentiment, and felt that the way that activities (such as warm ups and song choice) were designed to encourage active participation was important [U]<br><br>(X36) Although relaxed musical standards were appreciated, Nancy (C) described a enjoying a good balance between repertoire that was familiar and easy, and that which was more challenging: [U]<br><br>(X37) Uncertainty about the groups ending was frustrating to participants. [U]<br><br>(X38) Membership and recruitment were discussed as a key factor impacting the long-term security of the group. Participants expressed concern that the low membership numbers of their respective groups made them vulnerable to collapse, both due to lack of attendance, and the cost-effectiveness of running small groups [U]<br><br>(X39) The lack of success recruiting new members, both during the initial study period, and the extended post-study time was cited as a point of frustration. [U]<br><br>(X40) On several occasions, participants strategized about how they could support the groups to continue themselves. [U]		facilitators were organised and instructions clear.

(E8) Theme: Enablers, Barriers, and Types of Participation in Therapeutic Songwriting "All dyads described how facilitation methods supported the achievability and success of TSW, including for people who had never written a song before." [U]<br><br>(E9) "Dyads were surprised by "the ease of the whole process" (Carlo∗), largely attributing this to supportive aspects of facilitation and therapeutic "leadership," including the therapist prompting and encouraging participants to "have a go" (Michelle∗) and suggesting and organizing the groups' thoughts and ideas." [U]<br><br>(F2) Participants commented on facilitator enthusiasm, organisation, information emails and take-home practice tracks [U]<br><br>(O1) The music therapist displays calming and compassionate personal attributes: [C]<br><br>(O2) The music therapist uses subtleness of approach and has specialized person-centered therapeutic skills [C]<br><br>(O3) The music therapist maintains a supportive and interactive role [C]<br><br>(O4) The music therapist establishes and maintains an ongoing therapeutic relationship: [C]<br><br>(T6) Worked well because of personal and professional qualities of the music therapist. Participants felt the music therapist (and intern) had unique qualities, skills, and abilities including flexibility, empathy, musical/creative talent, sensitivity, compassion. They were approachable, dependable, supportive, leaders [U]<br><br>(X30) Participants felt the facilitator played a role in helping participants feel comfortable and actively engage in sessions. While facilitators were generally described positively, Kate (C) highlighted some experiences where facilitation was lacking, which resulted in her and others feeling confused and stressed: [U]<br><br>(X31) Colin (D) emphasized the importance of facilitator training, noting that he felt facilitators should have specific knowledge about the people they are working with: [U]<br><br>(X32) Mark (C) and Joan (D) felt that at times, the role of the facilitator appeared subtle, and that the true importance of the facilitator was only revealed when there were issues in the group (where participants needed particular support): [U]	5.2 Facilitator/music therapy skills<br><br>Findings (n=11)	

Key

[U] = Unequivocal finding   [C] = Credible finding

Alphanumeric codes: Study ID code letter

Study ID letter	Author (Year)	Reference
A	Baker et al. (2018)	(1)
B	Baker et al. (2012)	(2)
C	Baker & Yeates (2018)	(3)
D	Black et al. (2020)	(4)
E	Clark et al. (2020)	(5)
F	Clark et al. (2018)	(6)
G	Clark et al. (2021)	(7)
H	Dassa et al. (2020)	(8)
I	García-Valverde et al. (2022)	(9)
J*	Hanser et al. (2011)*	(10)
K	Lee et al. (2022)	(11)
L	Magill (2009c)	(12)
M	Magill (2009a)	(13)
N	Magill (2009b)	(14)
O	Magill (2011)	(15)
P	O'Callaghan et al. (2013)	(16)
Q	Potvin et al. (2018)	(17)
R	Tamplin et al. (2018)	(18)
S	Teut et al. (2014)	(19)

T	Young & Pringle (2018)	(20)
U	Denk et al. (2022)	(21)
V	Klein & Silverman (2012)	(22)
W†	Mittelman & Papayannopoulou (2018)†	(23)
X	Thompson et al(2022)	(24)

*Removed from meta-synthesis as available qualitative data were anecdotal

†Removed from meta-synthesis as eligible participant data could not be separated

1. Baker FA, Stretton-Smith P, Clark IN, Tamplin J, Lee Y-EC. A Group Therapeutic Songwriting Intervention for Family Caregivers of People Living With Dementia: A Feasibility Study With Thematic Analysis. Front Med. 2018 May 22;5(May):1–13.
2. Baker FA, Grocke D, Pachana NA. Connecting through music: A study of a spousal caregiver- directed music intervention designed to prolong fulfilling relationships in couples where one person has dementia. Aust J Music Ther. 2012;23.
3. Baker FA, Yeates S. Carers’ experiences of group therapeutic songwriting: An interpretive phenomenological analysis. Br J Music Ther. 2018;32(1):8–17.
4. Black S, Bartel L, Rodin G. Exit Music: The Experience of Music Therapy within Medical Assistance in Dying. Healthcare. 2020 Sep 10;8(3):331.
5. Clark IN, Stretton-Smith PA, Baker FA, Lee YEC, Tamplin J. “It’s Feasible to Write a Song”: A Feasibility Study Examining Group Therapeutic Songwriting for People Living With Dementia and Their Family Caregivers. Front Psychol. 2020;11(August):1–14.
6. Clark IN, Tamplin JD, Baker FA. Community-Dwelling People Living With Dementia and Their Family Caregivers Experience Enhanced Relationships and Feelings of Well-Being Following Therapeutic Group Singing: A Qualitative Thematic Analysis. Front Psychol. 2018 Jul 30;9(July):1–13.
7. Clark IN, Baker FA, Tamplin J, Lee Y-EC, Cotton A, Stretton-Smith PA. “Doing Things Together Is What It’s About”: An Interpretative Phenomenological Analysis of the Experience of Group Therapeutic Songwriting From the Perspectives of People With Dementia and Their Family Caregivers. Front Psychol. 2021 Mar 31;12(March):1–15.
8. Dassa A, Rosenbach M, Gilboa A. Towards sustainable implementation of music in daily care of people with dementia and their spouses. Arts Psychother. 2020 Nov;71(September):101713.

9. García-Valverde E, Badia Corbella M, Orgaz Baz MB. Experiences of group therapeutic songwriting of family caregivers of people with dementia. Psychol Music. 2022;50(2):530–47.

10. Hanser SB, Butterfield-Whitcomb J, Kawata M, Collins BE. Home-based Music Strategies with Individuals who have Dementia and their Family Caregivers. J Music Ther. 2011 Mar 1;48(1):2–27.

11. Lee S, O'Neill D, Moss H. Promoting well-being among people with early-stage dementia and their family carers through community-based group singing: a phenomenological study. Arts Health. 2022 Jan 2;14(1):85–101.

12. Magill L. Caregiver Empowerment and Music Therapy: Through the Eyes of Bereaved Caregivers of Advanced Cancer Patients. J Palliat Care. 2009 Mar 19;25(1):68–75.

13. Magill L. The Meaning of the Music: The Role of Music in Palliative Care Music Therapy as Perceived by Bereaved Caregivers of Advanced Cancer Patients. Am J Hosp Palliat Med. 2009 Feb 8;26(1):33–9.

14. Magill L. The spiritual meaning of pre-loss music therapy to bereaved caregivers of advanced cancer patients. Palliat Support Care. 2009;7(1):97–108.

15. Magill L. Bereaved Family Caregivers' Reflections on the Role of the Music Therapist. Music Med. 2011 Jan 1;3(1):56–63.

16. O'Callaghan CC, McDermott F, Hudson P, Zalcberg JR. Sound Continuing Bonds with the Deceased: The Relevance of Music, Including Preloss Music Therapy, for Eight Bereaved Caregivers. Death Stud. 2013 Feb;37(2):101–25.

17. Potvin N, Bradt J, Ghetti C. A Theoretical Model of Resource-Oriented Music Therapy with Informal Hospice Caregivers during Pre-Bereavement. J Music Ther. 2018 Mar 9;55(1):27–61.

18. Tamplin J, Clark IN, Lee Y-EC, Baker FA. Remini-Sing: A Feasibility Study of Therapeutic Group Singing to Support Relationship Quality and Wellbeing for Community-Dwelling People Living With Dementia and Their Family Caregivers. Front Med. 2018 Aug 31;5(AUG):1–10.

19. Teut M, Dietrich C, Deutz B, Mittring N, Witt CM. Perceived outcomes of music therapy with Body Tambura in end of life care – a qualitative pilot study. BMC Palliat Care. 2014 Dec 7;13(1):18.

20. Young L, Pringle A. Lived experiences of singing in a community hospice bereavement support music therapy group. Bereave Care. 2018 May 4;37(2):55–66.

21. Denk JG. The Impact of a Music Therapy Support Group on Perceived Stress, Anxiety, and Depression in Long-Term Caregivers: A Pilot Study. Music Ther Perspect. 2022 Jul 6;1–9.

22. Klein CM, Silverman MJ. With love from me to me: Using songwriting to teach coping skills to caregivers of those with Alzheimer's and other dementias. J Creat Ment Heal. 2012;7(2):153–64.

23. Mittelman MS, Papayannopoulou PM. The Unforgettables: A chorus for people with dementia with their family members and friends. Int Psychogeriatrics. 2018;30(6):779–89.

24. Thompson Z, Tamplin J, Clark I, Baker F. Therapeutic Choirs for Families Living with Dementia: A Phenomenological Study. Act Adapt Aging. 2022 Mar 27;00(00):1–35.
